# Development of guidelines for school staff on supporting students who self-harm: a Delphi study

**DOI:** 10.1186/s12888-022-04266-7

**Published:** 2022-09-29

**Authors:** Inge Meinhardt, Tania Cargo, Ben Te Maro, Linda Bowden, Sarah Fortune, Sasha Cuthbert, Susanna James, Riley Cook, Tania Papalii, Korotangi Kapa-Kingi, Mariameno Kapa-Kingi, Annabelle Prescott, Sarah Elisabeth Hetrick

**Affiliations:** 1 School of Psychology, Faculty of Science, University of Auckland, Science Centre, Building 302, Level 2, 23 Symonds Street, Auckland Central, Auckland, 1010 New Zealand; 2 Department of Psychological Medicine, Faculty of Medical and Health Sciences, University of Auckland, Auckland, New Zealand; 3 A Better Start, E Tipu E Rea (Grant Number 15-02688), National Science Challenge, Auckland, New Zealand; 4 Clinical Advisory Services Aotearoa, Auckland, New Zealand; 5 Social and Community Health, Population Health, Faculty of Medical and Health Sciences, University of Auckland, Auckland, New Zealand; 6 Department of Psychological Medicine, Otago Medical School, University of Otago, Dunedin, New Zealand; 7 School of Psychology, Faculty of Environmental and Life Sciences, University of Southampton, Southampton, UK; 8 Northland District Health Board, Whangarei, New Zealand; 9 Iwi Māori Leader, Taitokerau Muriwhenua, Northland, New Zealand; 10 Centre of Excellence in Youth Mental Health, The Centre for Youth Mental Health, University of Melbourne, Melbourne, Australia

**Keywords:** Self-harm, School staff, Education, Guidelines, Best-practice

## Abstract

**Objective:**

Self-harm is a major public health issue that significantly impacts communities, making early intervention and prevention paramount in addressing this public health issue. This study aimed to develop evidence-based, culturally responsive, safe, and practical guidelines to assist school staff in effectively supporting students who self-harm.

**Methods:**

This Delphi study comprised of a five-step process, oversighted by a Rōpū Mātanga Māori (Māori clinical and cultural governance group), and drawing on the expertise and knowledge gained from existing literature, interviews with stakeholders, and two panels of experts (youth and stakeholders). The Rōpū Mātanga Māori ensured accountability to the principles of Te Tiriti o Waitangi (Treaty of Waitangi) and kept Māori processes central to the research aims. The panels completed two rounds of questionnaires, rating their endorsement of each statement. Statements rated as *important* or *essential* by 80% or more of both panels and Māori participants were included in the final guidelines. The Rōpū Mātanga Māori reviewed any remaining statements to determine inclusion.

**Results:**

Following the five-step process, 305 statements were included in the guidelines. These statements provided guiding actions that endorsed communication, collaborative responsibility, and wellbeing and a student-centred approach.

**Conclusion:**

The guidelines provide guidance to all school staff that is culturally responsive and safe, consensus-based, and evidence-based. It is informed by the voices and experiences of young people and those who support them.

**Supplementary Information:**

The online version contains supplementary material available at 10.1186/s12888-022-04266-7.

## Introduction

Self-harm is a prevalent public health issue affecting individuals, their families, and the community. Self-harm is defined as all acts of self-poisoning (e.g., intentional drug overdose) or self-injury (e.g., self-cutting), regardless of suicidal intent or motivation [1]. Henceforth, self-harm refers both to self-injury and poisoning with an intent to die (often called suicide attempts or suicidal behaviour) and self-injury without intent to die (often called non-suicidal self-injury) [1].

Young people represent one-quarter of those hospitalised due to self-harm in New Zealand [2]. The prevalence estimates of self-harm vary depending on the sampling frame, definition, and measurement used [3]. Studies in New Zealand have found that the 12-month prevalence of self-harm amongst young people (26 years old or younger) can be as high as 30% [4–9].

A repeated cross-sectional survey of New Zealand secondary school students called the Youth 2000 survey series provides insight into self-harm in secondary school-aged young people and highlights inequity in mental health and wellbeing outcomes for Māori in New Zealand [8, 9]. These surveys focus on health, wellbeing, and service access. Most recent data measured the construct of suicide attempts and showed that rates have increased from 4.2% (2007) to 6.2% (2019) [9]. For rangatahi (Māori young people), suicide attempts increased from 7.2% (2007) to 12.5% (2019) [9]. Data on self-harm for the 2019 survey is not yet available. However, in 2012, nearly one-third (28.7%) of Māori young people reported they had self-harmed in the previous 12 months, and 18.7% reported thinking of suicide [8]. In comparison, 24% of their non-Māori peers engaged in self-harm, and 15.7% had thoughts of suicide [8]. Overall, the higher rates of self-harm among Māori young people in New Zealand reflect the impacts of historical and ongoing experiences of colonisation on health and wellbeing outcomes for Māori [10]. Therefore, addressing how systems contribute to and perpetuate inequity is essential to addressing the impact of self-harm.

Self-harm is associated with an increased risk of repeated self-harm, mental health difficulties, substance use issues, and lower educational and employment outcomes [11–13]. When harm occurs due to self-harm, hospitalisation and medical intervention may be needed [12, 14]. An intervention study looking at emergency department admissions amongst Māori (17 years old and older) in New Zealand found that as many as 40% of Māori re-present to the hospital with another episode of self-harm within the following year, which is almost double the rate of non-Māori [15]. Self-harm is also associated with a greater risk of suicide [16]. Recent estimates suggest that young people hospitalised due to self-harm are thirty times more likely to die by suicide in the year following hospitalisation than the general population [17]. The relationship between self-harm and suicide is particularly concerning, considering that suicide is one of the leading causes of death in young people in New Zealand [18]. Māori young people are disproportionally negatively affected by suicide [19]. Between 2002 and 2012 the rate of Māori young people dying by suicide ranged from 18.8 per 100,000 to 38.4 per 100,000, compared to 7.2 per 100,000 to 10.7 per 100,000 for non-Māori, non-Pacific children and young people [19].

Given the evidence about the adverse outcomes of self-harm, including the high rates of death by suicide in New Zealand young people [6], it is evident that early intervention and prevention of self-harm is key to ensuring better health and life outcomes for young people. Considering the equity gap in the rates of self-harm and suicide between Māori and non-Māori, these interventions must be culturally responsive and safe for Māori young people and their families.

Studies examining self-harm prevention within Māori communities have predominantly been on Māori adults [15, 20], focusing on cultural interventions [21, 22]. These studies, along with studies examining the issue of self-harm in rangatahi, highlight the importance of connectedness to family, culture, community, and iwi (tribe) in enhancing Māori mental wellbeing [23–25]. Importantly, this includes connection to the school community and school-based groups such as kapa haka (Māori performing group) [23].

The school environment is an ideal space where intervention and prevention of self-harm and suicide can occur [26, 27]. Schools are where most young people spend their time and can serve as a source of support and stress for students [12]. Young people with higher absenteeism or who have dropped out of school, present with higher rates of self-harm [28–30]. This association between school engagement and self-harm highlights the increased vulnerability of young people who are not engaged in school and the need for associated support and interventions for this group of young people. It also highlights the importance of the school environment in effectively supporting students who self-harm. Young people in New Zealand want to receive wellbeing support from their school community [31]. Furthermore, school staff play an essential role in formally and informally supporting students and their families [26].

A recent study highlighted the challenges New Zealand school staff face in supporting students who self-harm [32]. Pastoral care staff reported a perceived increase in distress in young people, resulting in increased workload (i.e., more students needing support) and a lack of resources [32]. They reported feeling overwhelmed and ill-equipped to support these students. The findings highlighted the lack of consistency in practice and responses between individual staff members within and between schools in New Zealand [32]. Guidelines can serve as a tool to address these issues and help to establish consistent practices within the school environment; they are generally viewed favourably by pastoral care staff in New Zealand [32].

To date, there are no New Zealand specific guidelines on how school staff should support students who self-harm. A suicide prevention toolkit exists [33], primarily focused on how schools should respond to suicide in their school community. This toolkit does not guide school staff in preventing and early intervention of self-harm. There are international guidelines and recommendations; however, these do not address specific cultural considerations for New Zealand school students and focus predominantly on non-suicidal self-injury [34–36]. Furthermore, despite the usefulness of guidelines, researchers in New Zealand found that none of the guidance counsellors interviewed in their qualitative study utilised the available international guidelines to inform their practice [37].

Given the inequity that exists in the rates of self-harm between Māori and non-Māori, and the responsibilities outlined in Te Tiriti o Waitangi (Treaty of Waitangi); any guidelines for New Zealand need to be developed in a way that is consistent with the bi-cultural principles documented in Te Tiriti o Waitangi [38, 39]. Guidelines must be relevant to the communities it aims to serve, evidence-based and provide behaviourally-specific recommendations to increase the likelihood of implementation [40]. This is particularly important when considering the incongruence between the reported need for guidelines in New Zealand schools [32, 37] and the reported lack of uptake and implementation of the available international guidelines in New Zealand schools [37].

In response to the need outlined above, this project aimed to develop consensus-based guidelines for schools to guide school staff’s responses when supporting students who self-harm (irrespective of suicidal intent).

## Methods

The study used the Delphi method, a well-established consensus-based research methodology that establishes shared agreement around a research question using expert panels [41, 42]. The Delphi method has been increasingly popular in mental health research and developing guidelines for self-harm and suicidal behaviour [43–47]. This methodology, allows for incorporating the knowledge and expertise of consumers and providers in developing guidelines [41]. Similar to the methods employed in the development of the #chatsafe guidelines [45] and the Mental Health First Aid guidelines for suicidal behaviour [43, 44], the study consisted of five stages: (1) Literature review, (2) Interview transcript review, (3) Questionnaire development, (4) Expert Panel formation, (5) the Delphi Process. Unlike the Mental Health First Aid guidelines developed for supporting Aboriginal and Torres Strait Islanders engaging in non-suicidal self-injury and experiencing suicidal behaviour and thoughts [46, 47], we did not have a separate or stand-alone indigenous panel. We were not aiming to develop a guidelines specifically and exclusively for Māori, but a guidelines that was responsive to Māori, we modelled our approach, on the methodology employed by a bicultural Delphi study on the development of mental health nursing clinical indicators in New Zealand, who had equal representation of Māori and non-Māori panel members  [48]. In order to ensure responsiveness to Māori, the perspectives of Māori and Māori young people were incorporated from the outset and throughout the Delphi process.

Unlike other Delphi studies, we also engaged the Rōpū Mātanga Māori (Māori clinical cultural governance group), who oversaw and had input at all levels of the process across these five stages, and made final decisions on any issues where there were differing opinions, ensuring culturally responsive processes.

The Rōpū Mātanga Māori consisted of a) a Māori young person, b) two clinicians with significant experience working in child and adolescent mental health services and of working with young people who engage in self-harm, c) two members working in youth focused community-driven grassroots suicide prevention initiatives, d) and a further member with experience in a role where they held responsibility and oversight of suicide prevention within a health district. The Rōpū Mātanga Māori ensured that the Te Tiriti O Waitangi principles were upheld, which ensured that this was a Māori-centred piece of research [38, 39]. The Rōpū was able to utilise traditional practices of karakia (prayer), whakawhanaungatanga (the process of establishing relationship), kai (food), and korero (conversation) within a contemporary non-Māori environment. Te Tiriti O Waitangi helps build a strong foundation and ensures that any health provision caters for Māori as equal partners, ensuring that Māori are protected and can participate fully in the research. Although not Kaupapa Māori research, this study nevertheless valued the Rōpū for their cultural expertise and governance so that Māori participants were protected in the study. The inclusion of Māori researchers further supported a Te Tiriti O Waitangi aligned research study.

This study was approved by the University of Auckland Human Participants Ethics Committee (Ref: 023702).

### Literature review

A literature review was led by one of the co-authors (LB) and conducted by LB and members of the wider research team. The literature search aimed to identify documents containing statements relating to recommendations relevant to how school staff can support students who self-harm.

Included documents were in English and focused on managing self-harm in young people between the ages of 5 and 19 years within the school environment. Documents were excluded if they focused on: (a) school-based services or programmes that were related to general mental health (i.e., self-harm was not the target or considered along with other targets), (b) suicide postvention, interventions and strategies, (c) other risk behaviours in young people, (d) general psychoeducation on self-harm, (e) a population group exclusively outside of the age range (5–19 year olds) or not relevant to the school context. The reviewed documents were academic peer-reviewed literature, online grey literature, and additional literature recommended by the professional network of the research group at the time of the literature review.

#### Academic peer-reviewed literature search

PsychINFO, OVID, MEDLINE, and EMBASE databases were searched for articles published in English between 1990 and the 1st of April 2019. The search terms were: (a) the target population (youth, adolescent, young adult, teenage, children), (b) school settings (primary school, secondary school, high school, elementary, junior high school, middle school) and (c) self-harm descriptors (auto-mutilation, drug overdose, suicidal behaviour, self-injurious behaviour, overdose, self-destructive behaviour, attempted suicide, suicide, suicide prevention, suicidality, self-cutting, self-mutilation, self-poisoning, self-harm, suicidal ideation).

The initial search identified 1081 articles, of which 978 were excluded after the screening of titles and abstracts. The full text of 110 articles was retrieved and reviewed by three authors (IM, LB, SJ) for inclusion. An additional 11 articles were excluded during this final screening, resulting in 99 included articles. Of these, 66% were from the USA. Regarding study type, 26% were quasi-experimental intervention studies, 24% were review articles, 23% were association studies (mainly cross-sectional surveys), 14% were qualitative studies, and 12% were randomised control trials of interventions.

#### Grey literature search

The search for grey literature used the Google platforms for the United States (google.com), Australia (google.com.au), New Zealand (google.co.nz), and the United Kingdom of Great Britain and Northern Ireland (google.co.uk). After an iterative process to determine the most appropriate search terms to produce relevant results, the search phrase “school guideline self-harm” was used. The first 20 search results on each Google search platform were used to target the most relevant documents. This number was chosen as it identified the most relevant documents, because the relevance of websites identified beyond 20 decreased.

The initial search identified 79 documents. After removing duplicates and screening documents for inclusion, 23 documents were retrieved. Four additional documents were excluded in the final screen, resulting in 19 documents in the final review. The documents were published between 2010 and 2018, and 37% were from England. Regarding grey literature type: 58% were guidelines, 16% were online articles, 16% were webpages, 5% were policy documents, and 5% were evaluations. An example of a webpage found and included was the Mental Health Foundation (New Zealand) page on self-harm (see https://mentalhealth.org.nz/conditions/condition/self-harm). An example of guidelines found and included was the Royal Australian and New Zealand College of Psychiatrists clinical practice guidelines for managing deliberate self-harm (see https://www.ranzcp.org/files/resources/college_statements/clinician/cpg/deliberate-self-harm-cpg.aspx).

#### Additional literature provided by the professional network

During the literature search and data extraction, colleagues from the research team’s professional network recommended six additional documents. These were published between 2000 and 2019, and 50% were from the USA. Regarding document type, 50% were guidelines, 16.7% were literature reviews, 16.7% were position papers, and 16.7% were government national survey reports.

#### Data extraction

Four authors (IM, SJ, LB, RC) undertook data extraction of statements relevant to how school staff can support students who self-harm from documents retrieved in the literature search.

### Interview transcripts review

In 2018, an interview study involving school staff was conducted [32]. The interviews aimed to explore their experience and current practice in managing self-harm in schools. The transcripts of all the interviews undertaken were reviewed for the Delphi study. The interviews were undertaken by five researchers, two of which were Māori the remaining three were non-Māori. Overall, 32 participants were interviewed; 24 were school guidance counsellors, six were nurses, one was a social worker, and one was a chaplain. One participant identified as Asian, one as Pacific Peoples, and the remaining as European.

#### Data extraction

Two authors (IM and SJ) undertook data extraction from the transcripts. During a pilot phase, statements from 15% of the interviews were identified by both researchers to ensure consistency.

### Questionnaire development

Five of the authors, two of which were Māori and members of the Rōpū Mātanga Māori (BTM, SC) and three others (IM, LB, SH) met to ensure that statements that were extracted from the literature and interviews were consistent in language, avoided repetition and were relevant to school staff and practices within New Zealand schools. Members of the Rōpū Mātanga Māori (BTM, SC, TP, MKK) ensured the statements were appropriate and relevant to Māori students and their families.

This resulted in statements phrased as: what the target behaviour was, who should do it, how they should do it, and when. Notably, the meaning of the original extracted statements was retained to reflect the diversity of beliefs, views, and opinions evident across the data sources. This translation of items into specific target behaviour statements is fundamental to increasing the likelihood of implementation [40].

### Delphi panel formation

Participants were recruited into one of two Delphi panels: Stakeholders or Youth. Participants had to be over 16 years of age and live in New Zealand to participate. The minimum age requirement was set for ethics approval. The stakeholder panel consisted of members with expertise and knowledge of the New Zealand school and health system, and knowledge of managing students/young people who engage in self-harm. The youth panel consisted of members with expertise in advocating for young people. Stakeholder panel members were eligible to participate in the study if they were: 1) at least 18 years of age, 2) lived and worked in New Zealand, 3) and were employed as either a school staff member, a healthcare professional or a researcher working with young people or specialising in the management of self-harm, or an education policy or decision maker. Youth panel members were eligible to participate in the study if they were: 1) aged between 16 and 25 and 2) current members of a youth advisory group within New Zealand.

Participants were recruited by emailing professional networks of the research group and schools. The youth panel consisted of participants between the ages of 16 and 25 years. Youth is a broad age range, and for the current study being at school (or school aged) was not a requirement given that school attendance would have been recent enough for participants in this age range to recall and reflect on their experiences. The youth panel was recruited via email and a Facebook advertisement targeting youth advisory groups in New Zealand. Participants were sent the online questionnaire, a participant information sheet, and an online consent form, following expressions of interest. Informed written consent was required before participants were able to access the questionnaire. All participants were given the opportunity to be acknowledged as contributors to the final guidelines and offered a certificate of participation. The youth panel was offered an additional gift voucher.

#### Panel members

Table 1 shows a detailed account of the demographic characteristics of panel members. In the first round, 64 panel members (30 youth and 34 stakeholders) participated, and 48 (21 youth and 27 stakeholders) participated in the second round. Participants were from across New Zealand and represented various age groups (See Table 1). Using the level one prioritised ethnicity protocol provided by Ministry of Health [49], 28.1% identified as Māori, 18.8% as Pacific Peoples, 6.3% as Asian, and 46.9 as European.

**Table 1:** Demographics of participant by panel and across panels

Demographics	Youth		Stakeholders		Both Panels	
	n	%	n	%	n	%
Gender						
Female	24	80.0	22	64.7	46	71.9
Male	5	16.7	12	35.3	17	26.6
Trans woman	1	3.3	–	–	1	1.6
Age						
16 to 18 years	20	66.7	–	–	20	31.3
19 to 25 years	10	33.3	1	2.9	11	17.2
26 to 29 years	–	–	4	11.8	4	6.3
30 to 44 years	–	–	13	38.2	13	20.3
45 to 59 years	–	–	14	41.2	14	21.9
60 and over	–	–	2	5.9	2	3.1
Ethnicity (Level 1 Prioritised)						
Māori	10	33.3	8	23.5	18	28.1
Pacific Peoples	4	13.3	8	23.5	12	18.8
Asian	3	10.0	1	2.9	4	6.3
European	13	43.3	17	50.0	30	46.9
Region in Aotearoa						
Northland	–	–	5	14.7	5	7.8
Auckland	8	26.7	13	38.2	21	32.8
Waikato	–	–	1	2.9	1	1.6
Bay of Plenty	7	23.3	1	2.9	8	12.5
Taranaki	3	10.0	–	–	3	4.7
Gisborne	–	–	1	2.9	1	1.6
Manawatu-Wanganui	–	–	1	2.9	1	1.6
Wellington	3	10.0	4	11.8	7	10.9
Tasman	2	6.7	1	2.9	3	4.7
Nelson	5	16.7	–	–	5	7.8
Canterbury	1	3.3	3	8.8	4	6.3
Otago	1	3.3	4	11.8	5	7.8

Table 2 outlines the participants’ range of experience and roles, including the professional roles, characteristics of the school type they are affiliated with, and their experience with self-harm. Of note, 50% of the youth panel and 21% of the stakeholder panel reported lived experiences of self-harm.

**Table 2:** Overview of the roles and experiences of participants by panel and across panels

Experiences	Youth		Stakeholders		Both Panels	
	n	%	n	%	n	%
Roles relating youth and self-harm						
Secondary School Student	9	30.0	–	–	9	13.8
Youth Group Member	30	100.0	5	14.7	34	52.3
Parent of school-aged child	–	–	3	8.8	3	4.6
Researcher	–	–	6	17.6	6	9.2
School Guidance Counsellor	–	–	4	11.8	4	6.3
Deputy Principal	–	–	2	5.9	2	3.1
Principal	–	–	4	11.8	4	6.3
Teacher	–	–	1	2.9	1	1.6
Youth Worker	–	–	1	2.9	1	1.6
Student Adviser	–	–	1	2.9	1	1.6
School Nurse	–	–	1	2.9	1	1.6
Educational Psychologist	–	–	1	2.9	1	1.6
Youth Health Clinic Provider	–	–	1	2.9	1	1.6
Policy or Education Decision Maker	–	–	4	11.8	4	6.2
Suicide Prevention Professional	–	–	6	17.6	6	9.2
Community Mental Health Professional	–	–	7	20.6	7	10.8
School Characteristics						
Primary school	–	–	8	23.5	8	12.3
Intermediate	–	–	5	14.7	5	7.7
Secondary School	12	40.0	13	38.2	25	38.5
Rural	3	10.0	5	14.7	8	12.3
Urban	4	13.3	5	14.7	9	13.8
Single-sex	6	20.0	6	17.6	12	18.5
Co-ed	5	16.7	7	20.6	12	18.5
State	6	20.0	10	29.4	16	24.6
Integrated	2	6.7	4	11.8	6	17.6
Private	2	6.7	2	5.9	4	6.2
Tertiary Education	6	20.0	–	–	6	9.2
Experience with Self-Harm						
None	2	6.7	1	2.9	3	4.6
Lived Experience	15	50.0	6	17.6	21	32.3
Known someone who self-harmed	22	73.3	26	76.5	48	73.8
Supported someone who self-harmed	19	63.3	28	82.4	47	72.3

### Delphi process

Participants completed two rounds of questionnaires (see supplemental files 1 and 2) online using Qualtrics [50]. Data from these questionnaires were hosted on the University of Auckland secure network. Both questionnaires were available for completion over 10 weeks. The second questionnaire was released 4 weeks after the closure of the first. Five weeks after each questionnaire, participants were emailed reminders. Both the questionnaires were released and completed during the Covid-19 pandemic in 2020 and the associated lockdowns in New Zealand that year.

Panel members rated each target behaviour statement based on whether they believed school staff should or should not engage in the behaviours outlined in the statements. The following 5-point Likert rating scale was used; 1-*Should not be included*, 2- *unimportant*, 3- *depends/do not know*, 4- *Important*, 5- *Essential.* At the end of each section, there was an open text box, where panel members were asked to provide comments on items, suggestions for new items and changes to existing items. These comments and suggestions were then incorporated as new items in the subsequent questionnaire. Consensus (80% or more) within and across panels was required to be included in the guidelines. Items were retained if 80% of each panel agreed that an item was *essential* (5) or *important* (4). This criterion is consistent with the criterion used in other Delphi studies [41]. See additional file 1 for Questionnaire one.

In the second questionnaire (see additional file 2), participants re-rated statements endorsed by 70% to 79% of panel members, and any new statements that participants suggested in the first questionnaire. Also included for re-rating in the second questionnaire were statements where one panel reached consensus (80% or more), but the other panel did not reach consensus (less than 80%). Based on panel members’ feedback, the rating scale was adjusted in the second questionnaire to separate (3) *depends* and (6) *do not know.* Panel members were given a copy of their ratings for the first questionnaire, and the percentage of panel members who rated statements as essential, *important*, or *unimportant*. Panel members also received a list of retained and excluded statements.

### Rōpū Mātanga Māori

The Rōpū Mātanga Māori (Māori decision-making and governance group) reviewed statements that did not reach consensus (criteria above) following the second questionnaire. The items where there was no consensus between youth and stakeholders, and between Māori and non-Māori panel members were reviewed by the Rōpū Mātanga Māori. They were the kaitiaki (guardians) of the data and decided whether an item should be included in the guidelines. Factors considered in this decision-making process were whether they aligned with both Tikanga Māori and Te Ao Māori (Māori world view and practices) to ensure the cultural and clinical integrity of the items while prioritising the rangatahi Māori voice in the choice of items.

## Results

### Panel agreement

Pearson’s r was calculated using SPSS software [51] to determine the correlations between the Youth and Stakeholder panels’ ratings. For the 308 items rated in Round One, the two panels’ endorsement rates were moderately correlated (*r* = 0.66, *p* < 0.001). Of the total number of Round One statements, there were 76 statements with a discrepancy between the two panels. A discrepancy was defined as when one panel reached consensus to include a statement, but the other panel did not reach consensus to include it. For the 129 items rated in Round two, the two panels’ endorsement rates were weakly correlated (*r =* 0.39, *p* < 0.001). Of the total number of Round Two statements, there were 41 statements with a discrepancy between the panels. An outline of the key areas of discrepancy follows.

#### Information sharing

Both panels agreed that other people involved in the care of the student must be informed of a student’s self-harm. However, the panels disagreed on what information should be shared and who should be notified. The youth panel endorsed statements that encouraged disclosing essential information about a student’s self-harm to other school staff if negotiated and discussed with the student first. In contrast, the stakeholder panel did not agree on items related to this issue.

#### Involvement of family members

Stakeholders endorsed items that support family member involvement in decisions regarding a student’s self-harm (e.g., where to refer). In contrast, the youth panel did not agree on items related to this issue. Instead, they endorsed and encouraged the student's involvement in the decisions that would affect the student. The youth panel endorsed involving family members in developing and implementing support plans and prevention initiatives (e.g., psychoeducation).

#### How to manage triggering educational material

Another discrepancy was on how potentially triggering educational material needs to be managed within the school. Triggering educational material refers to literature and other forms of media, and school curriculum that references self-harm, suicide, or other potentially difficult themes (e.g., sexual abuse). Stakeholders agreed on implementing of processes such as content warnings and check-ins around potentially triggering educational materials; however, the youth panel did not reach consensus on items related to this issue. Generally, youth endorsed items that emphasised the need for students to make the choice themselves. Therefore, supporting the idea of content warnings and the availability of support, but ultimately ensuring students weren’t being censured (i.e., prevented from choosing topics) and had decision making power to opt in or out of engaging with potentially triggering educational material was highlighted as important.

#### Assessment content

With respect to the specific questions that need to be covered in a psycho-social assessment, stakeholders were more particular about the importance of the various steps and topics to be covered in a psychosocial assessment with students who self-harm than the youth panel. 

#### The role of staff in supporting students who self-harm

The panels also disagreed on the extent to which every school staff member can and should play a role in supporting students who self-harm. Youth wanted to approach any staff member within a school, whereas stakeholders did not agree on items related to this issue.

#### Disciplinary responses to self-harm

Within New Zealand schools there are varying ways school staff respond to a student who self-harms while at school. Some schools react to incidents of self-harm by engaging in disciplinary processes, resulting in students being stood down (prevented from attending school for a set period) because of the student’s self-harm. Youth were in favour of abolashing policies and processes within the school, that encouraged these disciplinary processes, which were considered punitive towards students who self-harm (e.g., against students being stood down because they self-harm). Stakeholders did not reach consensus on items related to this issue.

Discrepancies between panels were resolved through the Delphi process and by the Rōpū Mātanga Māori.

### Statement endorsement

**Fig. 1:**
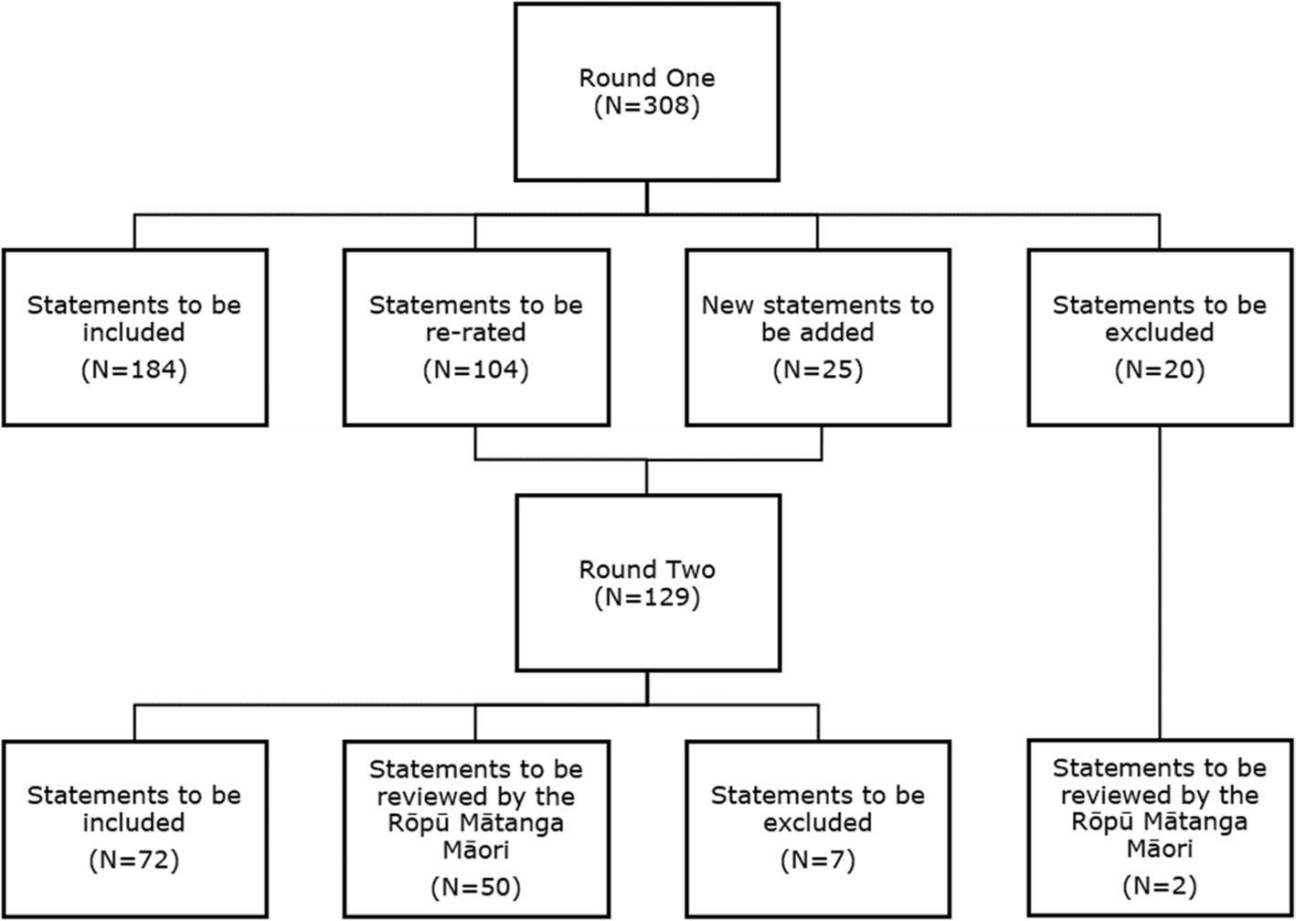
Overview of statements across the two questionnaire rounds

Across the two rounds, 256 statements were endorsed, and 25 were excluded. Figure 1 shows an overview of the endorsement, rejection, and re-rating of items in each phase of the Delphi process. The statements that the panel members in each panel supported collaborative responsibility for student wellbeing within schools, with specific responsibilities for various school staff. Although there was support for a more specific wellbeing team dedicated to supporting students who self-harm, there was also a clear directive for all school staff to have the necessary skills and understanding to sensitively and appropriately respond to a young person who discloses self-harm in a way that validates the young person’s experience, reinforces their help-seeking, and ensures that they gain further support as required. Panel members also supported a designated team with added responsibilities in supporting students who self-harm beyond the support other school staff would provide. Panel members endorsed items that outlined that this team should give immediate support following disclosure or incidents of self-harm, and outlined long-term care the team should provide. Panel members also endorsed items that ensured school staff are provided with the necessary support to care for the wellbeing of school staff.

### Rōpū Mātanga Māori

Consensus between Māori and non-Māori panel members revealed that Māori participants endorsed two items excluded in the first round. These two items, along with the 50 items that did not reach consensus in round two, were presented to the Rōpū Mātanga Māori.

The Rōpū Mātanga Māori reviewed 52 statements (2 where there was no consensus between Māori and non-Māori panel members, 50 where there was no consensus between youth and stakeholders’ panels) to determine whether these should be included, reworded, or excluded. The decision was made to exclude five of the 52 items. Four statements were excluded due to the lack of endorsement by youth panel members. A further statement was deemed irrelevant and excluded during the review. The Māori Rōpu expanded on two existing statements, also included during this decision-making process. The first statement highlighted the importance of noticing a change in a student’s affect and behaviour as a warning sign. The second statement specified how often the designated team must meet. Thus, the review, 305 statements were endorsed for inclusion into the guidelines following review by the Rōpū Mātanga Māori.

### The guidelines

The statements included in the guidelines were divided into five sections that follow a logical and chronological order in which school staff may approach the management of self-harm. The first section provides guidance that ensures everyone works together, clarifying the roles and responsibilities of the various school staff, and the need for policies and procedures to ensure a student-centred and whole school approach. The second section outlines how all school staff must respond to self-harm (historical and in the moment), providing them with steps to follow when a student reaches out or is distressed. The third and fourth section outlines specific directives for a subset of the school staff. We refer to this team as the designated team. The team provides more in-depth support than other school staff might. It also has added responsibilities beyond those of other school staff. The third section outlines the acute responses this designated team would provide, such as debriefing and informing the family. The fourth section outlines how the designated team can support students in the long term, which involves one-on-one support for the student, liaison and collaboration with other important support groups and services to expand the student’s circle of care. The final section outlines essential aspects of ensuring school staff are supported. It outlines basic directives around self-care and emphasises important systems that need to be put in place to prevent burnout and ensure the wellbeing of staff (e.g., adequate leave).

## Discussion

The current study used the Delphi consensus method to co-create evidenced-based guidelines on supporting students who self-harm in New Zealand schools. To the best of our knowledge, this is the first study to utilise this methodology to create guidelines specifically for New Zealand schools, including a broad range of stakeholders with a large proportion of young people.

### Key findings

This study comprised five-steps and produced 305 statements grounded in current literature and expert-based evidence about how schools can support students who self-harm. The Delphi panels consisting of a range of stakeholders, including almost 50% representation of young people and 28% representation of Māori. The Rōpū Mātanga Māori guided it to ensure cultural responsivity and safety, by ensuring accountability to the principles of Te Tiriti o Waitangi and by keeping Tikanga Māori (Māori protocols) and Te Ao Māori (Māori world view) central. The panel members’ diversity in experience and knowledge helped ensure that endorsed statements were evidence-based, practical and promoted cultural safety and wellbeing within schools.

### Endorsed statements

The 305 endorsed recommendations were crafted as behaviourally-specific action items written concisely, separated into five sections that follow the natural management process of students who engage in self-harm in a school. It also provides role-specific guidance for school staff at all levels of the (e.g., the board, leadership team, teaching staff, pastoral care staff, etc.). The guidelines were divided into five sections: 1) How can we work together to support students who self-harm, 2) How do I respond to a disclosure or incident of self-harm, 3) What should the designated team do after an incident or disclosure of self-harm, 4) How can the designated team provide ongoing support to students who self-harmed, 5) How can we support staff who support students. The study findings highlight the importance of ensuring a school context that is supportive, inclusive, and well-being-focused, that acknowledges students’ voices and facilitates a strong connection between students and their families. Overall, the endorsed statements aligned with recommendations in position and guiding document outlining evidence-based recommendations for how schools can respond to students who self-harm [34, 35].

Key to creating guidelines are processes that ensure their implementation into practice. However, implementation poses a significant challenge. One of the weaknesses of guidelines is that they are often not implemented by the people they are aimed at [52, 53]. We engaged in processes that have been recommended to help facilitate implementation by ensuring stakeholder engagement in the co-creation of the guidelines.

### Māori representation and decision-making

As reported above, the study had a bicultural partnership approach embodied in Te Tiriti o Waitangi. This included the pivotal role the Rōpū Mātanga Māori played in ensuring cultural responsiveness throughout the development of the guidelines and equal power for Māori in our panels. This allowed Māori voices to be heard [54, 55] and prioritised in the guidelines to support health equity in New Zealand. Similar to the bicultural approach employed by another New Zealand-based Delphi study [56], we aimed to recruit an even representation of Māori and non-Māori stakeholders and youth. Māori participants represented almost one-third of the total number of participants. However, by determining consensus for Māori and non-Māori participants separately, we ensured equal weighting was given to Māori panel members. As noted earlier, the Rōpū Mātanga Māori had made the final decisions regarding the 52 statements that did not meet consensus.

At the root of inequity sits institutional and systemic racism. Thus, guidelines have the potential to start creating shifts within a given school system by ensuring that prevention and intervention practices not only align with Te Ao Māori (Māori world-view) but also make suggestions on how racism can be addressed. For example, the language around disclosure and involvement with family is intentional, rather than focusing only on parents or caregivers. Therefore, encouraging a collective approach that involves the whole family as mirrored in Māori cultural practices, as opposed to an individualistic approach. Another example is the inclusion of items requiring all school staff to reflect on their cultural values, beliefs, and biases.

### Stakeholder engagement

Along with the prioritisation of Māori voices in developing the guidelines, the extensive range of stakeholders recruited and retained in this study is a significant strength. The stakeholder panel represented different education and health sector levels from across New Zealand. Stakeholder expertise ensured that the included recommendations were feasible and practical within various school environments across New Zealand. Their expertise was valued as much as the academic literature, meaning gaps within the existing literature were highlighted, and practice-based consensus was used to fill these gaps. This was particularly evident via the questionnaire function, allowing participants to comment on their experiences and suggestions for new statements. For example, one participant reflected on their experience as a rural schoolteacher, providing insight into rural schools’ resourcing challenges. This highlights the advantage of this methodology to ensure the engagement of a range of stakeholders and increase the likelihood of implementation by addressing barriers and ensuring buy-in from stakeholders.

Young people are essential stakeholders in developing and implementing guidelines that will impact them. Their involvement in developing mental health and education initiatives is not new. However, it is less common for young people to be involved as panel members in developing mental health-related guidelines. One example is a Delphi study involving a panel of young people and professionals to develop #chatsafe, guidelines for young people on safely communicating online about suicide [45]. We modelled our study on the methodology used to create  #chatsafe [45]. We ensured youth participant voices were given equal weighting as their stakeholder counterparts by determining consensus for the youth and stakeholder panel separately. Furthermore, youth represented almost 50% of the sample. They represented youth from New Zealand with various self-harm experiences and different school contexts.

Including the youth panel allowed us to identify tensions between stakeholders’ worldviews and youth. There was weak to moderate agreement between the two panels across the two rounds, suggesting that although there were some statements that the panels agreed on, there was also a significant number where youth and stakeholder panel members did not agree. There were several areas of contention between the two panels (information sharing, family involvement, managing educational material, assessment, role of school staff and disciplinary processes), which highlighted the importance youth placed on ensuring they had agency over what supports they receive, who provides it and how it is provided. Furthermore, there was some discrepancy between panels on the extent to which prevention and intervention for self-harm should be the responsibility of all school staff. Since self-harm is a public health issue and not exclusively a mental health/health issue [57, 58], it is the responsibility of all school staff, including teachers, to support students who self-harm. Of course, given differences in scope, experience, knowledge and training, the extent of that support may vary. The guidelines acknowledge this by assigning set roles for interventions to specific school staff (e.g., pastoral care) while also ensuring all school staff can signpost appropriate support options and engage in wellbeing enhancing and responsive in crises. Ultimately this ensures that students can be supported by any staff member in their school environment. This is particularly important since young people want their environments to be safe. They want the people around them, including school staff, to support, reach out and look after them [31].

### Limitations

A key component of the work undertaken in this study was that it aimed to develop guidelines specifically for a New Zealand school context. Therefore, it applies predominantly to New Zealand schools. Its applicability to the school contexts in other counties may be limited. Furthermore, the guide was developed for young people in a high school setting (12–19), and panel members in the youth panel were over 16. Therefore, consideration and adaptations to the primary school setting are required, as well as consideration of the perspectives and needs of young people under the age of 16. Concerning panel sizes, 16 participants dropped out after the first round of the questionnaire. Therefore, the consensus reached in the second-round questionnaire was between fewer panel members. Research into consensus ratings in Delphi studies has found that 23 panel members are enough to achieve stable consensus ratings [59]. The number of panel members required in mental health-related Delphi studies is unclear. However, Jorm [41] noted that studies involving fewer than 20 panel members are likely to show less stability. The smallest panel was the youth panel in the second round, with 21 youth panel members. Although the numbers in the youth panel were relatively small, they were sufficient based on previous studies and the assumption of stability in panels with more than 20 members [41, 59]. The attrition rate is expected, because of the questionnaires’ overall length [41]. The literature review used to develop the questionnaire was conducted in 2019, so more recent literature may have been omitted. Panel members may have suggested new statements that may have reflected more recent recommendations found in recent literature on the management of self-harm in schools.

Despite the diversity of experience and backgrounds of our panel members, we could not include all the important voices and perspectives in our panels. In particular, we had a disproportionately higher representation of cis-gender women, which meant we had limited insight into the views of people of other gender identities. Similarly, we had participants from across the country. However, some regions were under-represented or not represented at all. Furthermore, we had only a few participants with teaching experience, and only one current teacher. This limitation in representation highlights the importance of ensuring the involvement of these stakeholders in further evaluation and implementation of the guidelines.

### Future research and implementation

The guidelines must first and foremost address the bicultural partnership of New Zealand school context. Using methodology and frameworks that are culturally relevant, safe and by Māori to address health inequity and stay consistent with a Te Tiriti o Waitangi approach. In addition, the guidelines will need to be evaluated to determine how it is implemented within school environments and their usefulness in supporting school staff (both Māori and non-Māori) to manage self-harm. Furthermore, the usability and applicability of the guidelines within different school contexts need to be understood. A mixed-methodology approach across various schools will provide an understanding of school staff and students’ experiences implementing the guidelines.

As noted previously, understanding the areas of contention between stakeholders and youth highlights barriers to supporting students who self-harm. Therefore, exploring these discrepancies using qualitative methodologies might further our understanding of both groups’ experiences and provide avenues for addressing the disagreement. The implementation strategy for the guidelines will include barrier and facilitator analysis, the development of accompanying resources (e.g., templates) to aid implementation, and collaboration with government agencies that will ensure national dissemination of the guidelines in New Zealand schools. The guidelines are likely to be disseminated via a government agency website.

## Conclusion

Using a multi-step Delphi methodology, we have developed guidelines that could significantly contribute to self-harm and suicide prevention efforts in New Zealand. The diverse experiences of those involved at each step ensure that the voices of stakeholders and young people are acknowledged and heard. The guidelines provide a roadmap of the knowledge, skills and practices that should be incorporated into the lives of school staff to provide culturally responsive and safe, evidence-based, and effective support to students who self-harm.

“Mā te rongo, ka mōhio; Mā te mōhio, ka mārama; Mā te mārama, ka matatau; Mā te matatau, ka ora.”

“Through listening comes awareness; through awareness comes understanding; through understanding comes knowledge; through knowledge comes wellbeing.”

## Supplementary Information

**Additional file 1.**

**Additional file 2.**

## Data Availability

The datasets generated during and analysed during the current study are not publicly available due to limits on availability and use of data outlined and approved by the ethics committee that approved this study. The data may be available from the corresponding author on reasonable request and following approval by the ethics committee.
