# Supplementary material for: Development of guidelines for school staff on supporting students who self-harm: a Delphi study
Source: BMC Psychiatry. 2022 Sep 29;22:631. doi: 10.1186/s12888-022-04266-7 (PMC9520113; doi:10.1186/s12888-022-04266-7)
Supplement: Supplementary file 1 — Additional file 1. [file 12888_2022_4266_MOESM1_ESM.pdf]

Questionnaire introduction and definitions

Questionnaire Information

The questionnaire is divided into sections. Each section contains a number of statements that have come from existing research articles and documents available to school staff that make recommendations on how school staff should support students. We also included statements obtained from interviews with school counselors and nurses in New Zealand schools.

There are over 300 statements in this questionnaire, that cover a range of topics.
Some of these statements may seem controversial or may even contradict, while others might seem obvious or helpful. This reflects the various different recommendations and actions, that are currently available for school staff to use. You may also notice some particular gaps or actions that you believe should be added, please add any suggestion for new or different statements in the open boxes at the end of each section.
Please keep in mind that the final guidelines will need to be adapted to suit each school in which they are used. This includes ensuring cultural responsiveness and ensuring it is suitable for all schools in Aotearoa/New Zealand.

Can I save and return to the questionnaire later?
Your progress will be automatically saved when you click "**Next**" at the end of each section. This means you can complete one or several sections at a time, save it, and come back to it rather than completing it in one session. To return to the questionnaire, simply click on the original link you were emailed.
Please note that this **link will expire after 6 weeks**, which means you have only 6 weeks to complete the survey.

What do I need to do in this questionnaire?
Please rate each statement based on whether you believe the statement should be in the guideline. Thus rate responses based on whether you believe it is appropriate and helpful for school staff, students, and their whānau. Please also use the comments box to give us suggested wording changes for statements, and to let us know if there are statements that should be included that we have missed.

Key terms used:
Whānau: legal guardians, biological family members, and adopted (whangai) family members. Whānau also refers to the group of people who are important to the student.
School staff: any adult employed at the school
Self-harm: Intentional self-injury (including poisoning) that occurs regardless of motivation or degree of suicidal intent

Click "**next**", if you are ready to begin.

Information about you

Information About You

The final guideline will be aimed at all school staff in Aotearoa/New Zealand, and aims to outline how school students in Aotearoa can be supported by school staff, peers and other adults. Therefore, we want to ensure that our sample represents the diversity of our population.

Please answer the following questions:

Do you live in Aotearoa/New Zealand?

- ◯ Yes
- ◯ No

Which age group do you belong to? *(Please note that to be eligible to participate you need to be over 16 years of age)*

- ◯ Under 16 years
- ◯ 16-18 years
- ◯ 19-25 years
- ◯ 26 to 29 years
- ◯ 30 - 44 years
- ◯ 45 - 59 years
- ◯ 60 years and over

Which of the following roles best describes you? *(Please choose all that apply to you, and where requested specify the details of your role)*

- ☐ Rangatahi/young person
- ☐ Current secondary school student
- ☐ Parent of a child in school (currently or in the past)
- ☐ Member of a youth network,council, board or advisory group *(Please specify type of group and organisation; e.g., youth advisory group for local board, etc.)*

[ ]

- [ ] Researcher *(Please specify the specialty area and type of organisation; e.g., suicide prevention research at a university, etc.)*

- [ ] School staff member *(Please specify the title of role; e.g., school teacher, school guidance counselor, etc.)*

- [ ] Policy/education decision maker *(Please specify the title of the role and type of organisation; e.g., education advisor for central government,etc.)*

- [ ] Other *(Please specify the title of the role and type of organisation; e.g., kaitakawaenga for a government agency, suicide prevention co-coordinator for a local district health board, etc)*

Please **choose all** that reflect the characteristics of the school that; you attend (if you are a student), work at (if you are a staff member), or that your child attends (if you are a parent).

- [ ] Primary School
- [ ] Intermediate school
- [ ] Secondary School
- [ ] Rural School
- [ ] Urban School
- [ ] Single-sex school
- [ ] Co-ed School
- [ ] State School
- [ ] Integrated School
- [ ] Private School
- [ ] Other (please specify)
- [ ] This question does not apply to me

Which ethnic group(s) do you belong to? *Mark the space or spaces which apply to you.*

- [ ] New Zealand European
- [ ] Māori
- [ ] Samoan
- [ ] Cook Islands Māori
- [ ] Tongan
- [ ] Niuean
- [ ] Chinese
- [ ] Indian

☐ ☐ Other (Please state: eg, Dutch, Japanese, Tokelauan)

Select the statement(s) that best describes your experience with self-harm?

- ☐ I have no experience or knowledge of self-harm
- ☐ I have lived experience of self-harm
- ☐ I know someone who self-harmed
- ☐ I know someone who is self-harming
- ☐ I have supported someone who self-harmed
- ☐ I am currently supporting someone who self-harms

If you or someone you know are in distress and in need of support, you can call or text the following numbers:

- *If you are in **immediate danger,** please call emergency services immediately on **111***
- *Youthline: call 0800 37 66 33 or free text 234*
- *Need to Talk: Call or text 1737*
- *Lifeline: Call 0800 543 354*
- *Suicide Crisis Helpline: 0508 828 865 (0508 TAUTOKO)*

What is your gender identity?

☐

In which region of New Zealand do you live?

- ○ Northland
- ○ Auckland
- ○ Waikato
- ○ Bay of Plenty
- ○ Gisborne
- ○ Hawke’s Bay
- ○ Taranaki
- ○ Manawatu-Wanganui

- Wellington
- Tasman
- Nelson
- Malborough
- West Coast
- Canterbury
- Otago
- Southland
- Other (Please specify)

School Culture and Environment

School Culture and Environment

The following statements relate to the ways in which the school environment and culture influences **student** wellbeing, how **all school staff** can engage in actions that support the wellbeing of **students**, and ways for creating a supportive school environment.

Key terms introduced in this section:
Senior leadership team: School staff who hold leadership positions within the school, this includes but is not limited to heads of departments, Deans, school principal, deputy principals, Special education needs coordinator (SENCO).

Please rate the following statements:

All school staff must acknowledge **whānau** as an essential and valuable resource.

- Essential
- Important
- Do not know/Depends
- Unimportant
- Should not be included

All school staff must demonstrate meaningful attempts to engage with **whānau**.

- Essential
- Important
- Do not know/Depends
- Unimportant

- ◯ Should not be included

All school staff must demonstrate the school's values, as a way of increasing **student** wellbeing.

- ◯ Essential
- ◯ Important
- ◯ Do not know/Depends
- ◯ Unimportant
- ◯ Should not be included

All school staff must encourage and facilitate **student** help-seeking behaviour, as a way to destigmatise self-harm, suicide and mental health difficulties in general.

- ◯ Essential
- ◯ Important
- ◯ Do not know/Depends
- ◯ Unimportant
- ◯ Should not be included

All school staff must engage in collaborative and transparent communication with each other.

- ◯ Essential
- ◯ Important
- ◯ Do not know/Depends
- ◯ Unimportant
- ◯ Should not be included

The **senior leadership team** and the **principal** must ensure that a school self-review of the school culture and **student** wellbeing occurs routinely. For example, they can use the 'Wellbeing at School' toolkit, which includes surveys for **staff** and **students** to identify the strengths and needs of **school staff** and **students**.

- ◯ Essential
- ◯ Important
- ◯ Do not know/Depends
- ◯ Unimportant
- ◯ Should not be included

The **senior leadership team** and the **principal** must use outcomes from the self-review of school culture and **student** wellbeing to make changes in the school.

- Essential
- Important
- Do not know/Depends
- Unimportant
- Should not be included

All school staff must ensure that **students** are supported to access and meet the **Pastoral Care Team** (e.g., counsellor, social worker, youth worker, psychologist, chaplain, nurse, etc) at any time during school hours (e.g., class time).

- Essential
- Important
- Do not know/Depends
- Unimportant
- Should not be included

All school staff (especially the **senior leadership team** and the **pastoral care team**) must advocate for adequate health care and education for all **students** and their **whānau**.

- Essential
- Important
- Do not know/Depends
- Unimportant
- Should not be included

All school staff must work to identify and value the strengths and abilities of all **students**.

- Essential
- Important
- Do not know/Depends
- Unimportant
- Should not be included

All school staff have a duty of care to all **students**.

- ◯ Essential
- ◯ Important
- ◯ Do not know/Depends
- ◯ Unimportant
- ◯ Should not be included

Please add any suggestions for changes to the statements above, suggestions for new statements, or any other comments or feedback you have.

The Role of School Staff in Supporting Students Who Self-Harm

The Role of School Staff in Supporting Students who Self-Harm

The following statements relate to the various roles and responsibilities of **schools staff** in supporting **students** who self-harm.

Please rate the following statements:

All school staff must identify what their role is in supporting **students** who self-harm by reviewing all related policies, procedures, and guidelines.

- ◯ Essential
- ◯ Important
- ◯ Do not know/Depends
- ◯ Unimportant
- ◯ Should not be included

All school staff must follow and use the related policies, procedures and guidelines to inform how they support **students** who self-harm.

- Essential
- Important
- Do not know/Depends
- Unimportant
- Should not be included

The **board of trustees** must review any guidelines, policies and procedures that influence how **students** who self-harm might be supported.

- Essential
- Important
- Do not know/Depends
- Unimportant
- Should not be included

All school staff must validate **students'** experiences of distress.

- Essential
- Important
- Do not know/Depends
- Unimportant
- Should not be included

All school staff must engage with **students** in a calm and non-judgemental manner.

- Essential
- Important
- Do not know/Depends
- Unimportant
- Should not be included

All school staff must ensure all **students** know that **staff** are there to support and listen to **students** with respect.

- Essential
- Important
- Do not know/Depends
- Unimportant
- Should not be included

The **senior leadership team and the principal** must enforce the expectations outlined in the policies, procedures and guidelines related to self-harm.

- Essential
- Important
- Do not know/Depends
- Unimportant
- Should not be included

The **senior leadership team and the school principal** must ensure **all staff** are aware of their responsibilities and the relevant policy, procedure and guideline documents.

- Essential
- Important
- Do not know/Depends
- Unimportant
- Should not be included

The **senior leadership team and the school principal** must ensure **all staff** are able to follow and perform their roles and responsibilities, by providing access to training, resources, and related supports.

- Essential
- Important
- Do not know/Depends
- Unimportant
- Should not be included

The **senior leadership team and the school principal** must address any incidents where protocol and responsibilities are not followed by **staff.**

- Essential
- Important
- Do not know/Depends
- Unimportant
- Should not be included

In acknowledgement of the fact that school can be a protective factor for students, and the potential risks associated with being alone at home for students who self-harm; the **senior leadership team** (and **other staff** involved) must <u>not exclude/stand-down</u> **students** <u>because</u> they self-harm.

- ◯ Essential
- ◯ Important
- ◯ Do not know/Depends
- ◯ Unimportant
- ◯ Should not be included

Please add any suggestions for changes to the statements above, suggestions for new statements, or any other comments or feedback you have.

Designated Team Responsible for Supporting Students who Self-Harm

Designated Team Responsible for Supporting Students who Self-Harm

<u>All school staff</u> <u>are responsible</u> for the wellbeing of **students**, however, supporting **students** who self-harm involves specific responsibilities, required knowledge and skills.

From here onward the team tasked with the responsibility of supporting **students** who *have self-harmed, are self-harming, or may self-harm in the future (prevention)* will be referred to as the '**designated team**'. This team will have specific responsibilities, and will need to be trained to provide them with the skills and knowledge needed to support distressed **students** and their **whānau**.

We acknowledge that some schools may not be able to have a **team** fully trained and dedicated to supporting **students** who self-harm. It is recommended, that even if only **one staff member** is able to formally be trained, a **group of school staff** should still form part of the **designated team**.

The following statements relate to the steps that need to be taken in order to establish **<u>who</u>** the **designated team** will be.

<u>Please rate the following statements:</u>

The **school leadership team** must choose which **staff members** will be part of the **designated team.**

- ◯ Essential
- ◯ Important
- ◯ Do not know/Depends
- ◯ Unimportant
- ◯ Should not be included

The **school leadership team** must allow any **staff member** to volunteer to be part of the **designated team** based on the **staff member's** interest and suitability for the role.

- ◯ Essential
- ◯ Important
- ◯ Do not know/Depends
- ◯ Unimportant
- ◯ Should not be included

The **designated team**, must include at least **one staff member** from each of the following groups, when possible and if available to the school:

- School Leadership team: for example the principal, member of the senior leadership team, etc.
- School Wellbeing team: for example school Counsellor, Social worker, Chaplain, Nurse, pastoral care team, etc.
- External services: for example general practitioner, youth worker, psychologist, etc.

- ◯ Essential
- ◯ Important
- ◯ Do not know/Depends
- ◯ Unimportant
- ◯ Should not be included

The **designated team** must introduce themselves, where and how to contact them, and their role to all **school staff**, **students** and their **whānau**.

- ◯ Essential
- ◯ Important
- ◯ Do not know/Depends

Unimportant
Should not be included

If possible, all **designated team members** should, ideally, be located in one place, for ease of **student** access. For example by having offices in the same building.

Essential
Important
Do not know/Depends
Unimportant
Should not be included

Please add any suggestions for changes to the statements above, suggestions for new statements, or any other comments or feedback you have.

Policies and Procedures relating to Self-Harm in Schools

Policies and Procedures relating to Self-Harm in Schools

The following statements relate to policies and procedures within schools that relate to self-harm, and how schools can support **students** who self-harm through policies and procedures.

Please rate the following statements:

The **school principal** and the **board of trustees** must develop and implement a school self-harm policy. This should be in collaboration with the **designated team**, and all other **staff** in and outside the school.

Essential
Important
Do not know/Depends
Unimportant

○ Should not be included

The **school principal** must ensure that **all school staff**, **whānau** and **students** are informed of the 'self-harm policy'.

○ Essential
○ Important
○ Do not know/Depends
○ Unimportant
○ Should not be included

Given the link between bullying and self-harm, the **school principal** and **board of trustees** must develop and implement an 'anti-bullying policy' which;

- Defines bullying
- Emphasises a 'no-bullying tolerance' message
- Outlines how any **students** or **staff members** found to participate in bullying will be responded to.

○ Essential
○ Important
○ Do not know/Depends
○ Unimportant
○ Should not be included

The **school principal** must ensure that **all school staff**, **whānau** and **students** are informed of the 'anti-bullying policy'.

○ Essential
○ Important
○ Do not know/Depends
○ Unimportant
○ Should not be included

The **designated team** must lead the development of a written procedure outlining: *What the **school's staff members** can and should do based on their skills, knowledge, experience and workload, and what requires external support/referral.*

○ Essential

- ◯ Important
- ◯ Do not know/Depends
- ◯ Unimportant
- ◯ Should not be included

The **designated team** must lead the development of a written procedure outlining:
*How **all staff** and the **designated person/team** should respond to and support **students** who self-harm.*

- ◯ Essential
- ◯ Important
- ◯ Do not know/Depends
- ◯ Unimportant
- ◯ Should not be included

The **designated team** must lead the development of a written procedure outlining:
*How the **student's whānau** will be informed and involved following a disclosure from or identification of a **student** who self-harms.*

- ◯ Essential
- ◯ Important
- ◯ Do not know/Depends
- ◯ Unimportant
- ◯ Should not be included

The **designated team** must lead the development of a written procedure outlining:
*How and when to refer **students** to external services.*

- ◯ Essential
- ◯ Important
- ◯ Do not know/Depends
- ◯ Unimportant
- ◯ Should not be included

The **principal and board of trustees** must ensure that school disciplinary policies are reviewed and changed; to ensure that **students** who self-harm are not excluded and do not experience disciplinary action as a result of the **student's** self-harming behaviour (i.e. **students** should not be excluded because they self-harm).

- Essential
- Important
- Do not know/Depends
- Unimportant
- Should not be included

If a member of the **senior leadership team** is considering excluding any **student**, a **senior leadership team member** must inform the **designated team**.

- Essential
- Important
- Do not know/Depends
- Unimportant
- Should not be included

If the **designated team** finds out that a **student** with a history of self-harm or who is currently engaging in self-harm might be stood-down or excluded, the **designated team** must; *inform the **principal** that the **student** may be at risk of harming themselves.*

- Essential
- Important
- Do not know/Depends
- Unimportant
- Should not be included

If the **designated team** finds out that a **student** with a history of self-harm or who is currently engaging in self-harm might be stood-down or excluded, the **designated team** must; *ensure the **student** will be safe outside of school, by developing a wellbeing plan with the **student** and their **whānau**.*

- Essential
- Important
- Do not know/Depends
- Unimportant
- Should not be included

The **principal and Board of Trustees** must create a procedural policy that outlines what **school staff** must do when a **student** self-harms at school. This can be done in consultation with the **designated team.**

- ◯ Essential
- ◯ Important
- ◯ Do not know/Depends
- ◯ Unimportant
- ◯ Should not be included

The **principal and board of trustees** must develop and implement a school policy about confidentiality, which outlines; That all **school staff** must ensure that **student** information is protected and kept confidential.

- ◯ Essential
- ◯ Important
- ◯ Do not know/Depends
- ◯ Unimportant
- ◯ Should not be included

The **principal and board of trustees** must develop and implement a school policy about confidentiality, which outlines; <u>*the limits of confidentiality.*</u>

- ◯ Essential
- ◯ Important
- ◯ Do not know/Depends
- ◯ Unimportant
- ◯ Should not be included

The **principal and board of trustees** must develop and implement a school policy about confidentiality, which outlines; <u>*the **expectation** that confidentiality must be broken if a **students** is at risk of harming themselves, being harmed by others, or harming someone else.*</u>

- ◯ Essential
- ◯ Important
- ◯ Do not know/Depends
- ◯ Unimportant
- ◯ Should not be included

The **principal and board of trustees** must develop and implement a school policy about confidentiality, which outlines; <u>*who to go to when confidentiality needs to be broken (i.e., who to inform of the **student's** risk to self, others and from others).*</u>

- Essential
- Important
- Do not know/Depends
- Unimportant
- Should not be included

Teachers (and in some cases **students**) can choose their own teaching materials and resources (e.g. media, literature) that can be inadvertently triggering and distressing to **students**.
Because of this, the **board of trustees** and the **school principal** must develop and implement a school policy and procedure that outlines expectations around choosing teaching material and resources.

- Essential
- Important
- Do not know/Depends
- Unimportant
- Should not be included

Please add any suggestions for changes to the statements above, suggestions for new statements, or any other comments or feedback you have.

Prevention

Prevention

The following statements relate to initiatives, campaigns, programmes and steps that can be taken within the school to prevent self-harm, and the various ways **school staff** can play a role in prevention.

Key term(s) introduced in this section:
Psychoeducation: The delivery of accurate information about an issue or diagnosis, associated short and long–term outcomes, management strategies, and prevention strategies. This information can be delivered to, or aimed at individuals, their whānau, the community or mental health professionals.

Please rate the following statements:

The **board of trustees** and the **senior leadership team** must decide whether self-harm psychoeducation workshops are offered and delivered outside of school time, for the **whānau** of all **students**.

- Essential
- Important
- Do not know/Depends
- Unimportant
- Should not be included

The **senior leadership team** must encourage **teaching staff** to select teaching materials and resources (e.g. media, literature) that **do not** show explicit suicidal and self-harm themes.

- Essential
- Important
- Do not know/Depends
- Unimportant
- Should not be included

The **senior Leadership team** must ensure **teaching staff** provide **students** with trigger warnings and alternative (non-triggering) choices to teaching materials and resources (e.g. Media, literature) if the **staff member** chooses topics or resources that have triggering themes (e.g., self-harm, suicide, trauma, etc).

- Essential
- Important
- Do not know/Depends
- Unimportant
- Should not be included

The **teaching staff** must inform a **designated team member** of their class topic choices that include references to triggering content e.g. self-harm, suicide, etc.

- Essential
- Important
- Do not know/Depends
- Unimportant
- Should not be included

Teachers who choose to use materials that reference triggering content (e.g. self-harm, suicide) must; *inform the **designated team** of what material will be used and when.*

- ◯ Essential
- ◯ Important
- ◯ Do not know/Depends
- ◯ Unimportant
- ◯ Should not be included

Teachers who choose to use materials that reference triggering content (e.g. self-harm, suicide) must; *inform the **whānau** of the **students** that the **teacher** has selected material that may be triggering or distressing for **students**.*

- ◯ Essential
- ◯ Important
- ◯ Do not know/Depends
- ◯ Unimportant
- ◯ Should not be included

Teachers who choose to use materials that reference triggering content (e.g. self-harm, suicide) must; *provide **students** with a warning that the content of the material may be distressing or triggering.*

- ◯ Essential
- ◯ Important
- ◯ Do not know/Depends
- ◯ Unimportant
- ◯ Should not be included

Teachers who choose to use materials that reference triggering content (e.g. self-harm, suicide) must; *provide **all students** with an option to not view or use the material, and must offer alternative material choices to **students** who want to use different material (e.g., a different book that does not include distressing themes).*

- ◯ Essential
- ◯ Important
- ◯ Do not know/Depends
- ◯ Unimportant
- ◯ Should not be included

Teachers who choose to use materials that reference triggering content (e.g. self-harm, suicide) must; *consult with a **designated team member** about how to check-in with **students** before and after the material is used/viewed.*

- ◯ Essential
- ◯ Important
- ◯ Do not know/Depends
- ◯ Unimportant
- ◯ Should not be included

Teachers who choose to use materials that reference triggering content (e.g. self-harm, suicide) must; *complete check-ins with all **students** before and after the material is used/viewed.*

- ◯ Essential
- ◯ Important
- ◯ Do not know/Depends
- ◯ Unimportant
- ◯ Should not be included

Teachers who choose to use materials that reference triggering content (e.g. self-harm, suicide) must; *provide help seeking information (e.g., help-line numbers, websites, etc.) to **students**, after **student's** viewed or engaged with triggering material.*

- ◯ Essential
- ◯ Important
- ◯ Do not know/Depends
- ◯ Unimportant
- ◯ Should not be included

In the event that a **student** chooses to use materials that reference triggering content (e.g. choosing to write a report on a movie that references self-harm) the **teacher** must; *inform a **designated team member** and discuss how the **teacher** can support the **student**. While considering factors such as the **student's** risk and age (i.e., these factors will influence whether whānau are informed, if check in occurs, etc.).*

- ◯ Essential
- ◯ Important
- ◯ Do not know/Depends

◯ Unimportant
◯ Should not be included

In the event that a **student** chooses to use materials that reference triggering content (e.g. choosing to write a report on a movie that references self-harm) the **teacher** must; *inform the **student's whānau** that their children may be using self-selected materials that may be distressing for the **student**.*

◯ Essential
◯ Important
◯ Do not know/Depends
◯ Unimportant
◯ Should not be included

In the event that a **student** chooses to use materials that reference triggering content (e.g. choosing to write a report on a movie that references self-harm) the **teacher** must; *provide **students** with a warning that the content of the self-selected material (book, movie, poem, play, etc.) may be distressing or triggering.*

◯ Essential
◯ Important
◯ Do not know/Depends
◯ Unimportant
◯ Should not be included

In the event that a **student** chooses to use materials that reference triggering content (e.g. choosing to write a report on a movie that references self-harm) the **teacher** must; *provide **students** with suggestions for different materials, but NOT force the **student** to choose a different option.*

◯ Essential
◯ Important
◯ Do not know/Depends
◯ Unimportant
◯ Should not be included

In the event that a **student** chooses to use materials that reference triggering content (e.g. choosing to write a report on a movie that references self-harm) the **teacher**

must; *consult with a **designated team member** about how to check-in with the **student** before and after the self-selected material is used/viewed.*

- Essential
- Important
- Do not know/Depends
- Unimportant
- Should not be included

In the event that a **student** chooses to use materials that reference triggering content (e.g. choosing to write a report on a movie that references self-harm) the **teacher** must; *complete check-ins with the **student** before and after the self-selected material is viewed/used.*

- Essential
- Important
- Do not know/Depends
- Unimportant
- Should not be included

In the event that a **student** chooses to use materials that reference triggering content (e.g. choosing to write a report on a movie that references self-harm) the **teacher** must; *provide help seeking information (including local help-line numbers, websites, etc.) to the **student**, after the **student** viewed or engaged with potentially triggering self-selected material.*

- Essential
- Important
- Do not know/Depends
- Unimportant
- Should not be included

All Teaching staff must implement relaxation and mindfulness practices in class time, to help improve the wellbeing of **all students**.

- Essential
- Important
- Do not know/Depends
- Unimportant
- Should not be included

The **designated team** must assess; existing prevention and wellbeing initiatives in the school and evaluate their effectiveness and usefulness (e.g., what is working, who is using it, etc).

- Essential
- Important
- Do not know/Depends
- Unimportant
- Should not be included

The **designated team** must identify; the factors that help and interfere in the effectiveness of current or future prevention strategies (e.g., lack of time, lack of buy-in from **staff**, willingness of **students**, etc.).

- Essential
- Important
- Do not know/Depends
- Unimportant
- Should not be included

The **designated team**, must schedule time during health class to open up conversation about self-harm with **students**.

- Essential
- Important
- Do not know/Depends
- Unimportant
- Should not be included

The **designated team** must develop and implement psychoeducation programmes for **students**, their **whānau**, and **staff** that are in line with the cultural values, needs and worldviews of participants.

- Essential
- Important
- Do not know/Depends
- Unimportant
- Should not be included

Psychoeducation programmes should aim to inform **students**, their **whānau** and **staff** about; *the facts about self-harm and emotional distress.*

- ◯ Essential
- ◯ Important
- ◯ Do not know/Depends
- ◯ Unimportant
- ◯ Should not be included

Psychoeducation programmes should aim to inform **students**, their **whānau** and **staff** about; *where to seek support for oneself and others.*

- ◯ Essential
- ◯ Important
- ◯ Do not know/Depends
- ◯ Unimportant
- ◯ Should not be included

Psychoeducation programmes should aim to inform **students**, their **whānau** and **staff** on; *how to respond to someone showing signs of distress and self-harm.*

- ◯ Essential
- ◯ Important
- ◯ Do not know/Depends
- ◯ Unimportant
- ◯ Should not be included

If the school has a boarding house, the **designated team** must consult with the **boarding house staff** about wellbeing initiatives that can prevent self-harm that could be implemented in the boarding house.

- ◯ Essential
- ◯ Important
- ◯ Do not know/Depends
- ◯ Unimportant
- ◯ Should not be included

The designated team must introduce and facilitate prevention programmes that focus on enhancing wellbeing of **students** by; *addressing issues that are known to contribute to or result in emotional distress (e.g., substance use, bullying, relationship difficulty, trauma, etc.).*

- Essential
- Important
- Do not know/Depends
- Unimportant
- Should not be included

The **designated team** must introduce and facilitate the prevention programmes that focus on enhancing wellbeing of **students** by; *addressing any discrimination, inequity and stigma (based on gender, ethnicity, religion, socio-economic status, sexual orientation, etc.).*

- Essential
- Important
- Do not know/Depends
- Unimportant
- Should not be included

The **designated team** must introduce and facilitate prevention programmes that focus on enhancing the wellbeing of **students** by; *teaching coping strategies.*

- Essential
- Important
- Do not know/Depends
- Unimportant
- Should not be included

The **designated team** must introduce and facilitate prevention programmes that focus on enhancing the wellbeing of **students** by; *supporting the development of emotion identification and emotion regulation skills.*

- Essential
- Important
- Do not know/Depends
- Unimportant
- Should not be included

The **designated team** must introduce and facilitate prevention programmes that focus on enhancing the wellbeing of **students** by; *supporting **students** to connect with their **whānau** and **peers**.*

- ◯ Essential
- ◯ Important
- ◯ Do not know/Depends
- ◯ Unimportant
- ◯ Should not be included

The **designated team** must introduce and facilitate prevention programmes that focus on enhancing the wellbeing of **students** by; *supporting **students** to explore their identity, strengths and values.*

- ◯ Essential
- ◯ Important
- ◯ Do not know/Depends
- ◯ Unimportant
- ◯ Should not be included

The **designated team** must introduce and facilitate self-harm psychoeducation and prevention programmes that are:

- Evidence-based or informed
- Culturally-relevant
- Able to be delivered during school time

- ◯ Essential
- ◯ Important
- ◯ Do not know/Depends
- ◯ Unimportant
- ◯ Should not be included

The **designated team**, must proactively inform **whānau** and **school staff**, about: *the difficulties **rangatahi/young people** face.*

- ◯ Essential
- ◯ Important
- ◯ Do not know/Depends
- ◯ Unimportant
- ◯ Should not be included

The designated team, must proactively inform **whānau** and **school staff**, about: *the challenges and benefits of social media.*

- ◯ Essential
- ◯ Important
- ◯ Do not know/Depends
- ◯ Unimportant
- ◯ Should not be included

The **designated team**, must proactively inform **whānau** and **school staff**, about: *the value and importance of **whānau** connection and acceptance.*

- ◯ Essential
- ◯ Important
- ◯ Do not know/Depends
- ◯ Unimportant
- ◯ Should not be included

The **designated team**, must proactively inform **whānau** and **school staff**, about: *how to support the emotional development and regulation of **rangatahi**.*

- ◯ Essential
- ◯ Important
- ◯ Do not know/Depends
- ◯ Unimportant
- ◯ Should not be included

The **designated team** must encourage **students** to form **peer-led groups** that: *tackle issues such as bullying.*

- ◯ Essential
- ◯ Important
- ◯ Do not know/Depends
- ◯ Unimportant
- ◯ Should not be included

The **designated team** must encourage **students** to form **peer-led groups** that; *advocate for inclusion and acceptance of **all students**.*

- ◯ Essential
- ◯ Important
- ◯ Do not know/Depends
- ◯ Unimportant
- ◯ Should not be included

The **designated team** must encourage **students** to form **peer-led groups** that: *aim to enhance awareness of issues **students** face or are passionate about (e.g., mental health).*

- ◯ Essential
- ◯ Important
- ◯ Do not know/Depends
- ◯ Unimportant
- ◯ Should not be included

The **designated team** must advocate for and implement evidence-based screening programmes that aim to identify **students** who self-harm or are at risk of self-harming.

- ◯ Essential
- ◯ Important
- ◯ Do not know/Depends
- ◯ Unimportant
- ◯ Should not be included

The **senior leadership team** must ensure that someone within the school is able to conduct the HEEADSSS (Home, Education, Eating, Activities, Drugs and Alcohol, Suicide and Depression, Sexuality and Safety) assessment, and that a HEEADSSS asessment is completed for **all students** by supporting the **school nurse** or **any other staff member** trained to do the assessment.

- ◯ Essential
- ◯ Important
- ◯ Do not know/Depends
- ◯ Unimportant
- ◯ Should not be included

The **school nurse** must conduct HEEADSSS assessment in year 9 for **all students** as a means of screening for self-harm.

- Essential
- Important
- Do not know/Depends
- Unimportant
- Should not be included

In recognition of the link between self-harm and a number of other factors, the **designated team** must ask **students** if they have or are self-harming if they present with or disclose; *experiencing bullying.*

- Essential
- Important
- Do not know/Depends
- Unimportant
- Should not be included

In recognition of the link between self-harm and a number of other factors, the **designated team** must ask **students** if they have or are self-harming if they present with or disclose; *low mood, depression, anxiety, or any other mental health difficulty.*

- Essential
- Important
- Do not know/Depends
- Unimportant
- Should not be included

In recognition of the link between self-harm and a number of other factors, the **designated team** must ask **students** if they have or are self-harming if they present with or disclose; *substance use.*

- Essential
- Important
- Do not know/Depends
- Unimportant
- Should not be included

In recognition of the link between self-harm and a number of other factors, the **designated team** must ask **students** if they have or are self-harming if they present with or disclose; *trauma experiences.*

- Essential
- Important
- Do not know/Depends
- Unimportant
- Should not be included

In recognition of the link between self-harm and a number of other factors, the **designated team** must ask **students** if they have or are self-harming if they present with or disclose: *grief and loss.*

- Essential
- Important
- Do not know/Depends
- Unimportant
- Should not be included

Please add any suggestions for changes to the statements above, suggestions for new statements, or any other comments or feedback you have.

Quarter of the way

${m://FirstName}, thank you for the time you have taken so far.
You are about a **quarter of the way** through this questionnaire!

Remember, you can take a break after completing each section, and return later to complete the remainder of the questionnaire.

To return to the questionnaire, **use the same link** sent to you in the original email.

Please note that the questionnaire will **expire 6 weeks** after it was sent out.

Click "Next" to continue.

Professional Development: Training

Professional Development: Training

The following section focuses on the training requirements for the professional development of **school staff**, in relation to supporting **students** who self-harm.

Please rate the following statements:

The **senior leadership team** must only recommend and approve external training programmes and workshops (related to self-harm) for **school staff** that are: *school-specific (where applicable).*

- Essential
- Important
- Do not know/Depends
- Unimportant
- Should not be included

The **senior leadership team** must only recommend and approve external training programmes and workshops (related to self-harm) for **school staff** that are: *facilitated by professionals from Aotearoa who understand the needs, realities and issues in Aotearoa.*

- Essential
- Important
- Do not know/Depends
- Unimportant
- Should not be included

The **senior leadership team** must only recommend and approve external training programmes and workshops (related to self-harm) for **school staff** that are: *culturally-relevant.*

- Essential
- Important
- Do not know/Depends
- Unimportant
- Should not be included

The **senior leadership team** must only recommend and approve external training programmes and workshops (related to self-harm) for **school staff** that are: *evidence-based.*

- Essential
- Important
- Do not know/Depends
- Unimportant
- Should not be included

The **senior leadership team** must only recommend and approve external training programmes and workshops (related to self-harm) for **school staff** that are: *endorsed by the local district health board's suicide prevention co-ordinator.*

- Essential
- Important
- Do not know/Depends
- Unimportant
- Should not be included

The **designated team** (once trained themselves) must provide training to **all staff** that is focused on self-harm and wellbeing and outlines; *how to have and manage conversations about self-harm with **students.***

- Essential
- Important
- Do not know/Depends
- Unimportant
- Should not be included

The **designated team** (once trained themselves) must provide training to **all staff** that is focused on self-harm and wellbeing and outlines; *how to respond to a **student** who discloses or shows signs of self-harm.*

- ◯ Essential
- ◯ Important
- ◯ Do not know/Depends
- ◯ Unimportant
- ◯ Should not be included

The **designated team** (once trained themselves) must provide training to **all staff** that is focused on self-harm and wellbeing and outlines; *when and how to meaningfully engage* ***whānau*** *of a* ***student*** *who is engaged in self-harm.*

- ◯ Essential
- ◯ Important
- ◯ Do not know/Depends
- ◯ Unimportant
- ◯ Should not be included

The **designated team** (once trained themselves) must provide training to **all staff** that is focused on self-harm and wellbeing and outlines; *how to support* ***students*** *who self-harm in the classroom.*

- ◯ Essential
- ◯ Important
- ◯ Do not know/Depends
- ◯ Unimportant
- ◯ Should not be included

The **designated team** (once trained themselves) must provide training to **all staff** that is focused on self-harm and wellbeing and outlines; *how to support* ***students****' wellbeing in the classroom.*

- ◯ Essential
- ◯ Important
- ◯ Do not know/Depends
- ◯ Unimportant
- ◯ Should not be included

The **designated team** (once trained themselves) must provide training to **all staff** that is focused on self-harm and wellbeing and outlines; *the evidence-based facts of self-*

<u>harm, in the hopes of reducing stigma and myths surrounding self-harm and mental health.</u>

- ◯ Essential
- ◯ Important
- ◯ Do not know/Depends
- ◯ Unimportant
- ◯ Should not be included

Please add any suggestions for changes to the statements above, suggestions for new statements, or any other comments or feedback you have.

Training: Required Knowledge and Awareness

Training: Required Knowledge and Awareness

The following statements relate to knowledge and awareness that **all staff** need, in order to support **students** who self-harm.
This knowledge and awareness relates directly to self-harm and other factors that are associated with self-harm and the wellbeing of **students**.
<u>Please rate the following statements:</u>

All school staff must have the cultural knowledge and awareness to engage with **students** in a culturally safe way that aligns with the **student** and their **whānau** cultural worldview.

- ◯ Essential
- ◯ Important
- ◯ Do not know/Depends
- ◯ Unimportant
- ◯ Should not be included

All school staff must attend training opportunities that will enhance their knowledge and awareness of; *<u>the signs of self-harm.</u>*

- Essential
- Important
- Do not know/Depends
- Unimportant
- Should not be included

All school staff must attend training opportunities that will enhance their knowledge and awareness of; *the relationship between self-harm and other important related issues and risk factors (e.g., bullying, substance use, trauma, substance use, etc.).*

- Essential
- Important
- Do not know/Depends
- Unimportant
- Should not be included

All school staff must attend training opportunities that will enhance their knowledge and awareness of; *the relationship between self-harm and suicide.*

- Essential
- Important
- Do not know/Depends
- Unimportant
- Should not be included

All school staff must attend training opportunities that will enhance their knowledge and awareness of; *how to respond to **students** who self-harm.*

- Essential
- Important
- Do not know/Depends
- Unimportant
- Should not be included

All school staff must attend training opportunities that will enhance their knowledge and awareness of;
*when and how to meaningfully engage **whānau** of a **student** who is engaged in self-harm.*

- Essential
- Important
- Do not know/Depends
- Unimportant
- Should not be included

The **designated team** must know; *how the mental health system in Aotearoa/New Zealand is structured and how it operates.*

- Essential
- Important
- Do not know/Depends
- Unimportant
- Should not be included

The **designated team** must know; *how to make a referral to community mental health services.*

- Essential
- Important
- Do not know/Depends
- Unimportant
- Should not be included

The **designated team** must know; *the potential barriers in referring and gaining support from other services.*

- Essential
- Important
- Do not know/Depends
- Unimportant
- Should not be included

The **designated team** must be aware of the way in which religion can serve as both a protective and risk factor for **students.**

- Essential
- Important
- Do not know/Depends

- Unimportant
- Should not be included

All school staff must be aware of the way in which the school and classroom environment can influence the wellbeing of **students** (e.g., pressure to succeed academically, lack of cultural safety and sensitivity).

- Essential
- Important
- Do not know/Depends
- Unimportant
- Should not be included

The **designated team members** must have the cultural knowledge and awareness required in order to use interventions and strategies to support **students** who self-harm, in a culturally safe way that is NOT harmful or dismissive of the cultural values and views of the **student** and their **whānau**.

- Essential
- Important
- Do not know/Depends
- Unimportant
- Should not be included

All school staff must be aware of current national and local mental health campaigns that address mental health stigma (e.g. Voices of Hope: Speak Your Mind, Like Minds Like Mine, etc).

- Essential
- Important
- Do not know/Depends
- Unimportant
- Should not be included

As part of health and safety, **all school staff** must be aware of the potential risks and hazards within the school's physical environment in order to minimise and monitor risk in relation to self-harm (e.g., doors that lock, classroom equipment that are poisonous or harmful, etc.).

- Essential
- Important
- Do not know/Depends
- Unimportant
- Should not be included

Teachers and the designated team, must be aware that **teachers** (and in some cases students) can choose their own teaching materials and resources (e.g. media, literature), and that some of these materials and resources can be inadvertently triggering or distressing to **students**.

- Essential
- Important
- Do not know/Depends
- Unimportant
- Should not be included

All school staff must be aware of their own cultural knowledge, values and beliefs, and must use the ongoing support of cultural advisors, and training opportunities to enhance their cultural competence.

- Essential
- Important
- Do not know/Depends
- Unimportant
- Should not be included

The **designated team members** must be aware that the environment and nature influences the wellbeing of **students** and their **whānau**.

- Essential
- Important
- Do not know/Depends
- Unimportant
- Should not be included

All school staff must know and be aware of the guidelines, acts and codes relevant to their role and qualification that should guide their behaviour and decisions. For example **counsellors** have a code of ethics that they must follow.

- Essential
- Important
- Do not know/Depends
- Unimportant
- Should not be included

The **designated team** must be aware of current research on self-harm, and the most effective ways of supporting **students** who self-harm by; *attending evidence-based workshops.*

- Essential
- Important
- Do not know/Depends
- Unimportant
- Should not be included

The **designated team** must be aware of current research on self-harm, and the most effective ways of supporting **students** who self-harm by; *networking with mental-health and self-harm **researchers** and **professionals**.*

- Essential
- Important
- Do not know/Depends
- Unimportant
- Should not be included

The **designated team** must be aware of current research on self-harm, and the most effective ways of supporting **students** who self-harm by: *reading up-to-date literature.*

- Essential
- Important
- Do not know/Depends
- Unimportant
- Should not be included

The **designated team** must be aware of the pressures **teaching staff** experience, by engaging in conversations with their co-workers to better understand the roles and experiences of **teaching staff**.

- Essential
- Important
- Do not know/Depends
- Unimportant
- Should not be included

The **teaching staff** must be aware of the pressures the **designated team** experience, by engaging in conversations with their co-workers to better understand the roles and experiences of the **designated team**.

- Essential
- Important
- Do not know/Depends
- Unimportant
- Should not be included

The **designated team** must be aware of the limits of what they can and cannot do based on their experience, qualification, professional codes and current level of training in self-harm.

- Essential
- Important
- Do not know/Depends
- Unimportant
- Should not be included

The **designated team members** must be aware of how social media can be distressing and triggering for some **students**, and as a result might contribute to self-harm.

- Essential
- Important
- Do not know/Depends
- Unimportant
- Should not be included

The **designated team members** must receive training that will give them the knowledge of what information needs to be shared with **whānau**, **co-workers**, and **external agencies** and **community groups** when a **student** presents with self-harm.

- Essential

- ◯ Important
- ◯ Do not know/Depends
- ◯ Unimportant
- ◯ Should not be included

Please add any suggestions for changes to the statements above, suggestions for new statements, or any other comments or feedback you have.

Training: Required Skills

Training: Required Skills

The following statements relate to skills that **all staff** need in order to support **students** who self-harm. These skills relate directly to self-harm, but may also be associated with other skills that are indirectly related to supporting **students** who self-harm.

Please rate the following statements:

The **designated team** must identify and engage in training opportunities that will provide them with the *skills* needed to: *respond to incidents of self-harm.*

- ◯ Essential
- ◯ Important
- ◯ Do not know/Depends
- ◯ Unimportant
- ◯ Should not be included

The **designated team** must identify and engage in training opportunities that will provide them with the *skills* needed to: *complete a psycho-social assessment.*

- ◯ Essential
- ◯ Important
- ◯ Do not know/Depends
- ◯ Unimportant

○ Should not be included

The **designated team** must identify and engage in training opportunities that will provide them with the *skills* needed to: *identify **students** who may be at risk of self-harming.*

○ Essential
○ Important
○ Do not know/Depends
○ Unimportant
○ Should not be included

The **designated team** must identify and engage in training opportunities that will provide them with the *skills* needed to; *identify **students** who have suicidal thoughts, intent and plans.*

○ Essential
○ Important
○ Do not know/Depends
○ Unimportant
○ Should not be included

The **designated team** must identify and engage in training opportunities that will provide them with the *skills* needed to; *take pre-emptive actions to support **students** at risk of engaging in self-harm (e.g., **students** who struggle with their alcohol and drug use, **students** who are being bullied, etc.).*

○ Essential
○ Important
○ Do not know/Depends
○ Unimportant
○ Should not be included

The **designated team** must identify and engage in training opportunities that will provide them with the *skills* needed to; *understand when and how to refer a **student** to community mental health services.*

○ Essential
○ Important

- ◯ Do not know/Depends
- ◯ Unimportant
- ◯ Should not be included

The **designated team** must identify and engage in training opportunities that will provide them with the *skills* needed to; *manage and support **students** with difficulties related to self-harm (e.g., substance use, mental health difficulties, etc.).*

- ◯ Essential
- ◯ Important
- ◯ Do not know/Depends
- ◯ Unimportant
- ◯ Should not be included

All School staff must receive training to equip them with *skills* to: *discuss emotionally distressing issues with **students**.*

- ◯ Essential
- ◯ Important
- ◯ Do not know/Depends
- ◯ Unimportant
- ◯ Should not be included

All School staff must receive training to equip them with *skills* to: *support a **student** who is acutely distressed.*

- ◯ Essential
- ◯ Important
- ◯ Do not know/Depends
- ◯ Unimportant
- ◯ Should not be included

All School staff must receive training to equip them with *skills* to: *support **students** with identifying coping strategies to use until they are able to receive support from the **designated team**.*

- ◯ Essential
- ◯ Important
- ◯ Do not know/Depends

- Unimportant
- Should not be included

The **school principal** must receive training that will equip them with the *skills* needed to support **school staff** to perform their roles and responsibilities in supporting **students**.

- Essential
- Important
- Do not know/Depends
- Unimportant
- Should not be included

The **designated team** must receive training that will equip them with the *skills* needed to inform the **whānau** of **students**, **co-workers**, and **external agencies** and **community groups** of the relevant information relating to a **student's** self-harming.

- Essential
- Important
- Do not know/Depends
- Unimportant
- Should not be included

In order to effectively support **students**' wellbeing and **students** who self-harm **all school staff** must have the *skills* needed to engage with **students** and their **whānau** in a culturally safe way.

- Essential
- Important
- Do not know/Depends
- Unimportant
- Should not be included

The **designated team** must have the *skills* to use interventions and strategies to support **students** who self-harm and their **whānau**, in a way that is culturally safe.

- Essential
- Important
- Do not know/Depends
- Unimportant

○ Should not be included

The **designated team** must be trained in using screening tools that are evidence-based and useful for identifying risk and protective factors associated with self-harm.

○ Essential
○ Important
○ Do not know/Depends
○ Unimportant
○ Should not be included

Please add any suggestions for changes to the statements above, suggestions for new statements, or any other comments or feedback you have.

Supervision

Supervision

The following statements relate to professional supervision, and how it relates to how **school staff** can support **students** who self-harm.

Key terms introduced in this section:
Professional Supervision: the ongoing process of professional support from a qualified supervisor, that focuses on enhancing knowledge and understanding, and skill relating to professional practice and the ethical requirements of the profession.
Cultural Supervision : A formal supervision relationship that aims to enhance the awareness, knowledge and skills needed to work within the cultural context of students (clients) and their whānaua.
Peer supervision: A formal process where a group of co-workers or professionals who have the same or similar roles meet to discuss professional issues with each other.

Please rate the following statements:

The **designated team** must receive clinical supervision, and use it as an opportunity to consult, seek advice, engage in professional development, reflect on their own practice, and as a means of self-care.

- ○ Essential
- ○ Important
- ○ Do not know/Depends
- ○ Unimportant
- ○ Should not be included

The **designated team** must attend peer supervision, and use it as an opportunity to share knowledge, seek advice and as a form of self-care.

- ○ Essential
- ○ Important
- ○ Do not know/Depends
- ○ Unimportant
- ○ Should not be included

The **designated team** must attend cultural supervision, and use it as an opportunity to consult, seek advice, engage in professional and cultural competence development, and to reflect on their own practice and as a form of self-care.

- ○ Essential
- ○ Important
- ○ Do not know/Depends
- ○ Unimportant
- ○ Should not be included

Please add any suggestions for changes to the statements above, suggestions for new statements, or any other comments or feedback you have.

Documentation

Documentation

The following statements relate to the documentation processes that may be required and necessary to support **students** who self-harm.

Please rate the following statements:

The **designated team** must maintain up-to-date records (i.e. clinical notes) for **students** who self-harm, with details of:

- The psycho-social assessment
- Incidents
- Treatment or intervention plan
- Safety plans
- School support plan

○ Essential
○ Important
○ Do not know/Depends
○ Unimportant
○ Should not be included

The **designated team** must document any contact and consultation with the local child and adolescent mental health services, and other community services and groups.

○ Essential
○ Important
○ Do not know/Depends
○ Unimportant
○ Should not be included

The **designated team** must document any contact and consultation with the **student's whānau**.

○ Essential
○ Important
○ Do not know/Depends
○ Unimportant
○ Should not be included

The **designated team** must document any disclosures and steps taken in response (e.g., informed Oranga Tamariki, or developed safety plan and informed **whānau**).

- Essential
- Important
- Do not know/Depends
- Unimportant
- Should not be included

Please add any suggestions for changes to the statements above, suggestions for new statements, or any other comments or feedback you have.

Halfway There

${m://FirstName}, thank you for the time you have taken so far. You are **halfway** through this questionnaire!

Remember, you can **take a break** after completing each section, and return later to complete the remainder of the questionnaire.

To return to the questionnaire, use the **same link** sent to you in the original email.

Please note that the questionnaire will **expire 6 weeks** after it was sent out.

Click "Next" to continue.

Communication and Collaboration

Communication and Collaboration

The following statements relate to communication and collaboration between **all school staff**, the **student**, their **whānau**, and other relevant services and groups. It outlines the

communication needed in order to support **students** who self-harm, including expectations around information sharing and confidentiality.

Please rate the following statements:

The **designated team** must meet regularly to discuss any concerns about **students**.

- Essential
- Important
- Do not know/Depends
- Unimportant
- Should not be included

The **designated team** must meet regularly to seek advise on how to support particular **students.**

- Essential
- Important
- Do not know/Depends
- Unimportant
- Should not be included

The **designated team** must meet regularly to develop plans on how to support **students** and their **whānau**.

- Essential
- Important
- Do not know/Depends
- Unimportant
- Should not be included

The **designated team** must meet regularly to co-ordinate roles and plans (e.g., who is the most appropriate person to call **whānau,** etc.).

- Essential
- Important
- Do not know/Depends
- Unimportant
- Should not be included

The **designated team** must meet regularly to debrief and to check-in with each other.

- ◯ Essential
- ◯ Important
- ◯ Do not know/Depends
- ◯ Unimportant
- ◯ Should not be included

In instances where there is only one person in the **designated team,** they must; establish a consult team to discuss concerns.

- ◯ Essential
- ◯ Important
- ◯ Do not know/Depends
- ◯ Unimportant
- ◯ Should not be included

In instances where there is only one person in the **designated team,** they must; *establish a consult team to seek advise on how to support particular **students**.*

- ◯ Essential
- ◯ Important
- ◯ Do not know/Depends
- ◯ Unimportant
- ◯ Should not be included

In instances where there is only one person in the **designated team,** they must; *establish a consult team to develop plans on how to support **students** and their **whānau**.*

- ◯ Essential
- ◯ Important
- ◯ Do not know/Depends
- ◯ Unimportant
- ◯ Should not be included

In instances where there is only one person in the **designated team,** they must; _establish a consult team to co-ordinate roles and plans (e.g., who is the most appropriate person to call **whānau**)._

- Essential
- Important
- Do not know/Depends
- Unimportant
- Should not be included

In instances where there is only one person in the **designated team,** they must; *establish a consult team to debrief and to check-in with each other.*

- Essential
- Important
- Do not know/Depends
- Unimportant
- Should not be included

The **designated team** must establish a preferred referral method for internal referrals, and must ensure all staff are aware of this method (e.g., email, complete referral form, call and email, face to face conversation and email, etc).

- Essential
- Important
- Do not know/Depends
- Unimportant
- Should not be included

The **designated team** may have concerns about a **student**, or feel that other **staff members** can support a **student** who self-harms. _If this is the case they must;_

- Work with the **student** to identify what they may need from **other school staff**
- Tell the **student** what information will be passed on and to whom
- Inform only the **staff** who are directly involved with the **student**

- Essential
- Important
- Do not know/Depends
- Unimportant
- Should not be included

The **designated team** may have concerns about a **student**, or feel that **other staff members** can support a **student** who self-harms. If this is the case they must; o*only disclose information related to the support the **student** needs but not detail about issues or events that the **student** has disclosed, unless requested by the **student**.*

- ◯ Essential
- ◯ Important
- ◯ Do not know/Depends
- ◯ Unimportant
- ◯ Should not be included

If the **nurse** is not part of the **designated team,** the **designated team** must ensure the **nurse** or other **school medical staff** are informed of any **students** who may be a risk to themselves.

- ◯ Essential
- ◯ Important
- ◯ Do not know/Depends
- ◯ Unimportant
- ◯ Should not be included

The **designated team**, **medical staff** and **the pastoral care team** must ensure they review confidentiality and its limits with all **students** they come into contact with. They must inform **students** when they will be breaking confidentiality, why, and to who.

- ◯ Essential
- ◯ Important
- ◯ Do not know/Depends
- ◯ Unimportant
- ◯ Should not be included

The **designated team** must, to the best of their ability, create a physical environment and space where they support **students** that facilitates privacy (e.g., soundproof, doors closed, etc.).

- ◯ Essential
- ◯ Important
- ◯ Do not know/Depends
- ◯ Unimportant

○ Should not be included

The relevant **designated team member** must not inform the **whānau** about the self-harm the **student** has engaged in, if it will damage the relationship between the **student** and the **designated team member.**

○ Essential
○ Important
○ Do not know/Depends
○ Unimportant
○ Should not be included

All school staff must inform all **students**, that **staff** cannot keep secrets, but that the **staff** will try to ensure that everything the **student** discusses with them will stay confidential. Except if the **student** discloses risk of harming themselves, risk of being harmed by someone else, or risk of harming someone else, in which case the **staff member** will need to break confidentiality and inform the **designated team**.

○ Essential
○ Important
○ Do not know/Depends
○ Unimportant
○ Should not be included

The **designated team**, **medical and first aid staff**, and **pastoral care staff** must be in communication with each other regarding all **students** under their care, and must ensure **other members of the team** are aware of any risks or concerns. Thus no one should work in isolation.

○ Essential
○ Important
○ Do not know/Depends
○ Unimportant
○ Should not be included

The **designated team** must be able to identify **students** who self-harm and therefore must be aware of all **students** who self-harm.

○ Essential

○ Important
○ Do not know/Depends
○ Unimportant
○ Should not be included

The **designated team** must establish relationships within the local community, which includes but are not limited to:

- **Primary care providers**
- **Iwi providers**
- **Private psychologists, counsellors, mental health professionals**
- **Churches**
- **Marae**
- **Youth services/clubs**
- **Sports Clubs/recreational centres**
- **Child and adolescent mental health service**
- **Non-government organisations**
- **Other social services and organisations**

○ Essential
○ Important
○ Do not know/Depends
○ Unimportant
○ Should not be included

To gain an understanding of the services available in the community, the **designated team** must; *identify potential barriers/challenges (e.g., cost, location, etc.).*

○ Essential
○ Important
○ Do not know/Depends
○ Unimportant
○ Should not be included

To gain an understanding of the services available in the community, the **designated team** must; *determine how the service operates.*

○ Essential
○ Important
○ Do not know/Depends
○ Unimportant
○ Should not be included

In the case that a local district health board community mental health service is required, but is not accessible (e.g., geographically isolated, waitlist, referral criteria, etc), the **designated team** must have established relationships with local services (e.g., Non-government mental health service) that can fulfil the equivalent role.

- ◯ Essential
- ◯ Important
- ◯ Do not know/Depends
- ◯ Unimportant
- ◯ Should not be included

The **designated team** must ensure they inform **students** and their **whānau** that the information shared with an individual **designated team member** might also be shared with other members of the **designated team.** But that the information will be confidential to the team, and therefore will not be discussed with others outside of the **designated** team, unless there are safety concerns.

- ◯ Essential
- ◯ Important
- ◯ Do not know/Depends
- ◯ Unimportant
- ◯ Should not be included

Please add any suggestions for changes to the statements above, suggestions for new statements, or any other comments or feedback you have.

Self-Care

Wellbeing and Self-care

The following statements relate to self-care practices that are required in order for **school staff** to support **students** who self-harm.

Please rate the following statements:

Members of the **designated team** must take care of their wellbeing and seek support as and when necessary, by: *having wellbeing plans in place for themselves.*

- Essential
- Important
- Do not know/Depends
- Unimportant
- Should not be included

Members of the **designated team** must take care of their wellbeing and seek support as and when necessary, by: *attending professional and peer supervision.*

- Essential
- Important
- Do not know/Depends
- Unimportant
- Should not be included

Members of the **designated team** must take care of their wellbeing and seek support when needed, by: *being aware of potential triggers or issues that they might find distressing.*

- Essential
- Important
- Do not know/Depends
- Unimportant
- Should not be included

Members of the **designated team** must take care of their wellbeing and seek support when needed, by: *ensuring they have a work-life balance, and enjoyable activities they engage in outside of work.*

- Essential
- Important
- Do not know/Depends
- Unimportant
- Should not be included

Members of the **designated team** must take care of their wellbeing and seek support when needed, by: *being aware of their own limits.*

- ◯ Essential
- ◯ Important
- ◯ Do not know/Depends
- ◯ Unimportant
- ◯ Should not be included

The **school principal** must provide practical and emotional support for **staff** supporting and working with **students** who self-harm.

- ◯ Essential
- ◯ Important
- ◯ Do not know/Depends
- ◯ Unimportant
- ◯ Should not be included

The **designated team** must have clear systems for how appointments are managed, as a means of taking care of the **designated team members** while supporting **students** who self-harm. For example, ensuring there is time in between seeing **students**, which will create time to debrief and process before seeing another **student**.

- ◯ Essential
- ◯ Important
- ◯ Do not know/Depends
- ◯ Unimportant
- ◯ Should not be included

The **designated team** must maintain professional boundaries when working with **students** in order to take care of their own wellbeing, by: *having clear working hours and days, and only seeing **students** during school hours (thus not during holiday, after school).*

- ◯ Essential
- ◯ Important
- ◯ Do not know/Depends
- ◯ Unimportant
- ◯ Should not be included

The **designated team** must maintain professional boundaries when working with **students** in order to take care of their own wellbeing, by: *providing **students** with after-hours numbers to call, and not providing **students** with their (**designated team member's**) own personal phone numbers.*

- Essential
- Important
- Do not know/Depends
- Unimportant
- Should not be included

The **designated team** must maintain professional boundaries when working with **students** in order to take care of their own wellbeing, by: *ensuring **students** have other support people, so as to enhance the support network of the **student**.*

- Essential
- Important
- Do not know/Depends
- Unimportant
- Should not be included

Please add any suggestions for changes to the statements above, suggestions for new statements, or any other comments or feedback you have.

Initial response: history of self-harm

Initial response: history of self-harm

The following section relates to the roles and responsibilities of various **staff members** and **teams** in the instance where a **student** is identified, who has a history of self-harm (this may include self-harm that has occurred in the last day, week, month, year or years). Thus this refers to the steps **staff** might take if a **student** self-harmed in the past.

For this section "the past" or a "history" refers to any self-harming behaviours

that occurred before the present moment ***and therefore can be minutes, hours, days, months or years in the past.***
Identification of the **student** may occur, through observation or disclosure directly to a **staff member** or through a disclosure by a **peer**.

Please rate the following statements:

If a **peer** informs **staff** of *another* ***student's*** *self-harm history* the **staff member** must; *thank the* ***student*** *for informing them.*

- Essential
- Important
- Do not know/Depends
- Unimportant
- Should not be included

If a **peer** informs **staff** of another **student's** self-harm history the **staff member** must: *ask the* ***peer*** *if they know when the most recent time is that the* ***student*** *has self-harmed.*

- Essential
- Important
- Do not know/Depends
- Unimportant
- Should not be included

If a **peer** informs **staff** of another **student's** self-harm history the **staff member** must; *ask the* ***peer*** *for as much contextual information (e.g., when did this happen, who else knows, etc) that the* ***peer*** *can provide.*

- Essential
- Important
- Do not know/Depends
- Unimportant
- Should not be included

If a **peer** informs **staff** of another **student's** self-harm history the **staff member** must; *ensure the **designated team** of who the **peer** is.*

- Essential
- Important
- Do not know/Depends
- Unimportant
- Should not be included

If a **peer** informs **staff** of another **student's** self-harm history, the **designated team** must; *meet with the **peer** and discuss whether the **peer** needs any support.*

- Essential
- Important
- Do not know/Depends
- Unimportant
- Should not be included

If a **student** discloses to a **staff member** that the **student** has self-harmed in the past (through observation, disclosure or screening), that **staff member** must: *thank the **student** for confiding in them.*

- Essential
- Important
- Do not know/Depends
- Unimportant
- Should not be included

If **any staff member** identifies a **student** who self-harmed (through observation, disclosure or screening), that **staff member** must: *engage with the **student** in a calm and non-judgmental manner.*

- Essential
- Important
- Do not know/Depends
- Unimportant
- Should not be included

If **any staff member** identifies a **student** who self-harmed (through observation, disclosure or screening), that **staff member** must: _avoid asking to see a **student's** self-harm injuries as proof of self-harm, but do ask when the last time was that the **student** self-harmed_

- ◯ Essential
- ◯ Important
- ◯ Do not know/Depends
- ◯ Unimportant
- ◯ Should not be included

If **any staff member** identifies a **student** who self-harmed (through observation, disclosure or screening), that **staff member** must: _offer access to medical attention with explanation as to why (e.g., sometimes the injuries can get infected and need to be cleaned and bandaged)._

- ◯ Essential
- ◯ Important
- ◯ Do not know/Depends
- ◯ Unimportant
- ◯ Should not be included

If the **student** consents to medical attention, the **staff member** must ensure the **student** receives medical intervention as soon as possible, and must ensure the **student** is not left alone.

- ◯ Essential
- ◯ Important
- ◯ Do not know/Depends
- ◯ Unimportant
- ◯ Should not be included

The **first aid and medical staff** must: _address the physical needs of any **student** who presents with self-harm injury (e.g., clean and dress any wounds, etc)._

- ◯ Essential
- ◯ Important
- ◯ Do not know/Depends
- ◯ Unimportant
- ◯ Should not be included

The **first aid and medical staff** must: _assess the severity and the degree of medical intervention needed (e.g., hospital versus simply cleaning and dressing the wound)._

- ◯ Essential
- ◯ Important
- ◯ Do not know/Depends
- ◯ Unimportant
- ◯ Should not be included

If **any staff member** identifies a **student** who has self-harmed (through observation, disclosure or screening), that **staff member** must: _inform the **student** that the school has a protocol, where **students** who self-harm are asked to see the a **designated team member**, as the **designated team member** is a good person to talk to._

- ◯ Essential
- ◯ Important
- ◯ Do not know/Depends
- ◯ Unimportant
- ◯ Should not be included

If **any staff member** identifies a **student** who self-harmed (through observation, disclosure or screening), that **staff member** must; _inform the **designated team.**_

- ◯ Essential
- ◯ Important
- ◯ Do not know/Depends
- ◯ Unimportant
- ◯ Should not be included

If **any staff member** identifies a **student** who self-harmed (through observation, disclosure or screening), that **staff member** must: _follow up with the **designated team** to ensure that the **student** has been seen._

- ◯ Essential
- ◯ Important
- ◯ Do not know/Depends
- ◯ Unimportant
- ◯ Should not be included

Boarding house staff must consult with the **designated team** when a **boarding student** has been identified as engaging in self-harm in the past.

- ◯ Essential
- ◯ Important
- ◯ Do not know/Depends
- ◯ Unimportant
- ◯ Should not be included

Please add any suggestions for changes to the statements above, suggestions for new statements, or any other comments or feedback you have.

Initial response: self-harm in the moment

Initial response: self-harm in the moment

The following section relate to the roles and responsibilities of various **staff members** and **teams** in the instance where a **student** is ***identified while engaging in self-harming behaviour*** e.g. in the class-room. Thus, this section covers actions that may be taken if a **student** is ***actively self-harming in the present moment***. Identification of the **student** may occur, through observation or disclosure directly to a **staff member** or through a disclosure by a **peer**.

Please rate the following statements:

If a **peer** informs **staff** that another **student** is *self-harming in that moment*, the **staff member** must; *thank the **student** for informing them.*

- ◯ Essential
- ◯ Important
- ◯ Do not know/Depends
- ◯ Unimportant
- ◯ Should not be included

If a **peer** informs **staff** that another **student** is self-harming in that moment, the **staff member** must; *ask the **peer** for as much contextual information that the **peer** can provide (e.g., where is the **student** at the moment, what is being used, who else is there, etc.).*

- ◯ Essential
- ◯ Important
- ◯ Do not know/Depends
- ◯ Unimportant
- ◯ Should not be included

If a **peer** informs **staff** that another **student** is self-harming in that moment, the **staff member** must; *ensure the relevant **designated team member** is informed of who the **peer** is.*

- ◯ Essential
- ◯ Important
- ◯ Do not know/Depends
- ◯ Unimportant
- ◯ Should not be included

If a **peer** informs **staff** that another **student** is self-harming in that moment, the **designated team** must; *meet with the **peer** and ensure the **peer** is provided with support.*

- ◯ Essential
- ◯ Important
- ◯ Do not know/Depends
- ◯ Unimportant
- ◯ Should not be included

If a **peer** informs **staff** that another **student** is self-harming in that moment, the **staff member** must; *send the **student** to find a **designated team member** or another **staff member.***

- ◯ Essential
- ◯ Important
- ◯ Do not know/Depends
- ◯ Unimportant
- ◯ Should not be included

If a **peer** informs **staff** that another **student** is self-harming in that moment, the **staff member** must; *go to where the **student** is located immediately.*

- ◯ Essential
- ◯ Important
- ◯ Do not know/Depends
- ◯ Unimportant
- ◯ Should not be included

Responding to events that occur discretely

The following statements relate to **staff** responses in the case that the self-harm is being done **'discretely'** in that present moment**.** This refers to situations where the a **student** is self-harming in that moment, but in a way or in a location where other **students** and **staff** cannot see or not aware of (i.e., not publically).

Please rate the following statements:

The **staff member** who identified the **student**, must discretely intervene by: *removing the means used by the **student** to self-harm.*

- ◯ Essential
- ◯ Important
- ◯ Do not know/Depends
- ◯ Unimportant
- ◯ Should not be included

The **staff member** who identified the **student**, must discretely intervene by: *quietly acknowledging the **student's** distress.*

- ◯ Essential
- ◯ Important
- ◯ Do not know/Depends
- ◯ Unimportant
- ◯ Should not be included

The **staff member** who identified the **student**, must discretely intervene by; *directing the* ***student*** *to come with them to see the* ***medical/first aid staff member*** *if medical attention is required.*

- ◯ Essential
- ◯ Important
- ◯ Do not know/Depends
- ◯ Unimportant
- ◯ Should not be included

First aid and medical staff must; *address the physical needs of any* ***student*** *who presents with a self-harm injury (e.g., clean and dress any wounds, etc).*

- ◯ Essential
- ◯ Important
- ◯ Do not know/Depends
- ◯ Unimportant
- ◯ Should not be included

First aid and medical staff must: *assess the severity and the degree of medical intervention needed (e.g., hospital versus simply cleaning and dressing the wound).*

- ◯ Essential
- ◯ Important
- ◯ Do not know/Depends
- ◯ Unimportant
- ◯ Should not be included

The **staff member** who identified the **student**, must discretely intervene by: *directing the* ***student*** *to come with them to see the* ***designated team****, once medical intervention has been sought.*

- ◯ Essential
- ◯ Important
- ◯ Do not know/Depends
- ◯ Unimportant
- ◯ Should not be included

Responding to events that occur in publicly

The following statements related to **staff** responses in the case that the self-harm is taking place in a visible/public way.

Please rate the following statements:

The staff member who identified the **student**, must de-escalate the situation by: *remaining at a safe distance.*

- Essential
- Important
- Do not know/Depends
- Unimportant
- Should not be included

The **staff member** who identified the **student**, must de-escalate the situation by: *using a gentle tone.*

- Essential
- Important
- Do not know/Depends
- Unimportant
- Should not be included

The **staff member** who identified the **student**, must de-escalate the situation by: *acknowledging the **student's** distress.*

- Essential
- Important
- Do not know/Depends
- Unimportant
- Should not be included

The **staff member** who identified the **student**, must de-escalate the situation by; *trying to remove the means used by the **student** to self-harm**.***

- Essential

- Important
- Do not know/Depends
- Unimportant
- Should not be included

The **staff member** who identified the **student**, must de-escalate the situation by: *expressing concern for the **students** well-being.*

- Essential
- Important
- Do not know/Depends
- Unimportant
- Should not be included

The **staff member** who identified the **student**, must de-escalate the situation by; *giving gentle clear instructions to take some deep breaths.*

- Essential
- Important
- Do not know/Depends
- Unimportant
- Should not be included

The **staff member** who identified the **student**, must de-escalate the situation by; *reassuring the **student** of the desire to provide support ('I am here to support'; 'what can I do to help').*

- Essential
- Important
- Do not know/Depends
- Unimportant
- Should not be included

Once the **student** is calmed down, the **staff member** who identified the **student** must: direct *the **student** to come with them to see the **medical/first aid staff member** if medical attention required or **the designated team***

- Essential
- Important

- ◯ Do not know/Depends
- ◯ Unimportant
- ◯ Should not be included

First aid and medical staff must: *address the physical needs of any **student** who presents with self-harm injuries (e.g., clean and dress any wounds, etc.).*

- ◯ Essential
- ◯ Important
- ◯ Do not know/Depends
- ◯ Unimportant
- ◯ Should not be included

First aid and medical staff must: *assess the severity and the degree of medical intervention needed (e.g., hospital versus simply cleaning and dressing the wound).*

- ◯ Essential
- ◯ Important
- ◯ Do not know/Depends
- ◯ Unimportant
- ◯ Should not be included

The **staff member** must; *send **other students** away (e.g. send to usual assembly point).*

- ◯ Essential
- ◯ Important
- ◯ Do not know/Depends
- ◯ Unimportant
- ◯ Should not be included

If de-escalation of the situation fails, or if the **staff member** needs support, the **staff member** must; *ask a responsible **student** to get assistance from the most appropriate closest **school staff member** (whose job it will be to alert the **designated team**).*

- ◯ Essential
- ◯ Important
- ◯ Do not know/Depends
- ◯ Unimportant

◯ Should not be included

The **designated team** must acknowledge and provide the opportunity for the **students** affected by the incident to get support if it is needed.

◯ Essential
◯ Important
◯ Do not know/Depends
◯ Unimportant
◯ Should not be included

Please add any suggestions for changes to the statements above, suggestions for new statements, or any other comments or feedback you have.

three quarters of the way

${m://FirstName}, thank you for the time you have taken so far.
You are **three quarters** of the way through this questionnaire!

Remember, you can **take a break** after completing each section, and return later to complete the remainder of the questionnaire.

To return to the questionnaire, use the **same link** sent to you in the original email.
Please note that the questionnaire will **expire 6 weeks** after it was sent out.

Click "Next" to continue.

Designated team Response

Designated Team Response

The following items relate to the way in which the **designated team** should respond following the identification of a **student** who self-harms. The **designated person** may have identified the **student** themselves or may have received a referral or crisis call from another **school staff member.**

<u>Please rate the following statements:</u>

The **staff member** to whom the **student** initially presented, may have a better relationship with the **student**, and as such they can offer to facilitate the connection between the **student** and the **designated team member**; <u>*by offering to sit in for part of the meeting.*</u>

- ○ Essential
- ○ Important
- ○ Do not know/Depends
- ○ Unimportant
- ○ Should not be included

All school staff must inform the **designated team** using the schools preferred means of referral and communication (e.g., referral form, email, etc) once immediate needs of the **student** are addressed (e.g., medical needs).

- ○ Essential
- ○ Important
- ○ Do not know/Depends
- ○ Unimportant
- ○ Should not be included

The **relevant designated team member** must meet with the **student** and ensure that the **student** receives the required medical intervention if they have not already.

- ○ Essential
- ○ Important
- ○ Do not know/Depends
- ○ Unimportant
- ○ Should not be included

The **relevant designated team member** must meet with the **student** and:

- Must spend time getting to know the students and their whānau, and listening to the student in order to build rapport
- Outline what confidentiality entails and its limits
- Validate the experiences of the **student** and generate hope for the future
- Discuss any concerns the **student** may have at that moment
- Must obtain the **student's** address, **student's** contact number and the contact details of their **whānau** if not already known

◯ Essential
◯ Important
◯ Do not know/Depends
◯ Unimportant
◯ Should not be included

Once the **designated team member** feels they have built rapport with the **student** they must: *carry out a psychosocial assessment.*

◯ Essential
◯ Important
◯ Do not know/Depends
◯ Unimportant
◯ Should not be included

Once the **designated team member** feels they have built rapport with the **student** they must: *determine the **student's** motivation and intent to die.*

◯ Essential
◯ Important
◯ Do not know/Depends
◯ Unimportant
◯ Should not be included

In order to understand the **student's** self-harm and how to support the **student**, the **designated team member** must explore: *the nature of the **student's** self-harming behaviour (most recent, when and how).*

◯ Essential
◯ Important
◯ Do not know/Depends
◯ Unimportant
◯ Should not be included

In order to understand the **student's** self-harm and how to support the **student**, the **designated team member** must explore: *the function of the **student's** self-harming behaviour (why).*

- Essential
- Important
- Do not know/Depends
- Unimportant
- Should not be included

In order to understand the **student's** self-harm and how to support the **student**, the **designated team member** must explore: *the coping strategies (both adaptive and maladaptive) the **student** is already using to manage distress (including internal and external coping strategies).*

- Essential
- Important
- Do not know/Depends
- Unimportant
- Should not be included

The **designated team member** must determine what supports a **student** has access to (e.g. whānau, friends, school staff, school, church, sports coach, etc) and assist the **student** with increasing their support network (broader than the **designated team member/student** relationship).

- Essential
- Important
- Do not know/Depends
- Unimportant
- Should not be included

The **designated team member** must remind the **student** of the limits of confidentiality, and ask the **student** who they think should be informed.

- Essential
- Important
- Do not know/Depends
- Unimportant
- Should not be included

The **designated team member**, must <u>not</u> suggest that the **student** should stop self-harming, but rather <u>must</u> help the **student** identify other coping strategies and ways of enhancing wellbeing (e.g., time with family, exercise, mindfulness, etc.).

- ◯ Essential
- ◯ Important
- ◯ Do not know/Depends
- ◯ Unimportant
- ◯ Should not be included

The **designated team member** must support the **student** to develop a safety plan by identifying <u>where</u> the **student** can go when distressed, <u>what</u> they can do if they are distressed that will keep them safe, and <u>who</u> they can contact to ask for support (including 24/7 helplines).

- ◯ Essential
- ◯ Important
- ◯ Do not know/Depends
- ◯ Unimportant
- ◯ Should not be included

The **designated team member** must create a no-self-harm contract with the **student**, where the **student** promises not to self-harm until the next time they see the **designated team member**.

- ◯ Essential
- ◯ Important
- ◯ Do not know/Depends
- ◯ Unimportant
- ◯ Should not be included

Please add any suggestions for changes to the statements above, suggestions for new statements, or any other comments or feedback you have.

Contacting whānau

Contacting whānau

The following statements relate to the actions that need to be taken when contacting the **whānau** of a **student** who self-harms or self-harmed.

Please rate the following statements:

The **designated team member** must always ensure that the **student's whānau** are informed of the **student's** self-harm.

- ◯ Essential
- ◯ Important
- ◯ Do not know/Depends
- ◯ Unimportant
- ◯ Should not be included

The **designated team member** must first encourage the **student** to inform their **whānau** of the **student's** self-harm.

- ◯ Essential
- ◯ Important
- ◯ Do not know/Depends
- ◯ Unimportant
- ◯ Should not be included

The **designated team member** must inform the **student** that it is school protocol for the **designated team member** to inform the **student's whānau** of the **student's** self-harm, even if the **student** does not consent.

- ◯ Essential
- ◯ Important
- ◯ Do not know/Depends
- ◯ Unimportant
- ◯ Should not be included

If a **student** discloses abuse, or express concern for their own safety at home, the **designated team member:**

- *must follow the school's child protection policies and procedures (e.g., report of concern),*
- *must consult with relevant services (e.g., Oranga Tamariki), and*
- *must document all consultations and decisions*

◯ Essential
◯ Important
◯ Do not know/Depends
◯ Unimportant
◯ Should not be included

If a **student** discloses abuse, or express concern for their own safety at home, the **designated team member** and the **student** need to identify an alternate **safe adult** that can be informed. Preferably an adult within the **whānau** who can support the **student** outside of school hours.

◯ Essential
◯ Important
◯ Do not know/Depends
◯ Unimportant
◯ Should not be included

The **designated team member** (or most **appropriate person**, e.g., cultural advisor, youth worker, Chaplin, etc) must call the **student's whānau** following a disclosure of self-harm (current or historical).

◯ Essential
◯ Important
◯ Do not know/Depends
◯ Unimportant
◯ Should not be included

The **designated team member** (or most **appropriate person**, e.g., cultural advisor , youth worker, Chaplin, etc) must arrange for a face to face meeting if the **whānau** is interested.

◯ Essential
◯ Important
◯ Do not know/Depends
◯ Unimportant

◯ Should not be included

The **designated team member** (or most **appropriate person**, e.g., cultural advisor, youth worker, Chaplin, etc) must document the phone call.

◯ Essential
◯ Important
◯ Do not know/Depends
◯ Unimportant
◯ Should not be included

The **designated team member** must inform the **whānau** of the school’s self-harm policy.

◯ Essential
◯ Important
◯ Do not know/Depends
◯ Unimportant
◯ Should not be included

The **designated team member** must inform the **whānau** of the **student’s** self-harm.

◯ Essential
◯ Important
◯ Do not know/Depends
◯ Unimportant
◯ Should not be included

The **designated team member** must inform the **whānau** of the safety plan.

◯ Essential
◯ Important
◯ Do not know/Depends
◯ Unimportant
◯ Should not be included

The **designated team member** must inform the **whānau** of available support and resources for the **student.**

- ◯ Essential
- ◯ Important
- ◯ Do not know/Depends
- ◯ Unimportant
- ◯ Should not be included

The **designated team member** must inform the **whānau** of available support and resources for the **whānau** (including the helpline numbers).

- ◯ Essential
- ◯ Important
- ◯ Do not know/Depends
- ◯ Unimportant
- ◯ Should not be included

The **designated team member** <u>does not need to</u> inform the **whānau** of anything other than that the **student** is self-harming (i.e. do not disclose reason for self-harm), unless supported and requested by the **student**.

- ◯ Essential
- ◯ Important
- ◯ Do not know/Depends
- ◯ Unimportant
- ◯ Should not be included

The **designated team member** must inform the **student's whānau** of options for referral.

- ◯ Essential
- ◯ Important
- ◯ Do not know/Depends
- ◯ Unimportant
- ◯ Should not be included

The **designated team member** must involve the **student's whānau** in the decision about whether and where to refer the **student.**

- Essential
- Important
- Do not know/Depends
- Unimportant
- Should not be included

If the **student's whānau** are open to a face-to-face meeting, then the **designated team member** or most appropriate **staff member** must facilitate the meeting, and must include at minimum:

- The **student**
- The **whānau**
- The **designated person**
- Any **support people** (for the student and whānau)
- **Cultural advisor**

- Essential
- Important
- Do not know/Depends
- Unimportant
- Should not be included

The **designated team member** must take time to establish a relationship with the **whānau** of the **student**.

- Essential
- Important
- Do not know/Depends
- Unimportant
- Should not be included

The **designated team member** must schedule a follow-up meeting with **whānau** and **student** after the initial meeting. This typically occurs 1-2 weeks and no later than 1 month after the school detects a self-harm incident.

- Essential
- Important
- Do not know/Depends
- Unimportant
- Should not be included

Please add any suggestions for changes to the statements above, suggestions for new statements, or any other comments or feedback you have.

Referrals

Referrals

The following statements relate to referring a **student** who self-harms to internal school support and external community services.

Please rate the following statements:

The **designated team member** must refer the **student** to support services within the schools. Depending on the school structure and the number of **support staff** available this might mean: *continued support from the **designated person** (if within scope of practice, and if trained) or referral to the **pastoral care team.***

- ◯ Essential
- ◯ Important
- ◯ Do not know/Depends
- ◯ Unimportant
- ◯ Should not be included

If a **student** is experiencing suicidal thoughts with intent to die: *the **designated team member** must consult with their local child and adolescent mental health service to determine the next steps (e.g., appropriateness of referral, etc) and document the consultation (e.g., via phone) and outcome.*

- ◯ Essential
- ◯ Important
- ◯ Do not know/Depends
- ◯ Unimportant
- ◯ Should not be included

If a **student** is experiencing suicidal thoughts with intent to die: *the **designated team member** must follow the guidelines and recommendations in the 'Ministry of Education's preventing and responding to suicide: resource kit for schools'.*

- ◯ Essential
- ◯ Important
- ◯ Do not know/Depends
- ◯ Unimportant
- ◯ Should not be included

The **designated team member** must refer ***students*** to external services if the ***student****; presents with chronic risk.*

- ◯ Essential
- ◯ Important
- ◯ Do not know/Depends
- ◯ Unimportant
- ◯ Should not be included

The **designated team member** must refer **students** to external services if the **student;** *has suicidal thoughts and intent to die.*

- ◯ Essential
- ◯ Important
- ◯ Do not know/Depends
- ◯ Unimportant
- ◯ Should not be included

The **designated team member** must refer **students** to external services if the **student;** *presents with co-morbid difficulties.*

- ◯ Essential
- ◯ Important
- ◯ Do not know/Depends
- ◯ Unimportant
- ◯ Should not be included

The **designated team member** must refer **students** to external services if the **student;** *has limited protective factors.*

- Essential
- Important
- Do not know/Depends
- Unimportant
- Should not be included

The **designated team member** must refer **students** to external services if the **student;** *has multiple risk factors.*

- Essential
- Important
- Do not know/Depends
- Unimportant
- Should not be included

The **designated team member** must refer **students** to external services if the **student**; *has a complex presentation and complex environmental factors.*

- Essential
- Important
- Do not know/Depends
- Unimportant
- Should not be included

The **designated team member** must refer **students** to external services if; the ***designated team*** *believes it is outside of the scope of practice of the* ***designated team*** *or other s****chool pastoral care staff.***

- Essential
- Important
- Do not know/Depends
- Unimportant
- Should not be included

Along with making a referral to an external agency, the **designated team member** must make a phone call or send an email to the external agency to discuss; *the*

appropriateness of the referral.

- ◯ Essential
- ◯ Important
- ◯ Do not know/Depends
- ◯ Unimportant
- ◯ Should not be included

Along with making a referral to an external agency, the **designated team member** must make a phone call or send an email to the external agency to discuss; *any missing information/further assessment required.*

- ◯ Essential
- ◯ Important
- ◯ Do not know/Depends
- ◯ Unimportant
- ◯ Should not be included

Along with making a referral to an external agency, the **designated team member** must make a phone call or send an email to the external agency *to discuss; wait-time/holding pattern.*

- ◯ Essential
- ◯ Important
- ◯ Do not know/Depends
- ◯ Unimportant
- ◯ Should not be included

If an external referral is required, the **designated team member** must make a referral to an external service that is relevant, appropriate and accessible to the **student** and their **whānau**. This will require consideration of;

- Location of the service
- Referral criteria of the service
- Specialisation of the service
- Financial requirements of the service
- Places that the student is already connected to
- Cultural appropriateness of the service

- ◯ Essential
- ◯ Important
- ◯ Do not know/Depends
- ◯ Unimportant

○ Should not be included

Please add any suggestions for changes to the statements above, suggestions for new statements, or any other comments or feedback you have.

Ongoing support

Ongoing support

The following statements relate to the ongoing support required by **students** and their **whānau**, after the initial response to identifying a **student** who self-harms.

Please rate the following statements:

Following the initial response, the **designated team member** must act as a liaison between:

- the **student**
- **whānau**
- affected **school staff**
- **peers**
- outside referral agents associated with the **student** as a result of the disclosure

○ Essential
○ Important
○ Do not know/Depends
○ Unimportant
○ Should not be included

If a referral is made to external agencies the **designated team member** must follow-up with the **student**, their **whānau** and the external agency to: *identify how the **designated team member** can support the **student** and **whānau** while the **student** is at school.*

- Essential
- Important
- Do not know/Depends
- Unimportant
- Should not be included

If a referral is made to external agencies the **designated team member** must follow-up with the **student**, their **whānau** and the external agency to: *identify how **school staff** can support the **student** and their **whānau** while at school.*

- Essential
- Important
- Do not know/Depends
- Unimportant
- Should not be included

The **designated team member** must ensure that all relevant **staff** know what they need to do in order to support the **student**, which may involve:

- Monitoring
- In class support
- Encouraging involvement in school extracurricular activities

- Essential
- Important
- Do not know/Depends
- Unimportant
- Should not be included

The **designated team member** must ensure that any related issues and concerns (e.g., substance use, etc.) are addressed, if within their scope of practice and if an external referral was not made, using evidence-based approaches.

- Essential
- Important
- Do not know/Depends
- Unimportant
- Should not be included

The **designated team**, must engage in evidence-based interventions and therapies to support the **student**.

- Essential
- Important
- Do not know/Depends
- Unimportant
- Should not be included

The **designated team member** must continue to support **students** by: *engaging in psychoeducation on self-harm and related issues.*

- Essential
- Important
- Do not know/Depends
- Unimportant
- Should not be included

The **designated team member** must continue to support students by: *identifying triggers (including social media).*

- Essential
- Important
- Do not know/Depends
- Unimportant
- Should not be included

The **designated team member** must continue to support **students** by: *exploring the **students** interests and skills, and tailoring other strategies with this in mind.*

- Essential
- Important
- Do not know/Depends
- Unimportant
- Should not be included

The **designated team member** must continue to support **students** by: *identifying alternative coping and relaxation strategies.*

- Essential
- Important
- Do not know/Depends
- Unimportant
- Should not be included

The **designated team member** must continue to support **students** by: *identifying prevention.*

- Essential
- Important
- Do not know/Depends
- Unimportant
- Should not be included

The **designated team member** must continue to support **students** by: *understanding their emotions, thoughts, behaviours, values and strengths.*

- Essential
- Important
- Do not know/Depends
- Unimportant
- Should not be included

The **designated team member** must continue to support **students** by: *continuing to review and update the safety plan with the **student** and their **whānau.***

- Essential
- Important
- Do not know/Depends
- Unimportant
- Should not be included

The **designated team member** must continue to support **students** by: *exploring ways of enhancing all aspects of the **student's** wellbeing (i.e. physical, spiritual, emotional, social, and environmental).*

- Essential
- Important

- ◯ Do not know/Depends
- ◯ Unimportant
- ◯ Should not be included

The **designated team member** must continue to support the **student** to develop and enhance their support network by: *exploring existing supports.*

- ◯ Essential
- ◯ Important
- ◯ Do not know/Depends
- ◯ Unimportant
- ◯ Should not be included

The **designated team member** must continue to support the **student** to develop and enhance their support network by: *exploring options of new activities to engage in that might offer social support and that is in line with the **student's** interests, beliefs and values.*

- ◯ Essential
- ◯ Important
- ◯ Do not know/Depends
- ◯ Unimportant
- ◯ Should not be included

The **designated team member** must ensure the **student** has skills, strategies and resources that they can use when they are not in school (e.g., apps, phone numbers, relaxation skills, safety plan, etc).

- ◯ Essential
- ◯ Important
- ◯ Do not know/Depends
- ◯ Unimportant
- ◯ Should not be included

The **designated team member** must contact the **student's whānau** regularly, and need to provide support as needed (e.g., referral for whānau, psychoeducation, validate, meeting, etc.).

- ◯ Essential

○ Important
○ Do not know/Depends
○ Unimportant
○ Should not be included

If needed and requested by the **whānau**, the **designated team member and the appropriate staff member** (e.g., kaumatua) must support **whānau** with adapting parenting approaches (e.g. developing empathic understandings) to support their distressed child. This could be offered in a group format and must consider the cultural context of parenting practices.

○ Essential
○ Important
○ Do not know/Depends
○ Unimportant
○ Should not be included

The **designated team member** and the appropriate **staff member** must support **whānau** by providing the **student's whānau** with relevant resources (e.g., helplines, written information about parenting and creating a safe home environment, etc.).

○ Essential
○ Important
○ Do not know/Depends
○ Unimportant
○ Should not be included

Please add any suggestions for changes to the statements above, suggestions for new statements, or any other comments or feedback you have.

Social Contagion

Social Contagion

The following statements relate to actions **school staff** can take in order to prevent or respond to social contagion of self-harm.

Key term(s) introduced in this section:
Social contagion: In this questionnaire social contagion refers to the social contagion of self-harm and other suicidal behaviours. Social contagion of self-harm is defined in this questionnaire as, the spread of self-harming behaviour following exposure to self-harming behaviour or suicide by others. This exposure can occur directly (e.g., family, peer groups or community) or indirectly (e.g., media).

<u>Please rate the following statements:</u>

All school staff must support and endorse self-harm prevention efforts within the school, as a means of preventing social contagion (e.g., psychoeducation, mindfulness in class, etc.).

- ◯ Essential
- ◯ Important
- ◯ Do not know/Depends
- ◯ Unimportant
- ◯ Should not be included

The **designated team member** must bring groups of **students** who self-harm together to provide psychoeducation and interventions.

- ◯ Essential
- ◯ Important
- ◯ Do not know/Depends
- ◯ Unimportant
- ◯ Should not be included

The **school leadership team and the principal** must meet with all **students** who self-harm in response to social contagion, and inform them that they will be sent home if they self-harm in school.

- ◯ Essential
- ◯ Important
- ◯ Do not know/Depends
- ◯ Unimportant
- ◯ Should not be included

All school staff must ensure that **students** cover any signs of self-harm (e.g., scars) with clothing, bandages, plasters, or tape.

- ◯ Essential
- ◯ Important
- ◯ Do not know/Depends
- ◯ Unimportant
- ◯ Should not be included

First aid and medical staff must ensure that any tape, bandages or plasters used to cover self-harm injuries are not identifiable or stand out, therefore preventing it from becoming a symbol of self-harm.

- ◯ Essential
- ◯ Important
- ◯ Do not know/Depends
- ◯ Unimportant
- ◯ Should not be included

Please add any suggestions for changes to the statements above, suggestions for new statements, or any other comments or feedback you have.

Resources

Resources

The following statements relate to the resources needed in schools in order for **staff** to support **students** who self-harm.

<u>Please rate the following statements:</u>

All school staff must have access to any copies of relevant Ministry of Education guidelines and toolkits.

- Essential
- Important
- Do not know/Depends
- Unimportant
- Should not be included

The **designated team** must have access to pamphlets, booklets, tools and other resources that can be used to educate **students**, **whānau** and **staff** about self-harm and related issues.

- Essential
- Important
- Do not know/Depends
- Unimportant
- Should not be included

The **designated team** must have access to pamphlets, booklets, tools and other resources that can be used to provide **students** with ideas and information on alternate coping strategies (e.g., mindfulness, distraction techniques, etc.).

- Essential
- Important
- Do not know/Depends
- Unimportant
- Should not be included

The **board of trustees** must adhere to the NZAC guideline for the **counsellor** to student ratio (1:400), and therefore should ensure that enough **counsellors** are in the school to support **students**.

- Essential
- Important
- Do not know/Depends
- Unimportant
- Should not be included

The **board of trustees** and the **senior leadership team** must work in consultation with the **designated team, pastoral care staff** and **medical staff** to ensure that the

required resourcing is available to complete self-harm screening within the school (e.g., staff, time, tools, money, and physical space).

- Essential
- Important
- Do not know/Depends
- Unimportant
- Should not be included

In a rural school, the **board of trustees** must hire a **pastoral care professional (e.g., counsellor**) who has the necessary skills to identify and manage self-harm.

- Essential
- Important
- Do not know/Depends
- Unimportant
- Should not be included

If a **staff member** is balancing multiple roles, where one of the roles is that of a **school counsellor or designated team member**, the **senior leadership team** must ensure that the **staff member** is provided with support to allocate a set amount of time dedicated to managing the care of **students** who self-harm.

- Essential
- Important
- Do not know/Depends
- Unimportant
- Should not be included

Please add any suggestions for changes to the statements above, suggestions for new statements, or any other comments or feedback you have.
