# Supplementary material for: Development of guidelines for school staff on supporting students who self-harm: a Delphi study
Source: BMC Psychiatry. 2022 Sep 29;22:631. doi: 10.1186/s12888-022-04266-7 (PMC9520113; doi:10.1186/s12888-022-04266-7)
Supplement: Supplementary file 2 — Additional file 2. [file 12888_2022_4266_MOESM2_ESM.pdf]

Questionnaire Information

Questionnaire Information

Thank you for taking the time to complete the second and final questionnaire. Using the results of the first questionnaire, of the 308 statements, 184 statements were endorsed by our panel members, 20 were rejected, and 104 statements did not reach consensus. Furthermore 25 new statements were suggested by panel members.

As a result the 104 statements that did not reach consensus are included in this questionnaire to be re-rated, along with the 25 new statements.We have included a table below statements that need to be re-rated, which shows the percentage of panel members who rated the items as important or essential, and the percentage of panel members who rated the items as unimportant or should not be included. Based on the feedback from panel members, we have also included some additional information for items where context and clarification was required or suggested. Similarly, for some statements we have made changes to the wording of statements as suggested.

At the end of each section, there is an open text box if you have any comments and thoughts about that section or particular statements.

Can I save and return to the questionnaire later?

Your progress will be automatically saved when you click “**Next**” at the end of each section. This means you can complete one or several sections at a time, save it, and come back to it rather than completing it in one session. To return to the questionnaire, simply click on the original link you were emailed. Please note that this **link will expire after 6 weeks**, which means you have only 6 weeks to complete the survey.

What do I need to do in this questionnaire?

Please rate each statement based on whether you believe the statement should be in the guideline. Thus rate responses based on whether you believe it is appropriate and helpful for school staff, students, and their whānau. ***Please note, that the scale has changed*** from the scale in the first questionnaire. Based on the feedback and responses we received we have separated out “Don’t know/Depends”. The new rating scale is:

- Essential
- Important
- Depends
- Unimportant
- Should not be included
- Don’t know

You will also have the option on providing us with more information if you select "depends".

Key terms used throughout the questionnaire:

All School staff: Any and all adults employed at the school

Self-harm: Intentional self-injury (including poisoning) that occurs regardless of motivation or degree of suicidal intent

Click “**next**”, if you are ready to begin.

School Culture and Environment

School Culture and Environment

The following statements relate to the ways in which the school environment and culture influences **student** wellbeing, how **all school staff** can engage in actions that support the wellbeing of **students**, and ways for creating a supportive school environment.
Key terms introduced in this section:
Senior leadership team: School staff who hold leadership positions within the school, this includes but is not limited to heads of departments, Deans, school principal, deputy principals, Special education needs coordinator (SENCO).

Please rate the following **new** statements:

The pastoral care team must ensure that the **pastoral care** provided to **students** is culturally sensitive, safe and responsive to the cultural needs of the **student** and their **whānau**.
i.e., culturally sensitive and responsive to the needs and worldviews of students and whānau who identify as Māori, Pasifika, Asian, diverse (e.g., LGBTQI++, refugee, etc.).

- Essential
- Important
- Depends (if applicable, please provide more information in the text box below)

- Unimportant
- Should not be included
- Don't Know

All school staff should have training to increase either mental health literacy (e.g., MH101, mental health first aid, etc)

- Essential
- Important
- Depends (if applicable, please provide more information in the text box below)

- Unimportant
- Should not be included
- Don't Know

The **senior leadership team** should ensure that staff are supported to engage in self-care (e.g, setting up a peer support system).

- Essential
- Important
- Depends (if applicable, please provide more information in the text box below)

○ Unimportant
○ Should not be included
○ Don't Know

Students who miss any school activities (e.g., class, sport sign-up, etc) must be offered opportunities to catch-up by the **relevant staff member**.

○ Essential
○ Important
○ Depends (if applicable, please provide more information in the text box below)
○ Unimportant
○ Should not be included
○ Don't Know

Please leave any thoughts or comments about this section below:

The Role of School Staff in Supporting Students who Self-Harm

The Role of School Staff in Supporting Students who Self-Harm

The following statements relate to the various roles and responsibilities of **schools staff** in supporting **students** who self-harm.

Please re-rate the following statements:

In acknowledgement of the fact that school can be a protective factor for students, and the potential risks associated with being alone at home for students who self-harm; the **senior leadership team** (and **other staff** involved) must not exclude/stand-down **students** because they self-harm.

Note: This items is different to the situation where the student and school staff decide together, that the student wants or needs time away from school. This item is in the context of maintaining a student at school with considerable support for them and other students. It seeks to ensure that students do not feel punished for their distress (that manifests as self-harm).

	**Essential + Important**	**Unimportant + not include**
Professionals Panel	**71.9%**	**6.2%**

Rangatahi Panel	**81.5%**	**0.0%**

- Essential
- Important
- Depends (if applicable, please provide more information in the text box below)
- Unimportant
- Should not be included
- Don't Know

Please rate the following **new** statements:

Staff members who are supporting a **student** who is engaging in self-harm, must be facilitated to engage with an appropriate support person (e.g., principal, pastoral care staff member, Team leader)

- Essential
- Important
- Depends (if applicable, please provide more information in the text box below)
- Unimportant
- Should not be included
- Don't Know

School students should be involved in the development and review of school guidelines and policies.

- Essential
- Important
- Depends (if applicable, please provide more information in the text box below)
- Unimportant
- Should not be included
- Don't Know

Please leave any thoughts or comments about this section below:

Designated Team

Designated Team Responsible for Supporting Students who Self-Harm

All school staff are responsible for the wellbeing of **students**, however, supporting **students** who self-harm involves specific responsibilities, required knowledge and skills.

From here onward the team tasked with the responsibility of supporting **students** who *have self-harmed, are self-harming, or may self-harm in the future (prevention)* will be referred to as the '**designated team**'. This team will have specific responsibilities, and will need to be trained to provide them with the skills and knowledge needed to support distressed **students** and their **whānau**.
We acknowledge that some schools may not be able to have a **team** fully trained and dedicated to supporting **students** who self-harm. It is recommended, that even if only **one staff member** is able to formally be trained, a **group of school staff** should still form part of the **designated team**.

Clarification:
The designated team should include pastoral care and wellbeing staff (e.g. Counsellor, social worker, youth worker, nurse, etc.) if available to the school. In schools where there are limited resources, the designated team should include at least one staff member who is willing to attend the training, and at least one member of the senior leadership team to support them in their role.
The following statements relate to the steps that need to be taken in order to **establish who** the **designated team** will be.
Please re-rate the following statements:

The **school leadership team** must choose which **staff members** will be part of the **designated team.**

	**Essential + Important**	**unimportant + not include**
Professionals Panel	**78.1%**	**3.1%**
Rangatahi Panel	**59.3%**	**7.4%**

- Essential
- Important
- Depends (if applicable, please provide more information in the text box below)
- Unimportant
- Should not be included
- Don't Know

The **school leadership team** must allow any **staff member** to volunteer to be part of the **designated team** based on the **staff member's** interest and suitability for the role.

Note: Things to consider around suitability is if the person is genuine, non-judgemental, good at establishing relationships with students, and obtained the training required. When considering suitability it is also important to include young people's perspectives.

	**Essential + Important**	**unimportant + not include**
Professionals Panel	**65.6%**	**12.5%**
Rangatahi Panel	**85.2%**	**7.4%**

- Essential
- Important
- Depends (if applicable, please provide more information in the text box below)
- Unimportant
- Should not be included
- Don't Know

Please rate the following **new** statements:

All school websites should have a tab that clearly identifies who is in the **designated team** (ideally with a photo a bio and photo).

- Essential
- Important
- Depends (if applicable, please provide more information in the text box below)
- Unimportant
- Should not be included
- Don't Know

Please leave any thoughts or comments about this section below:

Policies

Policies and Procedures relating to Self-Harm in Schools

The following statements relate to policies and procedures within schools that relate to self-harm, and how schools can support **students** who self-harm through policies and procedures.

Please rate the following statements:

The **designated team** must lead the development of a written procedure outlining: *How the* ***student's whānau*** *will be informed and involved following a disclosure from or identification of a* ***student*** *who self-harms.*

Note: following an assessment of safety, and if the self-harm is current. Whānau should be considered in its broadest sense, and include any safe adult who can provide support (e.g., older cousin, youth pastor, older siblings, etc.).

	Essential + Important	Unimportant + not include
Professionals Panel	**86.7%**	**3.3%**
Rangatahi Panel	**76.0%**	**12.0%**

- Essential
- Important
- Depends (if applicable, please provide more information in the text box below)

- Unimportant
- Should not be included
- Don't Know

Teachers (and in some cases **students**) can choose their own teaching materials and resources (e.g. media, literature) that can be inadvertently triggering and distressing to **students**.

Because of this, the board of trustees and the school principal must develop and implement a school policy and procedure that outlines expectations around choosing teaching material and resources.

Note: this is not saying the materials should not be used, but there should be guidance around these materials (e.g., policy could outline an expectations that trigger warnings should be used)

	Essential + Important	unimportant + not include
Professionals Panel	**63.3%**	**20.0%**
Rangatahi Panel	**72.0%**	**8.0%**

- Essential
- Important
- Depends (if applicable, please provide more information in the text box below)

- Unimportant
- Should not be included
- Don't Know

<u>Please rate the following **new** statements:</u>

Following a suicide attempts that takes place at school, there should be a review to ensure that the policy and procedure is followed.

- Essential
- Important
- Depends (if applicable, please provide more information in the text box below)
- Unimportant
- Should not be included
- Don't Know

Please leave any thoughts or comments about this section below:

Prevention

Prevention

The following statements relate to initiatives, campaigns, programmes and steps that can be taken within the school to prevent self-harm, and the various ways **school staff** can play a role in prevention.

Key term(s) introduced in this section:
Psychoeducation: The delivery of accurate information about an issue or diagnosis, associated short and long–term outcomes, management strategies, and prevention strategies. This information can be delivered to, or aimed at individuals, their whānau, the community or mental health professionals.

<u>Please re-rate the following statements:</u>

The **senior Leadership team** must ensure **teaching staff** provide **students** with trigger warnings and alternative (non-triggering) choices to teaching materials and resources (e.g. Media, literature) if the **staff member** chooses topics or resources that have triggering themes (e.g., self-harm, suicide, trauma, etc).
Note: provision of alternative materials must be done in a way that ensures the confidentiality and privacy of the student.

	Essential +	unimportant +

	Important	not include
Professionals Panel	**92.6%**	**0.0%**
Rangatahi Panel	**78.3%**	**0.0%**

- Essential
- Important
- Depends (if applicable, please provide more information in the text box below)
- Unimportant
- Should not be included
- Don't Know

The **teaching staff** must *inform* a **designated team member** of their class topic choices that include references to triggering content e.g. self-harm, suicide, etc.
Note: This can be simply sending an email to the designated team informing that of what will be covered in class.

	Essential + Important	unimportant + not include
Professionals Panel	**70.4%**	**11.1%**
Rangatahi Panel	**73.9%**	**8.6%**

- Essential
- Important
- Depends (if applicable, please provide more information in the text box below)
- Unimportant
- Should not be included
- Don't Know

Teachers who choose to use materials that reference triggering content (e.g. self-harm, suicide) must; *inform the **designated team** of what material will be used and when.*

	Essential + Important	unimportant + not include
Professionals Panel	**81.5%**	**3.7%**
Rangatahi Panel	**73.9%**	**8.7%**

- Essential
- Important

○ Depends (if applicable, please provide more information in the text box below)

○ Unimportant

○ Should not be included

○ Don't Know

Teachers who choose to use materials that reference triggering content (e.g. self-harm, suicide) must; *inform the **whānau** of the **students** that the **teacher** has selected material that may be triggering or distressing for **students**.*
Note: depending on the age of the student (e.g., there have been instances where a children's cartoon was shown to primary school children where a character completes suicide)

	Essential + Important	unimportant + not include
Professionals Panel	**74.1%**	**3.7%**
Rangatahi Panel	**39.1%**	**4.3%**

○ Essential

○ Important

○ Depends (if applicable, please provide more information in the text box below)

○ Unimportant

○ Should not be included

○ Don't Know

Teachers who choose to use materials that reference triggering content (e.g. self-harm, suicide) must; *consult with a **designated team member** about how to check-in with **students** before and after the material is used/viewed.*
Note: Here the term check-ins refers to introducing and debriefing with students after the material is used. This can be as simple as informing the class that if anyone is distressed they can talk to the staff member, the designated team or contact a help-line.

	Essential + Important	unimportant + not include
Professionals Panel	**81.5%**	**3.7%**
Rangatahi Panel	**73.9%**	**4.3%**

○ Essential

○ Important

○ Depends (if applicable, please provide more information in the text box below)

○ Unimportant

○ Should not be included

○ Don't Know

Teachers who choose to use materials that reference triggering content (e.g. self-harm, suicide) must; *complete check-ins with all **students** before and after the material is used/viewed.*
Note: Here the term check-ins refers to introducing and debriefing with students after the material is used. This can be as simple as informing the class that if anyone is distressed they can talk to the staff member, the designated team or contact a help-line.

	**Essential + Important**	**unimportant + not include**
Professionals Panel	**70.4%**	**7.4%**
Rangatahi Panel	**65.2%**	**4.3%**

○ Essential

○ Important

○ Depends (if applicable, please provide more information in the text box below)

○ Unimportant

○ Should not be included

○ Don't Know

Teachers who choose to use materials that reference triggering content (e.g. self-harm, suicide) must; *provide help seeking information (e.g., help-line numbers, websites, etc.) to **students**, after **student's** viewed or engaged with triggering material.*

	**Essential + Important**	**unimportant + not include**
Professionals Panel	**96.3%**	**0.0%**
Rangatahi Panel	**73.9%**	**0.0%**

○ Essential

○ Important

○ Depends (if applicable, please provide more information in the text box below)

○ Unimportant

○ Should not be included

○ Don't Know

In the event that a **student** chooses to use materials that reference triggering content (e.g. choosing to write a report on a movie that references self-harm) the **teacher** must; *inform a **designated team member** and discuss how the **teacher** can support the **student**. While considering factors such as the **student's** risk and age (i.e., these factors will influence whether whānau are informed, if check in occurs, etc.).*

Note: this would only occur following a discussion with the student

	Essential + Important	unimportant + not include
Professionals Panel	**81.5%**	**11.1%**
Rangatahi Panel	**60.9%**	**4.3%**

○ Essential

○ Important

○ Depends (if applicable, please provide more information in the text box below)

○ Unimportant

○ Should not be included

○ Don't Know

In the event that a **student** chooses to use materials that reference triggering content (e.g. choosing to write a report on a movie that references self-harm) the **teacher** must; provide **students** with a warning that the content of the self-selected material (book, movie, poem, play, etc.) may be distressing or triggering.
Note: This is in the context where the teacher becomes aware of the content of material the student is using, while also considering the age of the student.

	Essential + Important	unimportant + not include
Professionals Panel	**81.5%**	**7.4%**
Rangatahi Panel	**65.2%**	**13.0%**

○ Essential

○ Important

○ Depends (if applicable, please provide more information in the text box below)

○ Unimportant

○ Should not be included

○ Don't Know

In the event that a **student** chooses to use materials that reference triggering content (e.g. choosing to write a report on a movie that references self-harm) the **teacher** must; *provide **students** with suggestions for different materials, but NOT force the **student** to choose a different option.*

	Essential + Important	unimportant + not include
Professionals Panel	**70.4%**	**14.8%**
Rangatahi Panel	**47.8%**	**17.3%**

○ Essential

○ Important

○ Depends (if applicable, please provide more information in the text box below)

○ Unimportant

○ Should not be included

○ Don't Know

In the event that a **student** chooses to use materials that reference triggering content (e.g. choosing to write a report on a movie that references self-harm) the **teacher** must; *consult with a **designated team member** about how to check-in with the **student** before and after the self-selected material is used/viewed.*

	Essential + Important	unimportant + not include
Professionals Panel	**74.1%**	**14.8%**
Rangatahi Panel	**65.2%**	**8.7%**

○ Essential

○ Important

○ Depends (if applicable, please provide more information in the text box below)

- Unimportant
- Should not be included
- Don't Know

In the event that a **student** chooses to use materials that reference triggering content (e.g. choosing to write a report on a movie that references self-harm) the **teacher** must; *complete check-ins with the **student** before and after the self-selected material is viewed/used.*
Note: this does not need to be a formal process, it is simply checking whether the student is okay and needs any support.

	**Essential + Important**	**unimportant + not include**
Professionals Panel	**74.1%**	**7.4%**
Rangatahi Panel	**65.2%**	**8.7%**

- Essential
- Important
- Depends (if applicable, please provide more information in the text box below)

- Unimportant
- Should not be included
- Don't Know

In the event that a **student** chooses to use materials that reference triggering content (e.g. choosing to write a report on a movie that references self-harm) the **teacher** must; *provide help seeking information (including local help-line numbers, websites, etc.) to the **student**, after the **student** viewed or engaged with potentially triggering self-selected material.*
Note: This would occur if the check-in with the student raised some concerns about the student. The information provided will include that the student can receive support from the designated team if they want it.

	**Essential + Important**	**unimportant + not include**
Professionals Panel	**81.5%**	**3.7%**
Rangatahi Panel	**43.5%**	**13.0%**

- Essential
- Important
- Depends (if applicable, please provide more information in the text box below)

- Unimportant

- Should not be included
- Don't Know

The **designated team** must develop and implement psychoeducation programmes for **students**, their **whānau**, and **staff** that are in line with the cultural values, needs and worldviews of participants.

	Essential + Important	unimportant + not include
Professionals Panel	**77.8%**	**7.4%**
Rangatahi Panel	**69.6%**	**4.3%**

Please rate the re-worded statement below:
Any psychoeducation programme developed and implemented by the **designated team** for **students**, their **whānau**, and **staff** should be in line with the cultural values, needs and worldviews of the students, whānau and staff.

- Essential
- Important
- Depends (if applicable, please provide more information in the text box below)

- Unimportant
- Should not be included
- Don't Know

Psychoeducation programmes should aim to inform **students**, their **whānau** and **staff** about; *the facts about self-harm and emotional distress.*

	Essential + Important	unimportant + not include
Professionals Panel	**74.1%**	**3.7%**
Rangatahi Panel	**87.0%**	**0.0%**

- Essential
- Important
- Depends (if applicable, please provide more information in the text box below)

- Unimportant
- Should not be included
- Don't Know

The **designated team** must introduce and facilitate prevention programmes that focus on enhancing the wellbeing of **students** by; *supporting **students** to connect with their **whānau** and **peers**.*

	Essential + Important	unimportant + not include
Professionals Panel	**85.2%**	**0.0%**
Rangatahi Panel	**78.3%**	**0.0%**

- Essential
- Important
- Depends (if applicable, please provide more information in the text box below)

- Unimportant
- Should not be included
- Don't Know

The **designated team**, must proactively inform **whānau** and **school staff**, about:*the difficulties **rangatahi/young people** face.*
Note: it is important that whānau and school staff are continually educated about the challenges students face.

	Essential + Important	unimportant + not include
Professionals Panel	**74.1%**	**11.1%**
Rangatahi Panel	**87.0%**	**4.3%**

- Essential
- Important
- Depends (if applicable, please provide more information in the text box below)

- Unimportant
- Should not be included
- Don't Know

The designated team, must proactively inform **whānau** and **school staff**, about: *the challenges and benefits of social media.*
Note: this is not about stopping young people from using social media, but making sure that those around them and support them are aware that they may need more support. Additionally it may be about highlighting the benefits of social media, so that whānau and school staff do not have overly negative views of social media.

	Essential +	unimportant +

	Important	not include
Professionals Panel	**81.5%**	**3.7%**
Rangatahi Panel	**65.2%**	**8.7%**

- Essential
- Important
- Depends (if applicable, please provide more information in the text box below)

- Unimportant
- Should not be included
- Don't Know

The **designated team**, must proactively inform **whānau** and **school staff**, about: *the value and importance of* ***whānau*** *connection and acceptance.*

	Essential + Important	unimportant + not include
Professionals Panel	**85.2%**	**0.0%**
Rangatahi Panel	**73.9%**	**4.3%**

- Essential
- Important
- Depends (if applicable, please provide more information in the text box below)

- Unimportant
- Should not be included
- Don't Know

The **designated team** must encourage **students** to form **peer-led groups** that; *advocate for inclusion and acceptance of* ***all students****.*

	Essential + Important	unimportant + not include
Professionals Panel	**74.1%**	**7.4%**
Rangatahi Panel	**73.9%**	**8.7%**

Please rate the re-worded statement below:
The **designated team** *can* encourage **students** to form **peer-led groups** that; *advocate for inclusion and acceptance of* ***all students****.*

- Essential
- Important
- Depends (if applicable, please provide more information in the text box below)
- Unimportant
- Should not be included
- Don't Know

the **designated team** must encourage **students** to form **peer-led groups** that: *aim to enhance awareness of issues* ***students*** *face or are passionate about (e.g., mental health).*

	Essential + Important	unimportant + not include
Professionals Panel	**66.7%**	**7.4%**
Rangatahi Panel	**87.0%**	**4.3%**

Please rate the re-worded statement below:

The **designated team** *can* encourage **students** to form **peer-led groups** that: *aim to enhance awareness of issues* ***students*** *face or are passionate about (e.g., mental health).*

- Essential
- Important
- Depends (if applicable, please provide more information in the text box below)
- Unimportant
- Should not be included
- Don't Know

The **designated team** must advocate for and implement evidence-based screening programmes that aim to identify **students** who self-harm or are at risk of self-harming.
Note: This statement was close to being endorsed. We know there is evidence for the efficacy of screening programmes to prevent suicide because they detect students who otherwise may not have been identified, sought support or who self-harm. Screening has to take place in the context of sufficient resources to do this and effective pathways of care for those identified by screening. This type of screening could be conducted online or with paper and pencil. This recommendation would be made in the context of the importance of including young people in the design of screening programmes (e.g., young people might prefer that the screening occurs online or with paper in pencil, rather than in person with someone they are not as comfortable with). Furthermore developmentally appropriate screening tools would need to be selected.

	Essential + Important	unimportant + not include
Professionals Panel	**77.8%**	**0.0%**
Rangatahi Panel	**69.6%**	**4.3%**

- Essential
- Important
- Depends (if applicable, please provide more information in the text box below)
- Unimportant
- Should not be included
- Don't Know

In recognition of the link between self-harm and a number of other factors, the **designated team** must ask **students** if they have or are self-harming if they present with or disclose; *experiencing bullying.*

Note: this would only occur in the context of an already established (even if relatively newly established) relationship

	Essential + Important	unimportant + not include
Professionals Panel	**81.5%**	**0.0%**
Rangatahi Panel	**73.9%**	**4.3%**

- Essential
- Important
- Depends (if applicable, please provide more information in the text box below)
- Unimportant
- Should not be included
- Don't Know

In recognition of the link between self-harm and a number of other factors, the **designated team** must ask **students** if they have or are self-harming if they present with or disclose; *substance use.*
Note: this would only occur in the context of an already established (even if relatively newly established) relationship

	Essential + Important	unimportant + not include
Professionals Panel	**74.1%**	**0.0%**
Rangatahi Panel	**65.2%**	**8.7%**

- Essential
- Important
- Depends (if applicable, please provide more information in the text box below)
- Unimportant
- Should not be included
- Don't Know

In recognition of the link between self-harm and a number of other factors, the **designated team** must ask **students** if they have or are self-harming if they present with or disclose; *trauma experiences (including any experiences of abuse).*
Note: this would only occur in the context of an already established (even if relatively newly established) relationship

	Essential + Important	unimportant + not include
Professionals Panel	**81.5%**	**0.0%**
Rangatahi Panel	**69.6%**	**4.3%**

- Essential
- Important
- Depends (if applicable, please provide more information in the text box below)
- Unimportant
- Should not be included
- Don't Know

In recognition of the link between self-harm and a number of other factors, the **designated team** must ask **students** if they have or are self-harming if they present with or disclose: *grief and loss.*
Note: grief is a normal response to loss, but for some the loss can be triggering particularly in the context of limited coping mechanism and supports.

	Essential + Important	unimportant + not include

Professionals Panel	**74.1%**	**7.4%**
Rangatahi Panel	**69.6%**	**0.0%**

- Essential
- Important
- Depends (if applicable, please provide more information in the text box below)
- Unimportant
- Should not be included
- Don't Know

Please rate the following new statements:
The **Designated team** must conduct developmentally appropriate wellbeing screens within the school, which can include items on self-harm and distress, with the aim of identifying the needs and strengths of students.

- Essential
- Important
- Depends (if applicable, please provide more information in the text box below)
- Unimportant
- Should not be included
- Don't Know

Please leave any thoughts or comments about this section below:

Professional Development: Training

Professional Development: Training
The following section focuses on the training requirements for the professional development of **school staff**, in relation to supporting **students** who self-harm.
Please rate the following statements:

The **senior leadership team** must only recommend and approve external training programmes and workshops (related to self-harm) for **school staff** that are: *facilitated by*

professionals from Aotearoa who understand the needs, realities and issues in Aotearoa.

Note: this would be important to ensure that the cultural context is taken into account in the training.

	Essential + Important	unimportant + not include
Professionals Panel	**72.0%**	**12.0%**
Rangatahi Panel	**77.3%**	**13.6%**

- Essential
- Important
- Depends (if applicable, please provide more information in the text box below)
- Unimportant
- Should not be included
- Don't Know

The **senior leadership team** must only recommend and approve external training programmes and workshops (related to self-harm) for **school staff** that are: *culturally-relevant.*

Note: To ensure that the worldviews and experiences of students, staff and whānau, are considered in the support and response to self-harm from school staff, training programmes need to also include these worldviews and experiences. Therefore the statement above relates to ensuring that training programmes are responsive and relevant to the worldviews and experiences of Māori, Pasifika, Asian and diverse communities (e.g., LGBTQI++, refugee communities, etc.) and assumes it will be relevant to tamariki and rangatahi.

	Essential + Important	unimportant + not include
Professionals Panel	**88.0%**	**0.0%**
Rangatahi Panel	**63.9%**	**9.0%**

- Essential
- Important
- Depends (if applicable, please provide more information in the text box below)
- Unimportant
- Should not be included
- Don't Know

The **senior leadership team** must only recommend and approve external training programmes and workshops (related to self-harm) for **school staff** that are: *endorsed by the local district health board's suicide prevention co-ordinator.*

Note: Suicide prevention co-ordinators are the local suicide prevention coordinators who are responsible for oversight and implementation of a local suicide prevention plan, connecting and enhancing communities to prevent suicide in their area.

	**Essential + Important**	**unimportant + not include**
Professionals Panel	**52.0%**	**8.0%**
Rangatahi Panel	**77.3%**	**4.5%**

Please rate the re-worded statement below:

The designated team should seek advice from their local suicide prevention coordinator about recommended training programmes and workshops on self-harm for school staff.

- Essential
- Important
- Depends (if applicable, please provide more information in the text box below)

- Unimportant
- Should not be included
- Don't Know

The **designated team** (once trained themselves) must provide training to **all staff** that is focused on self-harm and wellbeing and outlines; *when and how to meaningfully engage **whānau** of a **student** who is engaged in self-harm.*
Note: this in the context of designated team members that are trained themselves and competent to do this.

	**Essential + Important**	**unimportant + not include**
Professionals Panel	**68.0%**	**16.0%**
Rangatahi Panel	**95.5%**	**0.0%**

- Essential
- Important
- Depends (if applicable, please provide more information in the text box below)

- Unimportant
- Should not be included
- Don't Know

The **designated team** (once trained themselves) must provide training to **all staff** that is focused on self-harm and wellbeing and outlines; *the evidence-based facts of self-harm, in the hopes of reducing stigma and myths surrounding self-harm and mental health.*

Note: this in the context of designated team members that are trained themselves and competent to do this.

	Essential + Important	unimportant + not include
Professionals Panel	76.0	8.0
Rangatahi Panel	86.4	0.0

- Essential
- Important
- Depends (if applicable, please provide more information in the text box below)
- Unimportant
- Should not be included
- Don't Know

Please leave any thoughts or comments about this section below:

Training: Knowledge

Training: Required Knowledge and Awareness

The following statements relate to knowledge and awareness that **all staff** need, in order to support **students** who self-harm. This knowledge and awareness relates directly to self-harm and other factors that are associated with self-harm and the wellbeing of **students**.

Please rate the following statements:

All school staff must have the cultural knowledge and awareness to engage with **students** in a culturally safe way that aligns with the **student** and their **whānau** cultural worldview.

	Essential + Important	unimportant + not include
Professionals Panel	92.0%	0.0%
Rangatahi Panel	77.3%	0.0%

- Essential

○ Important

○ Depends (if applicable, please provide more information in the text box below)

○ Unimportant

○ Should not be included

○ Don't Know

All school staff must attend training opportunities that will enhance their knowledge and awareness of; *the relationship between self-harm and other important related issues and risk factors (e.g., bullying, substance use, trauma, substance use, etc.).*
Note: these training opportunities might be those provided by the designated team.

	Essential + Important	unimportant + not include
Professionals Panel	**76.0%**	**4.0%**
Rangatahi Panel	**81.8%**	**0.0%**

○ Essential

○ Important

○ Depends (if applicable, please provide more information in the text box below)

○ Unimportant

○ Should not be included

○ Don't Know

All school staff must attend training opportunities that will enhance their knowledge and awareness of; *when and how to meaningfully engage **whānau** of a **student** who is engaged in self-harm.*
Note: these training opportunities might be those provided by the designated team.

	Essential + Important	unimportant + not include
Professionals Panel	**68.0%**	**12.0%**
Rangatahi Panel	**86.4%**	**0.0%**

○ Essential

○ Important

○ Depends (if applicable, please provide more information in the text box below)

○ Unimportant

○ Should not be included

○ Don't Know

The **designated team** must know; *<u>how the mental health system in Aotearoa/New Zealand is structured and how it operates.</u>*
Note: this does not mean an in depth knowledge, but it includes knowledge that helps the designated team to know when and how to make a referral when specialist support is required.

	Essential + Important	unimportant + not include
Professionals Panel	**64.0%**	**12.0%**
Rangatahi Panel	**90.9%**	**0.0%**

○ Essential

○ Important

○ Depends (if applicable, please provide more information in the text box below)

○ Unimportant

○ Should not be included

○ Don't Know

All school staff must be aware of current national and local mental health campaigns that address mental health stigma (e.g. Voices of Hope: Speak Your Mind, Like Minds Like Mine, etc).
Note: This is not an in depth knowledge, but knowledge of the potential pros and cons of the various mental health campaigns.

	Essential + Important	unimportant + not include
Professionals Panel	**64.0%**	**8.0%**
Rangatahi Panel	**77.3%**	**9.1%**

○ Essential

○ Important

○ Depends (if applicable, please provide more information in the text box below)

○ Unimportant

○ Should not be included

○ Don't Know

As part of health and safety, **all school staff** must be aware of the potential risks and hazards within the school's physical environment in order to minimise and monitor risk in relation to self-harm (e.g., doors that lock, classroom equipment that are poisonous or harmful, etc.).

Note: Restricting access to self-harm methods is an evidenced-based suicide prevention measure.

	Essential + Important	unimportant + not include
Professionals Panel	**76.0%**	**0.0%**
Rangatahi Panel	**77.3%**	**4.5%**

- Essential
- Important
- Depends (if applicable, please provide more information in the text box below)
- Unimportant
- Should not be included
- Don't Know

Teachers and the designated team, must be aware that **teachers** (and in some cases students) can choose their own teaching materials and resources (e.g. media, literature), and that some of these materials and resources can be inadvertently triggering or distressing to **students**.

	Essential + Important	unimportant + not include
Professionals Panel	**92.0%**	**0.0%**
Rangatahi Panel	**77.3%**	**13.6%**

- Essential
- Important
- Depends (if applicable, please provide more information in the text box below)
- Unimportant
- Should not be included
- Don't Know

All school staff must be aware of their own cultural knowledge, values and beliefs, and must use the ongoing support of cultural advisors, and training opportunities to enhance their cultural competence. Note: It is important that staff are able to engage with students in a way that makes them feel that their culture is valued and that they are safe.

	Essential + Important	unimportant + not include
Professionals Panel	**96.0%**	**0.0%**
Rangatahi Panel	**68.2%**	**9.0%**

- Essential

○ Important

○ Depends (if applicable, please provide more information in the text box below)

○ Unimportant

○ Should not be included

○ Don't Know

The **designated team** must be aware of current research on self-harm, and the most effective ways of supporting **students** who self-harm by: *reading up-to-date literature.*

	Essential + Important	unimportant + not include
Professionals Panel	**72.0%**	**8.0%**
Rangatahi Panel	**63.6%**	**9.1%**

○ Essential

○ Important

○ Depends (if applicable, please provide more information in the text box below)

○ Unimportant

○ Should not be included

○ Don't Know

The **designated team** must be aware of the pressures **teaching staff** experience, by engaging in conversations with their co-workers to better understand the roles and experiences of **teaching staff**.
Note: having a school culture where various staff members are aware of and supportive of each other in their roles, helps to ensure the wellbeing of students

	Essential + Important	unimportant + not include
Professionals Panel	**72.0%**	**12.0%**
Rangatahi Panel	**90.9%**	**4.5%**

○ Essential

○ Important

○ Depends (if applicable, please provide more information in the text box below)

○ Unimportant

○ Should not be included

○ Don't Know

The **teaching staff** must be aware of the pressures the **designated team** experience, by engaging in conversations with their co-workers to better understand the roles and experiences of the **designated team**.
Note: having a school culture where various staff members are aware of and supportive of each other in their roles, helps to ensure the wellbeing of students

	Essential + Important	unimportant + not include
Professionals Panel	**72.0%**	**8.0%**
Rangatahi Panel	**90.9%**	**4.5%**

○ Essential

○ Important

○ Depends (if applicable, please provide more information in the text box below)

○ Unimportant

○ Should not be included

○ Don't Know

Please rate the following new statement:

When endorsing a campaign or inviting guest speakers to the school that includes mental health content (e.g., self-harm, abuse, suicide, substance use) you must consult with your local suicide prevention co-ordinator or child and adolescent community mental health service.

○ Essential

○ Important

○ Depends (if applicable, please provide more information in the text box below)

○ Unimportant

○ Should not be included

○ Don't Know

Please leave any thoughts or comments about this section below:

Training: Skills

Training: Required Skills

The following statements relate to skills that **all staff** need in order to support **students** who self-harm. These skills relate directly to self-harm, but may also be associated with other skills that are indirectly related to supporting **students** who self-harm.

Please rate the following statements:

The **designated team** must identify and engage in training opportunities that will provide them with the *skills* needed to: *complete a psycho-social assessment.*
Note: there are good examples of various professionals being trained to do psychosocial-assessment

	**Essential + Important**	**unimportant + not include**
Professionals Panel	**70.8%**	**16.6%**
Rangatahi Panel	**81.8%**	**4.5%**

- Essential
- Important
- Depends (if applicable, please provide more information in the text box below)
- Unimportant
- Should not be included
- Don't Know

The **designated team** must identify and engage in training opportunities that will provide them with the *skills* needed to; *manage and support **students** with difficulties related to self-harm (e.g., substance use, mental health difficulties, etc.).*

	**Essential + Important**	**unimportant + not include**
Professionals Panel	**79.2%**	**8.4%**
Rangatahi Panel	**95.5%**	**0.0%**

- Essential
- Important
- Depends (if applicable, please provide more information in the text box below)
- Unimportant
- Should not be included
- Don't Know

All School staff must receive training to equip them with *skills* to: *discuss emotionally distressing issues with **students**.*

	Essential + Important	unimportant + not include
Professionals Panel	**79.2%**	**4.2%**
Rangatahi Panel	**86.4%**	**4.5%**

- Essential
- Important
- Depends (if applicable, please provide more information in the text box below)
- Unimportant
- Should not be included
- Don't Know

All School staff must receive training to equip them with *skills* to: *support a **student** who is acutely distressed.*

	Essential + Important	unimportant + not include
Professionals Panel	**79.2%**	**4.2%**
Rangatahi Panel	**86.4%**	**0.0%**

- Essential
- Important
- Depends (if applicable, please provide more information in the text box below)
- Unimportant
- Should not be included
- Don't Know

The **school principal** must receive training that will equip them with the *skills* needed to support **school staff** to perform their roles and responsibilities in supporting **students**.

	Essential + Important	unimportant + not include
Professionals Panel	**79.2%**	**8.3%**
Rangatahi Panel	**90.9%**	**0.0%**

- Essential
- Important

Depends (if applicable, please provide more information in the text box below)

Unimportant

Should not be included

Don't Know

The **designated team** must be trained in using screening tools that are evidence-based and useful for identifying risk and protective factors associated with self-harm.
NOTE: screening tools can include pen and paper tools, as well as face to face assessment. The designated team members must be trained in the approach they want to use

	Essential + Important	unimportant + not include
Professionals Panel	**75.0%**	**12.5%**
Rangatahi Panel	**77.3%**	**4.5%**

Essential

Important

Depends (if applicable, please provide more information in the text box below)

Unimportant

Should not be included

Don't Know

Please leave any thoughts or comments about this section below:

Supervision

Supervision

The following statements relate to professional supervision, and how it relates to how **school staff** can support **students** who self-harm.

Key terms introduced in this section:
Professional Supervision: the ongoing process of professional support from a qualified supervisor that focuses on enhancing knowledge and understanding, and skill relating to professional practice and the ethical requirements of the profession.

Cultural Supervision: A formal supervision relationship that aims to enhance the awareness, knowledge and skills needed to work within the cultural context of students (clients) and their whānaua.
Peer supervision: A formal process where a group of co-workers or professionals who have the same or similar roles meet to discuss professional issues with each other.

Please rate the following statements:

The **designated team** must receive professional supervision, and use it as an opportunity to consult, seek advice, engage in professional development, reflect on their own practice, and as a means of self-care.

	Essential + Important	unimportant + not include
Professionals Panel	**79.2%**	**4.2%**
Rangatahi Panel	**100.0%**	**0.0%**

- Essential
- Important
- Depends (if applicable, please provide more information in the text box below)
- Unimportant
- Should not be included
- Don't Know

The **designated team** must attend peer supervision, and use it as an opportunity to share knowledge, seek advice and as a form of self-care.
Note: when it is possible for staff to attend

	Essential + Important	unimportant + not include
Professionals Panel	**79.2%**	**4.2%**
Rangatahi Panel	**90.9%**	**0.0%**

- Essential
- Important
- Depends (if applicable, please provide more information in the text box below)
- Unimportant
- Should not be included
- Don't Know

Please rate the following new statements:

Supervision must occur a frequency that suits the staff members needs and expertise (e.g., school teacher with no counselling experience versus the school guidance counsellor)

- Essential
- Important
- Depends (if applicable, please provide more information in the text box below)
- Unimportant
- Should not be included
- Don't Know

All school staff must have access to supervision when working with students who self-harm.

- Essential
- Important
- Depends (if applicable, please provide more information in the text box below)
- Unimportant
- Should not be included
- Don't Know

Please leave any thoughts or comments about this section below:

Documentation

Documentation

The following statements relate to the documentation processes that may be required and necessary to support **students** who self-harm.

Please rate the following new statements:

Where an electronic record system is being used for notes about a student's welfare, these notes need to be kept separate from the student's academic information. Where an electronic system isn't used or a paper-based system is used in addition, this information needs to be kept separate from the academic information and securely stored in a locked cabinet or room.

- Essential
- Important
- Depends (if applicable, please provide more information in the text box below)
- Unimportant
- Should not be included
- Don't Know

In alignment with the principles of confidentiality, new episodes of self-harm must be shared with the relevant external providers (e.g., Child and adolescent community mental health case worker or intake team, GP).

- Essential
- Important
- Depends (if applicable, please provide more information in the text box below)
- Unimportant
- Should not be included
- Don't Know

Please leave any thoughts or comments about this section below:

Communication and Collaboration

Communication and Collaboration

The following statements relate to communication and collaboration between **all school staff**, the **student**, their **whānau**, and other relevant services and groups. It outlines the communication needed in order to support **students** who self-harm, including expectations around information sharing and confidentiality.

Please re-rate the following statements:

The **designated team** must meet regularly to debrief and to check-in with each other.

	**Essential + Important**	**unimportant + not include**

Professionals Panel	**75.0%**	**4.2%**
Rangatahi Panel	**100.0%**	**0.0%**

- Essential
- Important
- Depends (if applicable, please provide more information in the text box below)
- Unimportant
- Should not be included
- Don't Know

In instances where there is only one person in the **designated team,** they must; establish *a consult team to co-ordinate roles and plans (e.g., who is the most appropriate person to call* ***whānau****).*
Note: a consult team is a group who can provide support and advice, and could include principal, deputies, team leads, SENCO, etc)

	Essential + Important	unimportant + not include
Professionals Panel	**70.8%**	**8.3%**
Rangatahi Panel	**81.8%**	**4.5%**

- Essential
- Important
- Depends (if applicable, please provide more information in the text box below)
- Unimportant
- Should not be included
- Don't Know

In instances where there is only one person in the **designated team,** they must; establish a consult team to debrief and to check-in with each other.
Note: a consult team is a group who can provide support and advice, and could include principal, deputies, team leads, SENCO, etc.)

	Essential + Important	unimportant + not include
Professionals Panel	**79.2%**	**12.5%**
Rangatahi Panel	**90.9%**	**0.0%**

- Essential
- Important

○ Depends (if applicable, please provide more information in the text box below)

○ Unimportant

○ Should not be included

○ Don't Know

The **designated team** must establish a preferred referral method for internal referrals, and must ensure all staff are aware of this method (e.g., email, complete referral form, call and email, face to face conversation and email, etc).
Note: students (where appropriate) can be involved in establishing this process

	**Essential + Important**	**unimportant + not include**
Professionals Panel	**83.3%**	**4.2%**
Rangatahi Panel	**77.3%**	**9.0%**

○ Essential

○ Important

○ Depends (if applicable, please provide more information in the text box below)

○ Unimportant

○ Should not be included

○ Don't Know

The **designated team** may have concerns about a **student**, or feel that other **staff members** can support a **student** who self-harms. *If this is the case they must;*

- Work with the **student** to identify what they may need from **other school staff**
- Tell the **student** what information will be passed on and to whom
- Inform only the **staff** who are directly involved with the **student**

	**Essential + Important**	**unimportant + not include**
Professionals Panel	**79.2%**	**4.2%**
Rangatahi Panel	**90.9%**	**0.0%**

○ Essential

○ Important

Depends (if applicable, please provide more information in the text box below)

Unimportant

Should not be included

Don't Know

The **designated team** may have concerns about a **student**, or feel that **other staff members** can support a **student** who self-harms. If this is the case they must; o*only disclose information related to the support the **student** needs but not detail about issues or events that the **student** has disclosed, unless requested by the **student**.*

	**Essential + Important**	**unimportant + not include**
Professionals Panel	**70.8%**	**8.3%**
Rangatahi Panel	**90.9%**	**0.0%**

Essential

Important

Depends (if applicable, please provide more information in the text box below)

Unimportant

Should not be included

Don't Know

If the **nurse** is not part of the **designated team,** the **designated team** must ensure the **nurse** or other **school medical staff** are informed of any **students** who may be a risk to themselves.
Note: with the students consent and awareness

	**Essential + Important**	**unimportant + not include**
Professionals Panel	**75.0%**	**4.2%**
Rangatahi Panel	**86.4%**	**4.5%**

Essential

Important

Depends (if applicable, please provide more information in the text box below)

Unimportant

Should not be included

Don't Know

The **designated team** must be able to identify **students** who self-harm and therefore must be aware of all **students** who self-harm.

	Essential + Important	unimportant + not include
Professionals Panel	**66.7%**	**8.3%**
Rangatahi Panel	**77.3%**	**9.1%**

- Essential
- Important
- Depends (if applicable, please provide more information in the text box below)
- Unimportant
- Should not be included
- Don't Know

To gain an understanding of the services available in the community, the **designated team** must; *determine how the service operates.*

	Essential + Important	unimportant + not include
Professionals Panel	**79.2%**	**4.2%**
Rangatahi Panel	**90.9%**	**0.0%**

- Essential
- Important
- Depends (if applicable, please provide more information in the text box below)
- Unimportant
- Should not be included
- Don't Know

The **designated team** must ensure they inform **students** and their **whānau** that the information shared with an individual **designated team member** might also be shared with other members of the **designated team.** But that the information will be confidential to the team, and therefore will not be discussed with others outside of the **designated** team, unless there are safety concerns.

	Essential + Important	unimportant + not include
Professionals Panel	**79.2%**	**8.3%**
Rangatahi Panel	**95.5%**	**0.0%**

- Essential
- Important
- Depends (if applicable, please provide more information in the text box below)
- Unimportant
- Should not be included
- Don't Know

Please rate the following new statements:
The designated team should identify and connect with their local Suicide Prevention Coordinator (SPC) for advice and support relevant to their local suicide prevention plan and their school.

- Essential
- Important
- Depends (if applicable, please provide more information in the text box below)
- Unimportant
- Should not be included
- Don't Know

Students should be made aware that confidentiality will be maintained within the **designated team**, rather than only the individual they are talking to.

- Essential
- Important
- Depends (if applicable, please provide more information in the text box below)
- Unimportant
- Should not be included
- Don't Know

Please leave any thoughts or comments about this section below:

Wellbeing and Self-care

Wellbeing and Self-care

The following statements relate to self-care practices that are required in order for **school staff** to support **students** who self-harm.

Please rate the following statements:

The **designated team** must maintain professional boundaries when working with **students** in order to take care of their own wellbeing, by: *having clear working hours and days, and only seeing **students** during school hours (thus not during holiday, after school).*

Note: young people say they find it helpful to have access to support when needed. Outside of work hours a school professional's availability is limited, therefore it is the responsibility of the designated team to ensure that the student has access to contacts and support outside of school. The aim should be to increase the support available outside of school, beyond one person (e.g., helplines, trusted adults in whanau, etc).

	Essential + Important	unimportant + not include
Professionals Panel	**79.2%**	**4.2%**
Rangatahi Panel	**77.3%**	**4.5%**

- Essential
- Important
- Depends (if applicable, please provide more information in the text box below)

- Unimportant
- Should not be included
- Don't Know

Please rate the following new statements:

All school staff, particularly those involved in the welfare of students (e.g. Dean, SENCO, principals, deputy, etc) must seek support, supervision, and a work-life balance.

- Essential
- Important
- Depends (if applicable, please provide more information in the text box below)

- Unimportant
- Should not be included
- Don't Know

Please leave any thoughts or comments about this section below:

Initial Response: History of Self-Harm

Initial response: history of self-harm

The following section relates to the roles and responsibilities of various **staff members** and **teams** in the instance where a **student** is identified, who has a history of self-harm (this may include self-harm that has occurred in the last day, week, month, year or years). Thus this refers to the steps **staff** might take if a **student** self-harmed in the past.

For this section "the past" or a "history" refers to any self-harming behaviours that occurred before the present moment and therefore can be minutes, hours, days, months or years in the past.

Identification of the **student** may occur, through observation or disclosure directly to a **staff member** or through a disclosure by a **peer**.

Clarification: The statements in the following sections focus on the initial responses to self-harm. Although it would be ideal for the staff member present in that moment to respond in that moment, there might be circumstances that mean an alternative staff member may need to be called to support the student instead (E.g., the staff member might be triggered, the student might prefer another member of staff). In most cases one staff member will be allocated to respond to a student who is self-harming

Please re-rate the following statements:

If a **peer** informs **staff** of another **student's** self-harm history the staff member must: *ask the **peer** if they know when the most recent time is that the **student** has self-harmed.*

	Essential + Important	unimportant + not include
Professionals Panel	**66.7%**	**8.3%**
Rangatahi Panel	**72.7%**	**9.0%**

- Essential
- Important
- Depends (if applicable, please provide more information in the text box below)
- Unimportant
- Should not be included
- Don't Know

If a **peer** informs **staff** of another **student's** self-harm history the staff member must; *ask the **peer** for as much contextual information (e.g., when did this happen, who else knows, etc) that the **peer** can provide.*

Note: this could be verbal or written communication depending on what the peer is comfortable with.

	Essential + Important	unimportant + not include
Professionals Panel	**62.5%**	**8.3%**

Rangatahi Panel	**81.8%**	**4.5%**

- Essential
- Important
- Depends (if applicable, please provide more information in the text box below)
- Unimportant
- Should not be included
- Don't Know

If a **peer** informs **staff** of another **student's** self-harm history the staff member must; *ensure the **designated team** isis made aware of who the **peer** is.* *Note: this item is included so that the designated team can check in with the peer about their wellbeing*

	Essential + Important	unimportant + not include
Professionals Panel	**50.0%**	**4.2%**
Rangatahi Panel	**77.3%**	**9.0%**

- Essential
- Important
- Depends (if applicable, please provide more information in the text box below)
- Unimportant
- Should not be included
- Don't Know

If a **peer** informs **staff** of another **student's** self-harm history, the **designated team** must; *meet with the **peer** and discuss whether the **peer** needs any support.*

	Essential + Important	unimportant + not include
Professionals Panel	**79.2%**	**4.2%**
Rangatahi Panel	**95.5%**	**0.0%**

- Essential
- Important
- Depends (if applicable, please provide more information in the text box below)

- Unimportant
- Should not be included
- Don't Know

If **any staff member** identifies a **student** who self-harmed (through observation, disclosure or screening), that **staff member** must: *avoid asking to see a **student's** self-harm injuries as proof of self-harm, but do ask when the last time was that the **student** self-harmed*
Note: asking about when the last time occurred will help establish whether if medical support is needed

	Essential + Important	unimportant + not include
Professionals Panel	**75.0%**	**4.2%**
Rangatahi Panel	**81.8%**	**0.0%**

- Essential
- Important
- Depends (if applicable, please provide more information in the text box below)

- Unimportant
- Should not be included
- Don't Know

If **any staff member** identifies a **student** who self-harmed (through observation, disclosure or screening), that **staff member** must: *offer access to medical attention with explanation as to why (e.g., sometimes the injuries can get infected and need to be cleaned and bandaged).*

	Essential + Important	unimportant + not include
Professionals Panel	**79.2%**	**4.2%**
Rangatahi Panel	**95.5%**	**0.0%**

- Essential
- Important
- Depends (if applicable, please provide more information in the text box below)

- Unimportant
- Should not be included
- Don't Know

If the **student** consents to medical attention, the **staff member** must ensure the **student** receives medical intervention as soon as possible, and must ensure the **student** is not left

alone.
Note: for a recent wound.

	**Essential + Important**	**unimportant + not include**
Professionals Panel	**75.0%**	**4.2%**
Rangatahi Panel	**90.9%**	**0.0%**

- ○ Essential
- ○ Important
- ○ Depends (if applicable, please provide more information in the text box below)

- ○ Unimportant
- ○ Should not be included
- ○ Don't Know

If **any staff member** identifies a **student** who self-harmed (through observation, disclosure or screening), that **staff member** must: *follow up with the **designated team** to ensure that the **student** has been seen.*
Note: this should be in the context of a process where there is a process in place that ensures good communication between the designated team and staff, this ensures that the student is receiving follow-up care.

	**Essential + Important**	**unimportant + not include**
Professionals Panel	**79.2%**	**8.4%**
Rangatahi Panel	**90.9%**	**4.5%**

- ○ Essential
- ○ Important
- ○ Depends (if applicable, please provide more information in the text box below)

- ○ Unimportant
- ○ Should not be included
- ○ Don't Know

Please leave any thoughts or comments about this section below:

Initial Response: Self-harm in the Moment

Initial response: self-harm in the moment

The following section relate to the roles and responsibilities of various **staff members** and **teams** in the instance where a **student** is ***identified while engaging in self-harming behaviour*** e.g. in the class-room. Thus, this section covers actions that may be taken if a **student** is ***actively self-harming in the present moment***.
Identification of the **student** may occur, through observation or disclosure directly to a **staff member** or through a disclosure by a **peer**.

Clarification: The statements in the following sections focus on the initial responses to self-harm. Although it would be ideal for the staff member present in that moment to respond in that moment, there might be circumstances that mean an alternative staff member may need to be called to support the student instead (E.g., the staff member might be triggered, the student might prefer another member of staff). In most cases one staff member will be allocated to respond to a student who is self-harming

Please re-rate the following statements:

If a **peer** informs **staff** that another **student** is self-harming in that moment, the **staff member** must; *ensure the relevant **designated team member** is informed of who the **peer** is.*

	Essential + Important	unimportant + not include
Professionals Panel	**79.2%**	**4.2%**
Rangatahi Panel	**81.8%**	**9.0%**

- ○ Essential
- ○ Important
- ○ Depends (if applicable, please provide more information in the text box below)
- ○ Unimportant
- ○ Should not be included
- ○ Don't Know

If a **peer** informs **staff** that another **student** is self-harming in that moment, the **designated team** must; *meet with the **peer** and ensure the **peer** is provided with support.*

	Essential + Important	unimportant + not include
Professionals Panel	**79.2%**	**4.2%**
Rangatahi Panel	**90.9%**	**4.5%**

- ○ Essential
- ○ Important
- ○ Depends (if applicable, please provide more information in the text box below)

- Unimportant
- Should not be included
- Don't Know

If a **peer** informs **staff** that another **student** is self-harming in that moment, the **staff member** must; *go to where the **student** is located immediately.*
Note: this is in the context of gaining support from others if required

	Essential + Important	unimportant + not include
Professionals Panel	**79.2%**	**0.0%**
Rangatahi Panel	**68.2%**	**4.5%**

- Essential
- Important
- Depends (if applicable, please provide more information in the text box below)

- Unimportant
- Should not be included
- Don't Know

The **staff member** who identified the **student**, must discretely intervene by: *removing the means used by the **student** to self-harm.*
Note: when safe to do so (i.e., will not escalate the situation), in which case knowing the whereabouts so of the means is important to ascertain

	Essential + Important	unimportant + not include
Professionals Panel	**66.7%**	**4.2%**
Rangatahi Panel	**77.3%**	**4.5%**

- Essential
- Important
- Depends (if applicable, please provide more information in the text box below)

- Unimportant
- Should not be included
- Don't Know

The **staff member** who identified the **student**, must discretely intervene by; *directing the **student** to come with them to see the **medical/first aid staff member** if medical attention is required.*

	Essential + Important	unimportant + not include

Professionals Panel	**75.0%**	**8.3%**
Rangatahi Panel	**95.5%**	**0.0%**

- Essential
- Important
- Depends (if applicable, please provide more information in the text box below)

- Unimportant
- Should not be included
- Don't Know

The **staff member** who identified the **student**, must discretely intervene by: *directing the **student** to come with them to see the **designated team**, once medical intervention has been sought.*
Note: If possible can also call the designated person to the location

	Essential + Important	unimportant + not include
Professionals Panel	**62.5%**	**4.2%**
Rangatahi Panel	**90.9%**	**9.1%**

- Essential
- Important
- Depends (if applicable, please provide more information in the text box below)

- Unimportant
- Should not be included
- Don't Know

Please rate the following new statements:
If a student indicated that they need particular strategies such as physical reassurance (e.g., hug from a close friends), or a distraction (e.g., gaming, music, etc), the staff member should facilitate this, as long as it is safe.

- Essential
- Important
- Depends (if applicable, please provide more information in the text box below)

- Unimportant

○ Should not be included

○ Don't Know

Please leave any thoughts or comments about this section below:

Responding to events that occur publicly

Responding to events that occur publicly

The following statements related to **staff** responses in the case that the self-harm is taking place in a visible/public way.

Please rate the following statements:

The staff member who identified the **student**, must de-escalate the situation by: *remaining at a safe distance.*
Note: a safe distance is one where you are not encroaching on the student's space, that you are not standing over the student but that you are close enough to have a calm conversation.

	Essential + Important	unimportant + not include
Professionals Panel	**70.8%**	**4.2%**
Rangatahi Panel	**86.4%**	**4.5%**

○ Essential

○ Important

○ Depends (if applicable, please provide more information in the text box below)

○ Unimportant

○ Should not be included

○ Don't Know

The **staff member** who identified the **student**, must de-escalate the situation by; *trying to remove the means used by the **student** to self-harm.*
Note: when safe to do so (i.e., will not escalate the situation), in which case knowing the whereabouts of the means is important to ascertain

	Essential + Important	unimportant + not include
Professionals Panel	**70.8%**	**4.2%**

Rangatahi Panel	**90.9%**	**0.0%**

- Essential
- Important
- Depends (if applicable, please provide more information in the text box below)
- Unimportant
- Should not be included
- Don't Know

The **staff member** who identified the **student**, must de-escalate the situation by; *giving gentle clear instructions to take some deep breaths.*

	Essential + Important	unimportant + not include
Professionals Panel	**75.0%**	**4.2%**
Rangatahi Panel	**86.4%**	**0.0%**

- Essential
- Important
- Depends (if applicable, please provide more information in the text box below)
- Unimportant
- Should not be included
- Don't Know

The **staff member** must; *send **other students** away (e.g. send to usual assembly point).*

	Essential + Important	unimportant + not include
Professionals Panel	**75.0%**	**0.0%**
Rangatahi Panel	**95.5%**	**0.0%**

- Essential
- Important
- Depends (if applicable, please provide more information in the text box below)
- Unimportant
- Should not be included

○ Don't Know

Please leave any thoughts or comments about this section below:

Designated Team Response

Designated Team Response

The following items relate to the way in which the **designated team** should respond following the identification of a **student** who self-harms. The **designated person** may have identified the **student** themselves or may have received a referral or crisis call from another **school staff member.** The statements below are in the context where all designated team members receive the required training.

Please rate the following statements:

The staff member to whom the student initially presented, may have a better relationship with the student, and as such they can offer to facilitate the connection between the student and the designated team member; by offering to sit in for part of the meeting.

	Essential + Important	unimportant + not include
Professionals Panel	**73.9%**	**0.0%**
Rangatahi Panel	**100.0%**	**0.0%**

○ Essential
○ Important
○ Depends (if applicable, please provide more information in the text box below)

○ Unimportant
○ Should not be included
○ Don't Know

Once the **designated team member** feels they have built rapport with the **student** they must: *carry out a psychosocial assessment.*

	Essential + Important	unimportant + not include
Professionals Panel	**73.9%**	**4.3%**
Rangatahi Panel	**90.9%**	**4.5%**

- Essential
- Important
- Depends (if applicable, please provide more information in the text box below)
- Unimportant
- Should not be included
- Don't Know

In order to understand the **student's** self-harm and how to support the **student**, the **designated team member** must explore: *the nature of the **student's** self-harming behaviour (most recent, when and how).*

	**Essential + Important**	**unimportant + not include**
Professionals Panel	**73.9%**	**4.3%**
Rangatahi Panel	**90.9%**	**0.0%**

- Essential
- Important
- Depends (if applicable, please provide more information in the text box below)
- Unimportant
- Should not be included
- Don't Know

The **designated team member**, must not suggest that the **student** should stop self-harming (e.g., tell the student to stop), but rather must help the **student** identify other coping strategies and ways of enhancing wellbeing (e.g., time with family, exercise, mindfulness, etc.).
Note: This is in acknowledgement that for some young people this is their only coping strategy, and until they develop other strategies it may be unhelpful to suggest immediate cessation. Note this does **not** mean encouraging self-harm, but suggest a change in focus to working with the students to develop other coping strategies and means of enhancing wellbeing. A harm minimisation approach is a helpful framework to guide intervention

	**Essential + Important**	**unimportant + not include**
Professionals Panel	**69.6%**	**4.3%**
Rangatahi Panel	**90.9%**	**4.5%**

- Essential
- Important

○ Depends (if applicable, please provide more information in the text box below)

○ Unimportant

○ Should not be included

○ Don't Know

Please leave any thoughts or comments about this section below:

Contacting Whānau

Contacting whānau

The following statements relate to the actions that need to be taken when contacting the **whānau** of a **student** who self-harms or self-harmed.

Please rate the following statements:

The **designated team member** must first encourage the **student** to inform their **whānau** of the **student's** self-harm.
Note: Following an assessment of safety, and if the self-harm is current. Whānau should include any safe adult who can provide support (e.g., older cousin, youth pastor, older siblings, etc.)

	Essential + Important	unimportant + not include
Professionals Panel	**73.9%**	**0.0%**
Rangatahi Panel	**81.8%**	**0.0%**

○ Essential

○ Important

○ Depends (if applicable, please provide more information in the text box below)

○ Unimportant

○ Should not be included

○ Don't Know

The **designated team member** must inform the **student** that it is school protocol for the **designated team member** to inform the **student's whānau** of the **student's** self-harm,

even if the **student** does not consent.
Note: Following an assessment of safety, and if the self-harm is current. Whānau should include any safe adult who can provide support (e.g., older cousin, youth pastor, older siblings, etc.)

	Essential + Important	unimportant + not include
Professionals Panel	**73.9%**	**13.0%**
Rangatahi Panel	**72.7%**	**4.5%**

- Essential
- Important
- Depends (if applicable, please provide more information in the text box below)
- Unimportant
- Should not be included
- Don't Know

The **designated team member** (or most **appropriate person**, e.g., cultural advisor , youth worker, Chaplin, etc) must arrange for a face to face meeting if the **whānau** is interested.
Note: Following an assessment of safety, and if the self-harm is current. Whānau should include any safe adult who can provide support (e.g., older cousin, youth pastor, older siblings, etc.)

	Essential + Important	unimportant + not include
Professionals Panel	**78.3%**	**0.0%**
Rangatahi Panel	**81.8%**	**0.0%**

- Essential
- Important
- Depends (if applicable, please provide more information in the text box below)
- Unimportant
- Should not be included
- Don't Know

The **designated team member** must inform the **whānau** of the school's self-harm policy.

	Essential + Important	unimportant + not include
Professionals Panel	**73.9%**	**4.3%**
Rangatahi Panel	**81.8%**	**0.0%**

- Essential
- Important
- Depends (if applicable, please provide more information in the text box below)

- Unimportant
- Should not be included
- Don't Know

The **designated team member** must inform the **whānau** of the safety plan.
Note: Following an assessment of safety, and if the self-harm is current. Whānau could include any safe adult who can provide support (e.g., older cousin, youth pastor, older siblings, etc.). It is important that the person or people providing support outside of school can support in a way that is consistent with the plan that is developed by the student and designated team.

	Essential + Important	unimportant + not include
Professionals Panel	**78.3%**	**4.3%**
Rangatahi Panel	**72.7%**	**0.0%**

- Essential
- Important
- Depends (if applicable, please provide more information in the text box below)

- Unimportant
- Should not be included
- Don't Know

The **designated team member** must inform the **student's whānau** of options for referral.
Note: Following an assessment of safety, and if the self-harm is current. Whānau should include any safe adult who can provide support (e.g., older cousin, youth pastor, older siblings, etc.). This should also occur in collaboration with the student.

	Essential + Important	unimportant + not include
Professionals Panel	**95.7%**	**0.0%**
Rangatahi Panel	**77.3%**	**0.0%**

- Essential
- Important

○ Depends (if applicable, please provide more information in the text box below)

○ Unimportant

○ Should not be included

○ Don't Know

The **designated team member** must involve the **student's whānau** in the decision about whether and where to refer the **student.**
Note: Following an assessment of safety, and if the self-harm is current. Whānau should include any safe adult who can provide support (e.g., older cousin, youth pastor, older siblings, etc.).

	Essential + Important	unimportant + not include
Professionals Panel	**91.3%**	**0.0%**
Rangatahi Panel	**63.6%**	**4.5%**

○ Essential

○ Important

○ Depends (if applicable, please provide more information in the text box below)

○ Unimportant

○ Should not be included

○ Don't Know

The **designated team member** must schedule a follow-up meeting with **whānau** and **student** after the initial meeting. This typically occurs 1-2 weeks and no later than 1 month after the school detects a self-harm incident.

	Essential + Important	unimportant + not include
Professionals Panel	**78.3%**	**4.3%**
Rangatahi Panel	**90.9%**	**0.0%**

○ Essential

○ Important

○ Depends (if applicable, please provide more information in the text box below)

○ Unimportant

○ Should not be included

○ Don't Know

Please rate the following new statements:
Before contacting whanau, the designated person should assess the safety of disclosing to the whanau.

○ Essential

○ Important

○ Depends (if applicable, please provide more information in the text box below)

○ Unimportant

○ Should not be included

○ Don't Know

When a disclosure is going to happen, the designated team should support the student by discussing with them what information will be disclosed, who it will be disclosed, and how the process of including whānau will happen.

○ Essential

○ Important

○ Depends (if applicable, please provide more information in the text box below)

○ Unimportant

○ Should not be included

○ Don't Know

Please leave any thoughts or comments about this section below:

Referrals

Referrals

The following statements relate to referring a **student** who self-harms to internal school support and external community services.

Please rate the following statements:

The **designated team member** must refer ***students*** to external services if the ***student***; *presents with chronic risk.*

	Essential + Important	unimportant + not include
Professionals Panel	**78.3%**	**0.0%**
Rangatahi Panel	**100.0%**	**0.0%**

- Essential
- Important
- Depends (if applicable, please provide more information in the text box below)
- Unimportant
- Should not be included
- Don't Know

The **designated team member** must refer **students** to external services if the **student;** *has limited protective factors.*

	Essential + Important	unimportant + not include
Professionals Panel	**69.6%**	**0.0%**
Rangatahi Panel	**100.0%**	**0.0%**

- Essential
- Important
- Depends (if applicable, please provide more information in the text box below)
- Unimportant
- Should not be included
- Don't Know

Along with making a referral to an external agency, the **designated team member** must make a phone call or send an email to the external agency to discuss; *any missing information/further assessment required.*

	Essential + Important	unimportant + not include
Professionals Panel	**78.3%**	**0.0%**
Rangatahi Panel	**95.5%**	**0.0%**

- Essential
- Important

Depends (if applicable, please provide more information in the text box below)

Unimportant

Should not be included

Don't Know

Along with making a referral to an external agency, the **designated team member** must make a phone call or send an email to the external agency *to discuss; wait-time/holding pattern.*

	**Essential + Important**	**unimportant + not include**
Professionals Panel	**73.9%**	**0.0%**
Rangatahi Panel	**81.8%**	**0.0%**

Essential

Important

Depends (if applicable, please provide more information in the text box below)

Unimportant

Should not be included

Don't Know

Please rate the following new statements:
The student must be involved in the decision about who they will receive intervention from (e.g., may prefer intervention from an external provider and they should have input into who that will be).

Essential

Important

Depends (if applicable, please provide more information in the text box below)

Unimportant

Should not be included

Don't Know

Please leave any thoughts or comments about this section below:

Ongoing Support

Ongoing support

The following statements relate to the ongoing support required by **students** and their **whānau**, after the initial response to identifying a **student** who self-harms.

Please rate the following statements:

Following the initial response, the **designated team member** must act as a liaison between:

- the **student**
- **whānau**
- **school staff** involved
- **peers**
- outside referral agents associated with the **student** as a result of the disclosure

Note: a co-ordinated and consistent approach is important across all settings

	Essential + Important	unimportant + not include
Professionals Panel	**78.3%**	**0.0%**
Rangatahi Panel	**90.0%**	**0.0%**

- Essential
- Important
- Depends (if applicable, please provide more information in the text box below)
- Unimportant
- Should not be included
- Don't Know

If needed and requested by the **whānau**, the **designated team member and the appropriate staff member** (e.g., kaumatua) must support **whānau** with adapting parenting approaches (e.g. developing empathic understandings) to support their distressed child. This could be offered in a group format and must consider the cultural context of parenting practices.

	Essential + Important	unimportant + not include
Professionals Panel	**73.9%**	**4.3%**
Rangatahi Panel	**86.4%**	**4.5%**

- Essential
- Important

- ○ Depends (if applicable, please provide more information in the text box below)
- ○ Unimportant
- ○ Should not be included
- ○ Don't Know

Please rate the following new statements:

The designated team must encourage the student and whānau to ask the external agency involved in the support of the student, to include the designated team in the treatment plan.

- ○ Essential
- ○ Important
- ○ Depends (if applicable, please provide more information in the text box below)
- ○ Unimportant
- ○ Should not be included
- ○ Don't Know

The designated team need to ensure that a safety plan is in place for students during school holidays and outside of school-hours, which clearly specifies who the student can reach out to for support during these times.

- ○ Essential
- ○ Important
- ○ Depends (if applicable, please provide more information in the text box below)
- ○ Unimportant
- ○ Should not be included
- ○ Don't Know

Safety plans must be reviewed and updated regularly, especially in anticipation of school holidays, to ensure potential risks are managed, and that students have contacts in case they need support during holidays.

- ○ Essential
- ○ Important

○ Depends (if applicable, please provide more information in the text box below)

○ Unimportant

○ Should not be included

○ Don't Know

Please leave any thoughts or comments about this section below:

Social Contagion

Social Contagion

The following statements relate to actions **school staff** can take in order to prevent or respond to social contagion of self-harm.

Key term(s) introduced in this section:

Social contagion: In this questionnaire social contagion refers to the social contagion of self-harm and other suicidal behaviours. Social contagion of self-harm is defined in this questionnaire as, the spread of self-harming behaviour following exposure to self-harming behaviour or suicide by others. This exposure can occur directly (e.g., family, peer groups or community) or indirectly (e.g., media).

Please rate the following statements:

First aid and medical staff must ensure that any tape, bandages or plasters used to cover self-harm injuries are not identifiable or stand out, therefore preventing it from becoming a symbol of self-harm. Note: for some young people it can be triggering

	Essential + Important	unimportant + not include
Professionals Panel	**73.9%**	**8.7%**
Rangatahi Panel	**72.7%**	**4.5%**

○ Essential

○ Important

○ Depends (if applicable, please provide more information in the text box below)

○ Unimportant

○ Should not be included

○ Don't Know

Please leave any thoughts or comments about this section below:

Resources

Resources

The following statements relate to the resources needed in schools in order for **staff** to support **students** who self-harm.

Please rate the following statements:

In a rural school, the **board of trustees** must hire a **pastoral care professional (e.g., counsellor**) who has the necessary skills to identify and manage self-harm.

	**Essential + Important**	**unimportant + not include**
Professionals Panel	**73.9%**	**4.3%**
Rangatahi Panel	**95.5%**	**0.0%**

Please rate the re-worded statement:

In Schools in areas where there are fewer or no external services that students can be referred to (including rural schools), the **board of trustees** must consider hiring a **pastoral care professional (e.g., counsellor**) who has the necessary skills to identify and manage self-harm.

- Essential
- Important
- Depends (if applicable, please provide more information in the text box below)
- Unimportant
- Should not be included
- Don't Know

Please rate the following new statements:

Leave arrangements for the designated team need to be flexible, to ensure the needs of the staff members are met.

- Essential
- Important

- ◯ Depends (if applicable, please provide more information in the text box below)

- ◯ Unimportant
- ◯ Should not be included
- ◯ Don't Know

Please leave any thoughts or comments about this section below:

Powered by Qualtrics
